# The effects of graded levels of calorie restriction: XI. Evaluation of the main hypotheses underpinning the life extension effects of CR using the hepatic transcriptome

**DOI:** 10.18632/aging.101269

**Published:** 2017-07-31

**Authors:** Davina Derous, Sharon E. Mitchell, Lu Wang, Cara L. Green, Yingchun Wang, Luonan Chen, Jing-Dong J. Han, Daniel E.L. Promislow, David Lusseau, Alex Douglas, John R. Speakman

**Affiliations:** ^1^ Institute of Biological and Environmental Sciences, University of Aberdeen, Aberdeen, Scotland, UK; ^2^ Centre for Genome Enabled Biology and Medicine, University of Aberdeen, Aberdeen, Scotland, UK; ^3^ State Key laboratory of Molecular Developmental Biology, Institute of Genetics and Developmental Biology, Chinese Academy of Sciences, Chaoyang, Beijing, China; ^4^ Chinese Academy of Sciences Key Laboratory of Computational Biology, Chinese Academy of Sciences-Max Planck Partner Institute for Computational Biology, Shanghai Institutes for Biological Sciences, Chinese Academy of Sciences, Shanghai, China; ^5^ Key laboratory of Systems Biology, Innovation Center for Cell Signaling Network, Institute of Biochemistry and Cell Biology, Shanghai Institute of Biological Sciences, Chinese Academy of Sciences, Shanghai, China; ^6^ Department of Pathology, University of Washington, Seattle, WA 98195, USA; ^7^ Department of Biology, University of Washington, Seattle, WA 98195, USA

**Keywords:** aging, calorie restriction, gene expression, liver, transcriptomics

## Abstract

Calorie restriction (CR) may extend longevity by modulating the mechanisms involved in aging. Different hypotheses have been proposed for its main mode of action. We quantified hepatic transcripts of male C57BL/6 mice exposed to graded levels of CR (0% to 40% CR) for three months, and evaluated the responses relative to these various hypotheses. Of the four main signaling pathways implied to be linked to the impact of CR on lifespan (insulin/insulin like growth factor 1 (IGF-1), nuclear factor-kappa beta (NF-ĸB), mechanistic target of rapamycin (mTOR) and sirtuins (SIRTs)), all the pathways except SIRT were altered in a manner consistent with increased lifespan. However, the expression levels of SIRT4 and SIRT7 were decreased with increasing levels of CR. Changes consistent with altered fuel utilization under CR may reduce reactive oxygen species production, which was paralleled by reduced protection. Downregulated major urinary protein (MUP) transcription suggested reduced reproductive investment. Graded CR had a positive effect on autophagy and xenobiotic metabolism, and was protective with respect to cancer signaling. CR had no significant effect on fibroblast growth factor-21 (FGF21) transcription but affected transcription in the hydrogen sulfide production pathway. Responses to CR were consistent with several different hypotheses, and the benefits of CR on lifespan likely reflect the combined impact on multiple aging related processes.

## INTRODUCTION

Aging is accompanied by many metabolic changes and elevated risks of metabolic, cardiovascular, neuro-degenerative and other non-communicable diseases. Obesity, insulin resistance, inflammation and hyper-tension are predisposing conditions that increase in prevalence during aging and contribute to the disease state known as the metabolic syndrome of aging [1]. In 1935, evidence emerged that mammalian longevity could be increased by restricting food intake [2]. Nowadays, it is well established that restricting the amount of calories contributes to an increased lifespan and healthspan in many species [3–8], including non-human primates [9]. CR also delays the onset of diseases related to the metabolic syndrome of aging, such as atherosclerosis, type 2 diabetes mellitus and cardiovascular diseases [10–12]. The mechanism(s) by which CR mediates its beneficial effects on aging are yet to be fully comprehended and are likely a result of changes simultaneously in many tissues and pathways. Mathematical models applied to mortality rates have been used to understand if CR postpones or slows the aging process [13]. Here, we analyzed the liver trans-criptome as a part of a systems level description of graded CR responses [14–22]. From a clinical perspective the liver is well protected against aging relative to the other organs, but changes still occur in hepatic structure and function, such as declining liver regeneration, decreasing drug clearance and increasing bile cholesterol production [23]. Interestingly only four weeks of CR was able to reverse the majority of the aging-associated changes observed in murine liver [24]. Due to its central role in energy metabolism and glucose homeostasis, the liver is of great interest for genome-wide analysis to understand whole-body aging.

Different theories of aging have been proposed and over 25 years ago it was estimated that there were already more than 300 aging theories [25]. Many of these old theories have laid the ground for the progress that has been made in the current aging research field. Explanations of the impact of CR on longevity have focused on a limited set of hypotheses derived from these different theories. These hypothetical impacts of different systems in the longevity effects of CR may converge on common pathways and are hence not mutually exclusive.

Lifelong 40% CR alters hepatic fat metabolism by reducing lipogenesis, and increasing lipolysis and ketogenesis [26], while during aging there is a shift towards lipogenesis [27]. This alteration in lipid metabolism is believed to contribute to the CR-observed increase in lifespan [28,29]. During short-term CR, β-oxidation is increased and triglyceride synthesis inhibited, which leads to an improvement of liver function [30]. The disposable soma theory of aging argues that organisms reallocate energy sources to maintain the soma, at the cost of investment in reproduction [31,32]. In addition, CR also induces a shift to β–oxidation of fatty acids, which produces flavin adenine dinucleotide (FADH). Unlike carbo-hydrate utilization (nicotinamide adenine dinucleotide (NADH) production) FADH bypasses complex I in the electron transport chain, which is a primary site for reactive oxygen species (ROS) production [33]. The free radical theory of aging argues that accumulation of damage due to ROS leads to a gradual decline in cellular function [34]. CR could be protective against oxidative stress damage achieved by a decrease in the rate at which ROS are generated, an increase in the rate at which ROS are detoxified and/or an up-regulation of degradation and repair processes (reviewed in [4]).

Several major pathways have also been implicated to impart the beneficial effects associated with CR. These are reduced insulin/insulin like growth factor (IGF-1) signaling [15,35,36], reduced mechanistic target of rapamycin (mTOR) signaling [37], reduced nuclear factor-kappa beta (NF-κB) signaling [38] and increased sirtuin signaling [39]. Downregulation of the evolutionary conserved insulin/IGF-1 signaling pathway is associated with increase in lifespan in worms, flies and rodents [40–44]. The insulin/IGF-1 signaling pathway regulates its downstream effects via regulation of phosphoinositide-3-kinase (PI3K) and protein kinase B (AKT). In addition, the insulin/IGF-1 pathway has downstream effects on the mTOR complex 1 via AKT/Tuberous sclerosis 1 (TSC1). In response to nutrients and hormones, the mTOR signaling pathway can regulate protein synthesis, cellular growth and metabolism (reviewed in [45]). Inhibition of this pathway can increase lifespan in model organisms and inhibition is protective against aging-associated diseases (reviewed in [37]). NF-ĸB signaling is also associated with aging, mainly as its activation is linked with inflammation and known lifespan regulators insulin/IGF-1 and mTOR (reviewed in [38]). Increased sirtuin signaling has been found to increase lifespan in numerous species [46–49] and can also interact with the insulin signaling pathway [50]. Sirtuin signaling is linked to autophagy, and the increased autophagy under CR may contribute to the increase in lifespan [51,52]. Lastly the beneficial effects of CR on cancer are well known and its anti-cancer effect may also be involved in extending lifespan (reviewed in [53]).

Many of these pathways have common signaling cascades and therefore might coincide in their effects. Hence, we concentrated on the main mechanisms proposed for the anti-aging effects of CR: modulated insulin/IGF-1, mTOR, NF-ĸB and Sirtuin signaling, reduced oxidative stress, the disposable soma theory, anti-cancer mechanisms and increased autophagy. In addition, the role of fibroblast growth factor-21 (FGF21), a hormone secreted by liver during fasting, has gained recent prominence in the aging field [54] and hydrogen sulfide (H_2_S) was also recently proposed to play a key role in aging [55]. We therefore included these in our analysis. Lastly, we also included xenobiotic metabolism as a separate mechanism from oxidative stress as suggested by studies in worms [56]. In most rats and mice increasing levels of CR in both sexes are linearly related to the increase in lifespan [3,4,57]. Hence the use of graded levels of CR as a research tool has gained much prominence in recent years [58–60]. C57BL/6 mice that are both known responders to CR and a well-studied strain, were the subject of the mouse genome project and are therefore ideal to study the graded effects of CR. Linear changes in gene expression with the level of restriction may likely be key components driving the longevity response. We therefore used a correlation approach across different levels of CR to investigate if the hypothesized mechanisms mediating the CR effect responded in a linear fashion when mice were exposed to graded levels of CR.

## RESULTS

### General correlation approach mapped onto pathways from IPA and custom built pathways

We used 6 levels of restriction (5 month old male mice, restricted for 3 months until 8 months old): 24 hours *ad libitum* (AL) feeding, 12 hours AL feeding (time restricted feeding), 10% CR, 20% CR, 30% CR and 40% CR which will be referred to as 24AL, 12AL, 10CR, 20CR, 30CR and 40CR respectively. The gene expression levels were correlated with the increase in restriction. If a pathway mediated the effect of CR on lifespan, we expected gene expression in this pathway to correlate with the increase in CR level. We constructed several pathways that represent the different mechanisms related to aging. The pathways were custom built based on expert knowledge and curated databases using Ingenuity Pathway Analysis (IPA) software. The pathways were colored based on a cut-off of an absolute correlation coefficient higher than 0.3. Regulation of the downstream pathways from the aging mechanisms are identified by pathway analysis in IPA. In addition to the p-value for individual pathways we also calculated a z-value reflecting up or down regulation of the entire pathway (for more details see methods).

### The effect of graded CR on the insulin/IGF-1 signaling pathway

**Figure 1:**
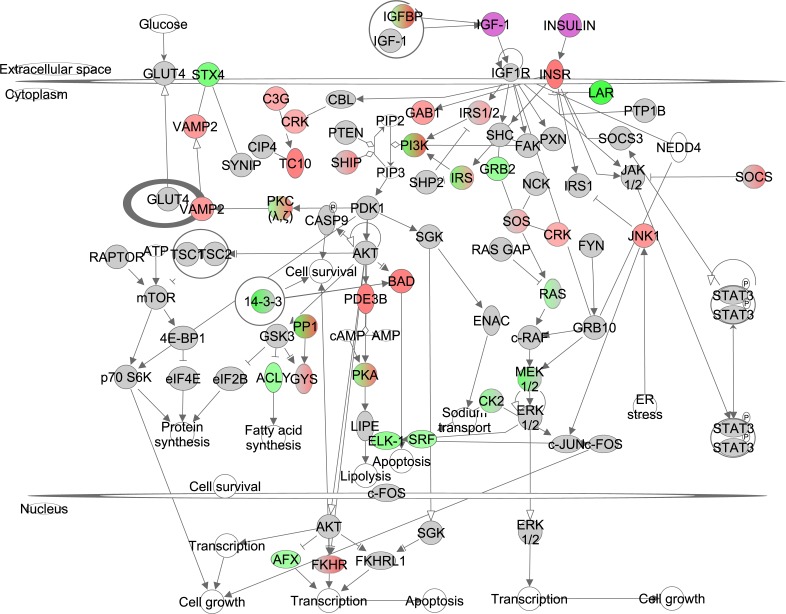
The insulin like growth factor (IGF-1)/insulin pathway created in the Ingenuity Pathway Analysis (IPA, www.qiagen.com/ingenuity) program The normalized counts for each gene were correlated with the increase in calorie restriction (CR) level by Pearson correlation method. The pathway is colored based on a cut-off of an absolute correlation coefficient higher than 0.3.. Red indicates a positive correlation with increasing CR level while green indicates a negative correlation. Circulating levels of insulin and IGF-1 were significantly reduced in these CR mice [15] and this is indicated by a purple color.

Insulin activates the insulin receptor (INSR) and downstream the insulin receptor substrate (IRS) family. Phosphorylated IRS proteins expose binding sites for signaling partners including PI3K which ultimately activates AKT. IGF-1 binds to its receptor (IGF1R) and induces a signaling cascade also via PI3K/AKT. In addition, AKT regulates mTOR via TSC1/TSC2. The INSR correlated positively with the increase in CR, probably reflecting the lowered levels of circulating insulin [15], while members of IRS family, PI3K, ATP citrate lyase (*Acly*) correlated negatively. Further downstream several target genes correlated negatively with the extent of CR (Figure 1, Table S1). However, other components in this pathway correlated positively (Figure 1, Table S1). The PI3K/AKT (z-score: -3.138, p-value: 0.019) and IGF-1 signaling (z-score: -1.091, p-value: 0.002) pathways were both significantly downregulated and hence the insulin/IGF-1 signaling appeared to be reduced in direct proportion to the increasing severity of CR. Significantly altered pathways downstream from Insulin/IGF-1 included changes in gluconeogenesis (z-score: 0.370, p-value: <0.001), lipolysis (z-score: NA, p-value: 0.046), and protein synthesis (z-score: -2.115, p-value: <0.001).

We previously measured urinary major urinary proteins (MUPs), food anticipatory activity (FAA) and basal metabolic rate (BMR) in the same animals [15,18,21]. We assessed whether changes in urinary MUPs, FAA and BMR were associated with gene expression levels of genes involved in the insulin/IGF-1 signaling pathway. In total, gene expression levels of 13 genes involved in insulin/IGF-1 signaling correlated significantly with FAA, 7 with urinary MUPs and 10 with BMR (Table 1).

**Table 1:** Expression levels of gene involved in insulin/IGF-1 signaling pathway correlated with urinary major urinary proteins (MUPs), food anticipatory activity (FAA) and basal metabolic rate (BMR)

	MUPs		FAA		BMR	
	r	p-value	r	p-value	r	p-value
*Acly*	0.425	0.019				
*Bad*			0.419	0.042	−0.350	0.031
*Casp9*			−0.488	0.016		
*Eif4ebp1*			−0.486	0.016		
*Elk1*			−0.428	0.037	0.407	0.011
*Foxo4*	0.431	0.018				
*Fyn*			−0.442	0.031	0.457	0.004
*Grb2*					0.423	0.008
*Insr*			0.408	0.048	−0.461	0.004
*Irs1*	0.419	0.021				
*Lipe*			−0.410	0.047		
*Mapk8*			0.495	0.014	−0.334	0.041
*Nck1*	0.495	0.005				
*Pde3b*			0.530	0.008	−0.443	0.005
*Ptprf*	0.440	0.015	−0.648	0.001	0.549	<0.001
*Rapgef1*	−0.378	0.039				
*Rhoq*			0.490	0.015	−0.322	0.049
*Socs3*			−0.441	0.031		
*Srf*			−0.405	0.049		
*Stx4a*	0.680	<0.001			0.481	0.002

Normalized gene counts for each individual was correlated with their corresponding physiological and behavioral data.

### The effect of graded CR on the mTOR pathway

**Figure 2:**
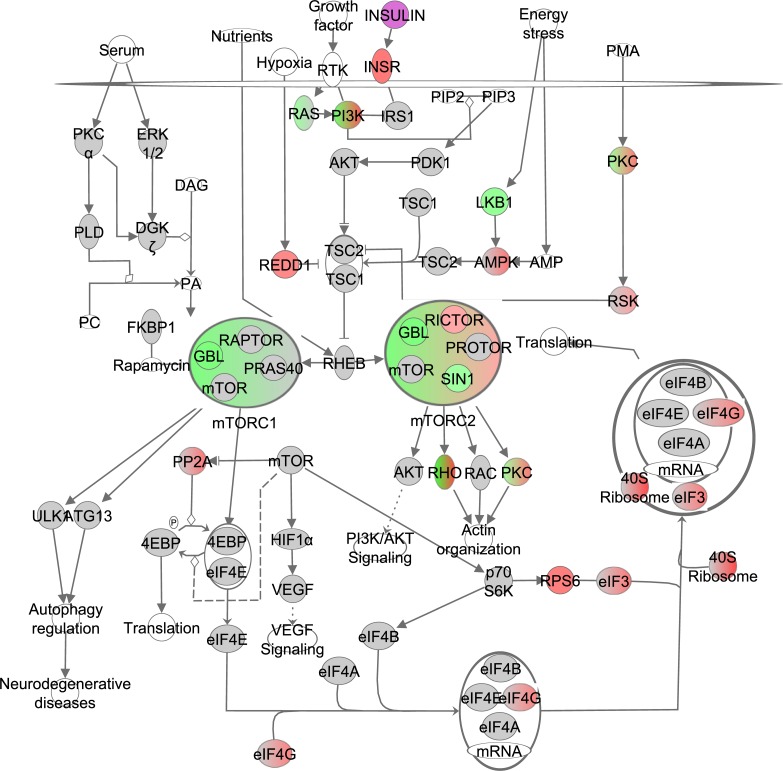
The mechanistic target of rapamycin (mTOR) signaling pathway obtained from the Ingenuity Pathway Analysis (IPA, www.qiagen.com/ingenuity) program The normalized counts for each gene were correlated with the increase in calorie restriction (CR) level by Pearson correlation method. The pathway is colored based on a cut-off of an absolute correlation coefficient higher than 0.3. Red indicates a positive correlation with increasing CR level while green indicates a negative correlation. Circulating levels of insulin were significantly reduced in these CR mice [15] and this is indicated by a purple color.

mTOR exists in two complexes: mTORC1 and mTORC2. Complex one contains the regulatory associated protein of MTOR, complex 1 (RAPTOR or *Rptor*), MTOR associated protein, LST8 homolog (GBL or *Mlst8*) and AKT1 substrate 1 (PRAS40 or *Akt1s1*) while complex two contains GBL, RPTOR independent companion of MTOR, complex 2 (RICTOR), the PROTOR group and mitogen-activated protein kinase associated protein 1 (SIN1 or *Mapkap1*). Both mTOR complexes are stimulated by Ras homolog enriched in brain (RHEB) which is downstream from insulin, growth factor and nutrient signaling pathways. RHEB is negatively regulated by TSC1/2. Gene expression of the components of both mTORC1 and mTORC2 were negatively correlated with increasing CR level, indicating significantly reduced signaling of mTOR under increasing CR (Figure 2, Table S2). Based on the correlation coefficient of the genes involved in mTORC1 and mTORC2, these 2 complexes were predicted in IPA to be negatively correlated with the increase in CR. However, no differences were observed between mTORC1 and mTORC2 in the extent of downregulation. The overall mTOR signaling pathway was indeed downregulated (z-score: -0.18, p-value: <0.001). Genes clustered together by IPA as the RHO group downstream from mTORC2 also correlated negatively with the increase in CR. RICTOR on the other hand correlated positively with the increase in CR. Expression levels of ribosomal protein S6 kinase beta-1 (*S6k1*) did not correlate with the increase in CR. Graded CR significantly altered several pathways downstream from mTOR including: autophagy (z-score: 0.833, p-value: <0.001), lipid metabolism (z-score: NA, p-value: <0.001), angiogenesis (z-score: 0.829, p-value: <0.001), cell proliferation (z-score -0.357, p-value: <0.001) and protein synthesis (z-score: -2.115, p-value: <0.001).

### The effects of graded CR on NF-ĸB signaling

**Figure 3:**
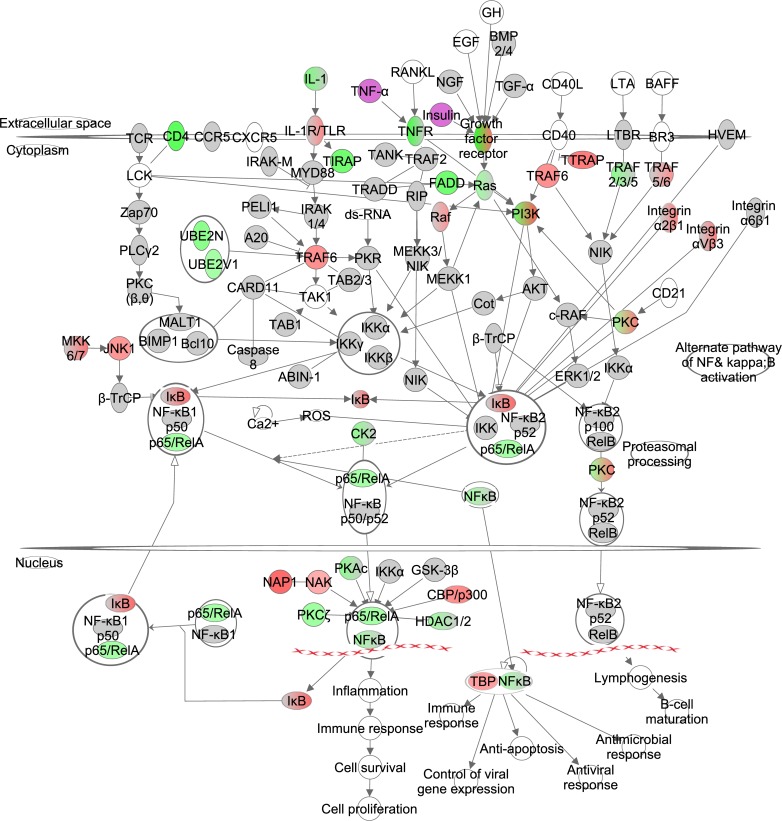
The nuclear factor kappa B (NF-ĸB) signaling pathway constructed in the Ingenuity Pathway Analysis (IPA, www.qiagen.com/ingenuity) program The normalized counts for each gene were correlated with the increase in calorie restriction (CR) level by Pearson correlation method. The pathway is colored based on a cut-off of an absolute correlation coefficient higher than 0.3. Red indicates a positive correlation with increasing CR level while green indicates a negative correlation. Circulating levels of insulin and TNF-α were significantly reduced in these mice [15] and this is indicated by a purple color.

The IKK complex (inhibitor of kappaB kinase beta (IKKβ) and conserved helix-loop-helix ubiquitous kinase (IKKα)) phosphorylates IκB proteins, and is activated by cytokines, growth factors and antigen receptors. Phosphorylation of IκB leads to downstream inactivation of the NF-κB/Rel complex via ubiquitina-tion and proteasomal degradation. NF-κB/Rel proteins include NF-κB2 p52/p100, NF-κB1 p50, p65/v-rel reticuloendotheliosis viral oncogene homolog A (avian) (RelA), and avian reticuloendotheliosis viral (v- rel) oncogene related B (RelB) and transcribes target genes in the nucleus. In addition, IKKα can phosphorylate NF-κB2 p100 via an alternative pathway. This leads to downstream activation of RelB and induces target gene transcription in the nucleus. IĸB was positively correlated with the increase in CR level and the NF-ĸB/RelB complexes were negatively correlated, indicating the increasing levels of CR progressively reduced signaling of this pathway (Figure 3, Table S3). NF-ĸB induced significant changes in the inflammation and immune response, cell proliferation (z-score -0.357, p-value: <0.001) and lymphogenesis (z-score: -0.539, p-value: <0.001)

We previously measured circulating hormone levels of the same mice [15] and assessed whether expression levels of genes involved in the NF-κB signaling path-way were significantly associated with these hormones. In total 4 genes correlated with leptin, 5 with insulin, 1 with interleukin 6 (IL6), 7 with tumor necrosis factor-alpha (TNF-α) and 11 with IGF-1 (Table 2).

**Table 2:** Expression levels of gene involved in NF-κB signaling pathway correlated with levels of circulating hormones

	leptin		insulin		IL6		TNF-α		IGF-1	
	r	p	r	p	r	p	r	p	r	p
*Azi2*			−0.508	0.003			−0.376	0.031	−0.517	0.002
*Card11*							0.391	0.024		
*Cd4*			0.417	0.016			0.429	0.013		
*Chuk*					−0.400	0.038				
*Fadd*							0.577	<0.001		
*Gsk3b*	−0.371	0.034	−0.427	0.013					−0.568	0.001
*Malt1*									−0.347	0.048
*Mapk8*									−0.534	0.001
*Peli1*			−0.361	0.039						
*Prkcz*									0.513	0.002
*Rela*							0.360	0.039		
*Ripk1*	−0.416	0.016							−0.433	0.012
*Tab1*									−0.367	0.036
*Tbk1*									−0.368	0.035
*Tbp*	−0.374	0.032							−0.386	0.027
*Tdp2*									−0.455	0.008
*Tirap*	0.362	0.038	0.477	0.005						
*Tradd*							0.435	0.011		
*Traf6*									−0.492	0.004
*Ube2n*							0.544	0.001		

Normalized gene counts for each individual was correlated with their corresponding physiological and behavioral data.

### The effects of graded CR on sirtuin signaling

**Figure 4:**
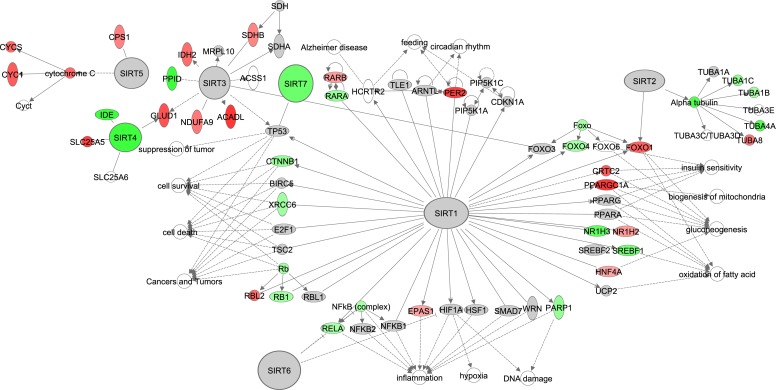
The sirtuin signaling pathway constructed in the Ingenuity Pathway Analysis (IPA, www.qiagen.com/ingenuity) program The normalized counts for each gene were correlated with the increase in calorie restriction (CR) level by Pearson correlation method. The pathway is colored based on a cut-off of an absolute correlation coefficient higher than 0.3. Red indicates a positive correlation with increasing CR level while green indicates a negative correlation.

The sirtuin signaling pathway has shared signaling cascades with mTOR signaling, NF-ĸB and insulin signaling, and gene expression of some members of these pathways correlated negatively with the increase of CR (Figure 2-3, Table S2-S3). There are seven sirtuins in mammals (SIRT1-7) which are involved in various biological functions. We constructed this pathway based on the Sirtuins at a glance publication by Nakagawa and Guarente (2011) [61]. Of these 7 SIRTs only *Sirt4* and *Sirt7* correlated negatively with the increase in CR (Figure 4, Table S4). Target genes of *Sirt4*, insulin degrading enzyme (IDE) and solute carrier family 25 (mitochondrial carrier, adenine nucleotide translocator), member 5 (SLC254A), correlated negatively and positively respectively with graded CR.

### The effects of graded CR on oxidative stress signaling

**Figure 5:**
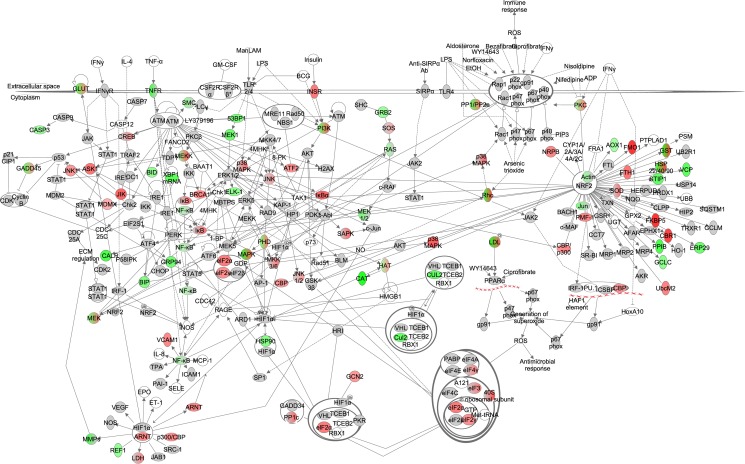
The oxidative stress signaling pathway constructed in the Ingenuity Pathway Analysis (IPA, www.qiagen.com/ingenuity) program The normalized counts for each gene were correlated with the increase in calorie restriction (CR) level by Pearson correlation method. The pathway is colored based on a cut-off of an absolute correlation coefficient higher than 0.3. Red indicates a positive correlation with increasing CR level while green indicates a negative correlation.

It has been hypothesized that CR may modulate oxidative stress by decreasing the rate at which ROS are generated, increasing the rate at which ROS are detoxified and upregulating degradation and repair processes (reviewed in [4]). This pathway includes processes such as nitric oxide (NO) production, nuclear factor erythroid derived 2 (NRF2) signaling, hypoxia-inducible factor 1-alpha (HIF1-α) signaling, eukaryotic initiation factor 2 (eIF2) signaling, inducible nitric oxide synthase (iNOS) signaling and NF-ĸB signaling. Interleukins, TNF-α and insulin have been suggested to induce expression in this pathway. Under graded CR, gene expression in this pathway indicated that genes involved in the eIF2 signaling (z-score: 4.718, p-value: <0.001) were positively correlated with the extent of restriction while NRF2 (z-score: NA, p-value: 0.001) correlated negatively (Figure 5, Table S5). In addition, the production of ROS was predicted to be decreased (z-score: -0.305, p-value: <0.001).

We previously measured activity levels of antioxidants in livers of the same individual mice [15] and we assessed whether their expression levels correlated with the corresponding enzyme activity levels. The expression levels of the gene catalase (*Cat*) correlated positively with the measured actively levels of this antioxidant (r: 0.378, p-value: 0.019). Gene members of the superoxide dismutase family correlated negatively with activity levels of superoxide dismutase (*Sod1* r: -0.325 p-value: 0.046; *Sod2* r: -0.335 p-value: 0.039). None of the gene members of the glutathione peroxidase (GPx) family correlated significantly with the anti-oxidant activity level of GPx. In total, 10 genes involved in oxidative stress correlated with SOD activity levels, 29 with catalase and 9 with GPx (Table 3). Gene expression levels were also correlated with circulating hormone levels of the same mice [15] (Table 4). In total 30 genes correlated significantly with leptin, 33 with insulin, 23 with TNF-α, 10 with IL6, 34 with IGF-1 and 36 with resistin.

**Table 3:** Expression levels of gene involved in oxidative stress signaling correlated with activity levels of antioxidants

	SOD		catalase		GPx	
	r	p-value	r	p-value	r	p-value
*Apex1*			0.385	0.017	0.339	0.037
*Atf4*	0.343	0.035			0.432	0.007
*Brca1*	−0.329	0.044				
*Calr*			0.333	0.041		
*Casp3*			0.323	0.048	0.539	<0.001
*Cat*			0.380	0.019		
*Cbr1*	−0.345	0.034	−0.382	0.018		
*Crebbp*			−0.340	0.037		
*Cul2*			0.377	0.020		
*Eif2a*	−0.423	0.008				
*Eif2s1*			0.324	0.047		
*Eif4e*			0.382	0.018		
*Fkbp5*			−0.457	0.004		
*Fmo1*			−0.472	0.003		
*Fth1*	−0.445	0.005	−0.387	0.016		
*Hsp90aa1*			0.415	0.010		
*Hsp90b1*	0.344	0.034	0.359	0.027	0.377	0.020
*Hspa5*			0.388	0.016		
*Insr*			−0.329	0.044		
*Map2k1*	0.371	0.022	0.379	0.019		
*Map2k5*	−0.321	0.049				
*Mapk14*	−0.370	0.022	−0.437	0.006		
*Ncf4*			0.392	0.015		
*Nfkbia*			−0.419	0.009		
*Ppib*			0.334	0.040		
*Prkcb*					0.351	0.031
*Scarb1*			−0.323	0.048		
*Serpine1*						
*Shc1*					0.410	0.011
*Stip1*			0.438	0.006		
*Taok3*			−0.451	0.004		
*Tdp1*					0.412	0.010
*Trp53bp1*			0.371	0.022		
*Ube2e3*			−0.427	0.008		
*Usp14*			0.340	0.037		
*Vcp*			0.448	0.005	0.330	0.043

Normalized gene counts for each individual was correlated with their corresponding physiological and behavioral data.

**Table 4:** Expression levels of gene involved in oxidative stress signaling correlated with levels of circulating hormones

	leptin		insulin		TNF-α		IL6		IGF-1		resistin	
	r	p	r	p	r	p	r	p	r	p	r	p
*Abcc1*											−0.422	0.028
*Abcc2*	−0.471	0.006	−0.418	0.016					−0.563	0.001		
*Abl1*	−0.365	0.037										
*Aox1*					0.354	0.044						
*Apex1*					0.365	0.037						
*Arnt*	−0.413	0.017							−0.556	0.001		
*Atf2*			−0.464	0.007					−0.544	0.001		
*Bach1*			−0.477	0.005								
*Calr*	0.374	0.032	0.621	<0.001	0.517	0.002			0.545	0.001		
*Casp9*							0.429	0.026				
*Cat*	0.523	0.002	0.428	0.013	0.509	0.002	−0.497	0.008	0.625	<0.001		
*Cbr1*	−0.570	0.001	−0.514	0.002	−0.383	0.028			−0.654	<0.001		
*Cbx5*			−0.392	0.024								
*Cdc25a*	−0.371	0.033	−0.373	0.033					−0.451	0.008		
*Cdc34*											−0.382	0.049
*Cdkn1a*											−0.403	0.037
*Clpp*							0.554	0.003			−0.444	0.020
*Cops5*											−0.410	0.034
*Crebbp*	−0.525	0.002	−0.419	0.015					−0.579	<0.001		
*Csf2ra*											−0.411	0.033
*Csf2rb2*											−0.424	0.028
*Cul2*			0.416	0.016	0.465	0.006					−0.453	0.018
*Cybb*											−0.437	0.023
*Eif1a*											−0.382	0.049
*Eif2a*	−0.396	0.023	−0.504	0.003					−0.433	0.012		
*Eif2ak4*									−0.478	0.005		
*Eif2s1*											−0.404	0.037
*Eif2s3x*									−0.553	0.001		
*Eif4e*											−0.425	0.027
*Enc1*	−0.404	0.020							−0.509	0.002		
*Erp29*			0.420	0.015	0.458	0.007			0.361	0.039	−0.495	0.009
*Fkbp5*	−0.581	<0.001	−0.487	0.004	−0.597	<0.001			−0.515	0.002		
*Fmo1*	−0.605	<0.001	−0.548	0.001	−0.647	<0.001			−0.569	0.001		
*Fth1*			−0.415	0.016			0.427	0.027	−0.458	0.007		
*Gclc*					0.354	0.043						
*Gsk3b*	−0.371	0.034	−0.427	0.013					−0.568	0.001		
*Gsr*											−0.447	0.019
*H2afx*											−0.461	0.015
*Herpud1*	−0.408	0.018										
*Hif1a*							−0.401	0.038				
*Hsp90aa1*											−0.501	0.008
*Hsp90b1*			0.522	0.002	0.488	0.004						
*Hspa5*											−0.431	0.025
*Icam1*											−0.397	0.040
*Insr*	−0.466	0.006	−0.546	0.001	−0.407	0.019			−0.481	0.005		
*Maf*	−0.344	0.050	−0.478	0.005	−0.479	0.005						
*Map2k1*			0.427	0.013	0.354	0.043			0.373	0.032	−0.444	0.020
*Map3k1*											−0.399	0.039
*Map3k5*	−0.485	0.004	−0.644	<0.001	−0.557	0.001			−0.394	0.023		
*Mapk14*	−0.560	0.001	−0.496	0.003	−0.380	0.029			−0.462	0.007		
*Mapk8*									−0.534	0.001		
*Mdm2*									−0.457	0.008		
*Mdm4*			−0.353	0.044					−0.578	<0.001		
*Ncf2*											−0.417	0.030
*Ncf4*											−0.460	0.016
*Ncoa1*									−0.554	0.001		
*Nfkbia*	−0.453	0.008	−0.507	0.003	−0.390	0.025			−0.478	0.005		
*Pdpk1*			−0.390	0.025					−0.525	0.002		
*Plat*											−0.453	0.018
*Pmf1*							0.388	0.046				
*Ppara*			−0.392	0.024							0.508	0.007
*Ppib*	0.427	0.013	0.650	<0.001	0.492	0.004			0.530	0.002		
*Ppp1r15a*	−0.356	0.042										
*Prdx1*	0.360	0.039										
*Scarb1*	−0.383	0.028					0.400	0.039				
*Serpine1*							0.460	0.016			−0.452	0.018
*Shc1*											−0.423	0.028
*Sirpa*											−0.433	0.024
*Sqstm1*											−0.441	0.021
*Stip1*			0.357	0.041							−0.411	0.033
*Taok3*	−0.456	0.008	−0.507	0.003					−0.473	0.005		
*Tlr4*											−0.439	0.022
*Trim28*	−0.415	0.016										
*Trp53*											−0.422	0.028
*Trp53bp1*											−0.520	0.005
*Txn1*	0.406	0.019			0.403	0.020					−0.454	0.017
*Txnrd1*											−0.458	0.016
*Ube2k*									−0.430	0.013		
*Vcam1*	−0.506	0.003	−0.422	0.015					−0.437	0.011		
*Vcp*			0.509	0.003	0.499	0.003						
*Xbp1*	0.438	0.011			0.370	0.034			0.485	0.004		

Normalized gene counts for each individual was correlated with their corresponding physiological and behavioral data.

### The effects of graded CR on reproduction pathways

**Figure 6:**
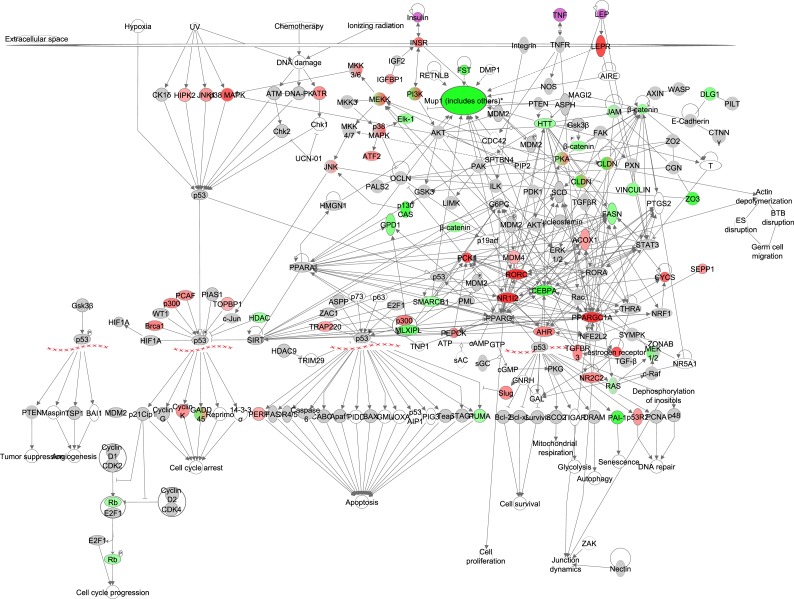
The reproduction pathway constructed in the Ingenuity Pathway Analysis (IPA, www.qiagen.com/ingenuity) program The normalized counts for each gene were correlated with the increase in calorie restriction (CR) level by Pearson correlation method. The pathway is colored based on a cut-off of an absolute correlation coefficient higher than 0.3. Red indicates a positive correlation with increasing CR level while green indicates a negative correlation. Circulating levels of insulin, TNF-α and leptin were significantly reduced in these mice [15] and this is indicated by a purple color.

Major urinary proteins (MUPs) are used by male mice in scent marking to attract females and their synthesis is a major cost of reproduction [31,62,63]. The p53 signaling pathway has also been implicated to play are role in reproduction [64,65]. However downstream genes of p53 signaling did not correlate significantly with CR. Gene expression of the different MUPs all correlated negatively with the extent in restriction (Figure 6, Table S6). Expression levels of MUPs genes also correlated strongly in a positive manner with several circulating hormones measured in the same mice (Table 5). Other gene products involved in reproduction such as PPARGC1A, RAR-related orphan receptor gamma (RORC), phosphoenolpyruvate carbo-xykinase 1, cytosolic (PCK1) and nuclear receptor subfamily 1, group I, member 2 (NR1I2) correlated strongly in a positive manner with the increase in CR (Table S6).

**Table 5:** Gene expression levels of major urinary proteins (MUPs) correlated with circulating hormone levels

	Insulin		IGF-1		TNF-α		Leptin	
	r	p-value	r	p-value	r	p-value	r	p-value
*Mup1*	0.345	0.049	0.414	0.017	0.292	0.099	0.475	0.005
*Mup3*	0.475	0.005	0.654	<0.001	0.443	0.010	0.732	<0.001
*Mup4*	0.279	0.116	0.565	0.001	0.418	0.016	0.579	<0.001
*Mup5*	0.319	0.070	0.595	<0.001	0.306	0.083	0.686	<0.001
*Mup6*	0.338	0.055	0.521	0.002	0.343	0.051	0.645	<0.001
*Mup7*	0.456	0.008	0.497	0.003	0.434	0.012	0.582	<0.001
*Mup8*	0.486	0.004	0.431	0.012	0.452	0.008	0.618	<0.001
*Mup9*	0.528	0.002	0.603	<0.001	0.450	0.009	0.592	<0.001
*Mup10*	0.534	0.001	0.661	<0.001	0.464	0.006	0.739	<0.001
*Mup11*	0.491	0.004	0.553	0.001	0.375	0.031	0.556	0.001
*Mup12*	0.320	0.070	0.397	0.022	0.326	0.064	0.466	0.006
*Mup14*	0.553	0.001	0.486	0.004	0.439	0.011	0.571	0.001
*Mup16*	0.495	0.003	0.489	0.004	0.457	0.007	0.626	<0.001
*Mup17*	0.440	0.010	0.516	0.002	0.382	0.028	0.636	<0.001
*Mup20*	0.581	<0.001	0.640	<0.001	0.529	0.002	0.714	<0.001
*Mup21*	0.447	0.009	0.558	0.001	0.368	0.035	0.669	<0.001

Normalized gene counts for each individual was correlated with their corresponding physiological and behavioral data.

### The effects of graded CR on cancer signaling pathways

**Figure 7:**
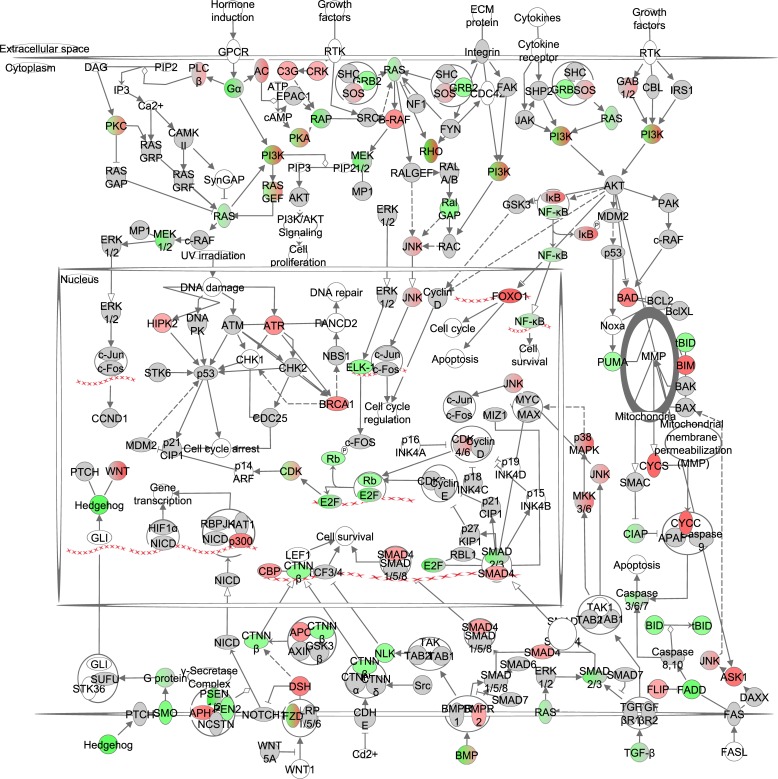
The cancer signaling pathway obtained from the Ingenuity Pathway Analysis (IPA, www.qiagen.com/ingenuity) program The normalized counts for each gene were correlated with the increase in calorie restriction (CR) level by Pearson correlation method. The pathway is colored based on a cut-off of an absolute correlation coefficient higher than 0.3. Red indicates a positive correlation with increasing CR level while green indicates a negative correlation. The string of Xs represent DNA and indicate a transcriptional effect of a gene.

A number of pathways are involved in cancer signaling, and show overlap with the oxidative stress signaling pathway (e.g. HIF1-α, NO, NRF2, iNOS). Expression of genes downstream from p53 did not correlate with the increase in CR but NF-ĸB correlated negatively with the increase in CR. Hedgehog, TGF-β and Catenin beta 1 (CTNNβ) signaling all showed negative correlations to the extent of increasing CR (Figure 7, Table S7). The tumor suppressor genes of the SMAD family also correlated positively with CR (Figure 7, Table S7). The ‘disease function’ cancer was predicted to be strongly decreased (z-score: -3.285, p-value: <0.001).

### The effects of graded CR on autophagy

**Figure 8:**
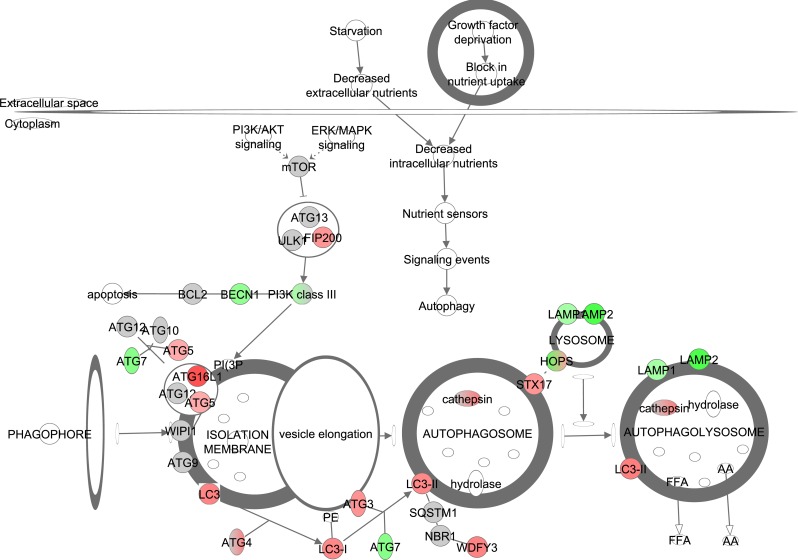
The autophagy signaling pathway obtained from the Ingenuity Pathway Analysis (IPA, www.qiagen.com/ingenuity) program The normalized counts for each gene were correlated with the increase in calorie restriction (CR) level by Pearson correlation method. The pathway is colored based on a cut-off of an absolute correlation coefficient higher than 0.3. Red indicates a positive correlation with increasing CR level while green indicates a negative correlation.

Starvation and nutrient deprivation induce autophagy. In addition, PI3K/AKT (insulin pathway) signaling activates mTOR and ERK/MAPK signaling negatively regulates mTOR, which both lead to an activation of mTOR and suppression of autophagy. Downstream from mTOR, UNC-51 like kinase 1 (ULK1) forms a large complex with autophagy related 13 (AGT13) and the scaffold protein FIP200. PI3K class III is required for the induction of autophagy. The ATG genes control autophagosome formation through ATG12/ATG5 which requires ATG7 and ATG10. This induces the lipidated form of LC3 (LC3-II) which is attached to the autophagosome membrane. FIP200 and the further downstream AGT genes correlated positively with CR (Figure 8, Table S8). Similar results were found for LC3, LC3-I and LC3-II. Hence, autophagy was significantly increased with increasing levels of CR (z-score: 0.833, p-value: <0.001). Gene expression levels of genes involved in autophagy correlated negatively with circulating levels of IGF-1 measured in the same mice (Table 6).

**Table 6:** Expression levels of genes involved in autophagy correlated with circulating hormone levels

	Insulin		IGF-1		TNF		Leptin	
	r	p-value	r	p-value	r	p-value	r	p-value
*Atg10*	−0.062	0.731	0.094	0.602	0.116	0.520	0.052	0.775
*Atg13*	0.138	0.443	−0.166	0.356	0.185	0.303	−0.044	0.809
*Atg16l1*	−0.297	0.093	−0.566	0.001	−0.148	0.412	−0.359	0.040
*Atg7*	0.043	0.811	0.422	0.014	0.033	0.854	0.180	0.317
*Lamp2*	0.298	0.092	0.656	<0.001	0.302	0.088	0.701	<0.001
*Map1lc3a*	−0.229	0.200	−0.133	0.460	−0.180	0.316	−0.131	0.469
*Map1lc3b*	−0.433	0.012	−0.166	0.355	−0.278	0.118	−0.426	0.013
*Rb1cc1*	−0.502	0.003	−0.609	<0.001	−0.221	0.216	−0.296	0.095
*Stx17*	−0.500	0.003	−0.456	0.008	−0.323	0.067	−0.335	0.057
*Wdfy3*	−0.509	0.002	−0.466	0.006	−0.429	0.013	−0.446	0.009

Normalized gene counts for each individual was correlated with their corresponding physiological and behavioral data.

### The effects of graded CR on mitochondrial biogenesis and fuel utilization

**Figure 9:**
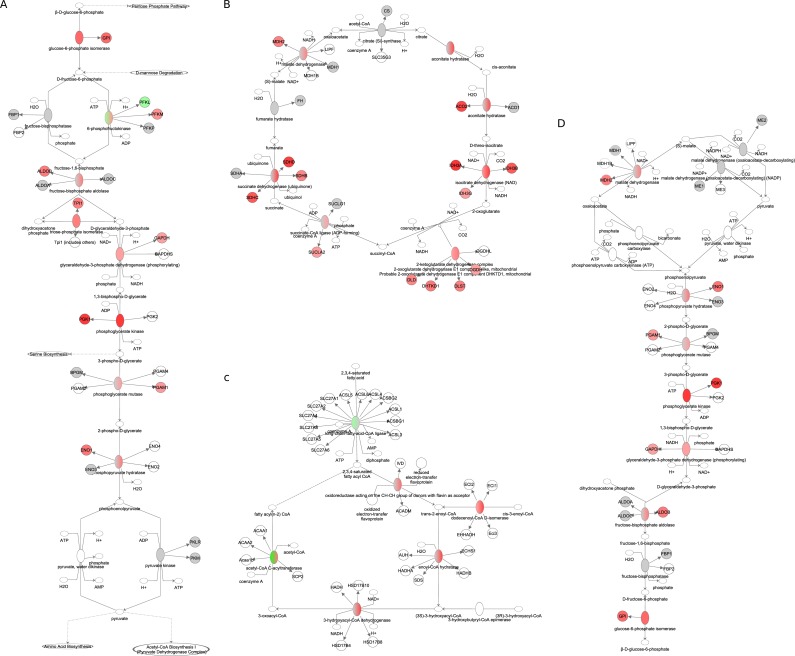
Fuel utilization pathways obtained from the Ingenuity Pathway Analysis (IPA, www.qiagen.com/ingenuity) program The normalized counts for each gene were correlated with the increase in calorie restriction (CR) level by Pearson correlation method. The pathway is colored based on a cut-off of an absolute correlation coefficient higher than 0.3. Red indicates a positive correlation with increasing CR level while green indicates a negative correlation. (**A**) glycolysis. (**B**) TCA cycle (**C**) fatty acid β-oxidation. (**D**) gluconeogenesis.

**Figure 10:**
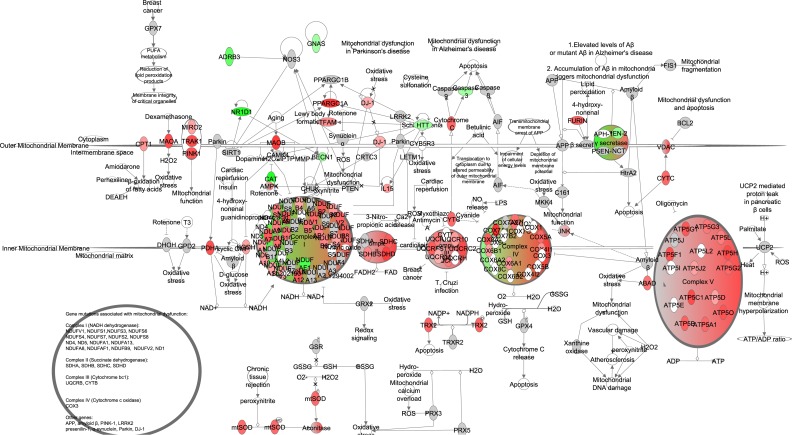
Electron transport chain and mitochondrial biogenesis constructed in the Ingenuity Pathway Analysis (IPA, www.qiagen.com/ingenuity) program The normalized counts for each gene were correlated with the increase in calorie restriction (CR) level by Pearson correlation method. The pathway is colored based on a cut-off of an absolute correlation coefficient higher than 0.3. Red indicates a positive correlation with increasing CR level while green indicates a negative correlation.

**Figure 11:**
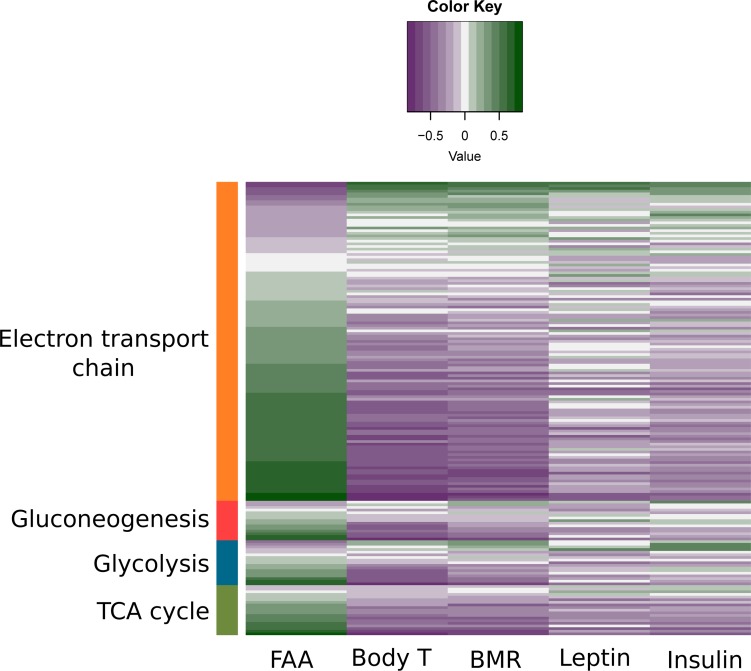
Association between physiological/behavior data and expression levels of genes involved in substrate metabolism Previously measured leptin and insulin levels, food anticipatory activity (FAA), body temperature (body T) and basal metabolic rate (BMR) [15,16,18,21] for each individual mouse were correlated with the normalized counts of genes involved in electron transport chain, gluconeogenesis, glycolysis and tricarboxylic acid (TCA) cycle. Purple indicates a negative correlation between genes and the measured physiological/behavior data while green indicates a positive correlation.

We split mitochondrial biogenesis and fuel utilization into 5 separate pathways: Glycolysis, the tricarboxylic acid cycle (TCA) cycle, fatty acid β-oxidation, and the electron transport chain (ETC) combined with mitochondrial biogenesis genes and gluconeogenesis. The genes involved in glycolysis (z-score: NA, p-value: 0.016), TCA cycle (z-score: NA, p-value: <0.001), fatty acid β-oxidation (z-score: 2.872, p-value: <0.001) and gluconeogenesis (z-score: 0.370, p-value: <0.001) all showed positive correlations with the extent of restriction (Figure 9A-D). Genes involved in the ETC were also positively correlated with the increase of CR, especially at complexes II, IV and V. Genes signaling mitochondrial dysfunction and apoptosis (z-score: -1.504, p-value <0.001) were negatively correlated with CR (Figure 10, Table S9). In addition genes involved in ETC, gluconeogenesis, glycolysis and TCA correlated in general positively with FAA and negatively with circulating levels of leptin and insulin, body tempera-ture and BMR measured in the mice (Figure 11).

### The effects of graded CR on FGF21 and the H_2_S pathway

**Figure 12:**
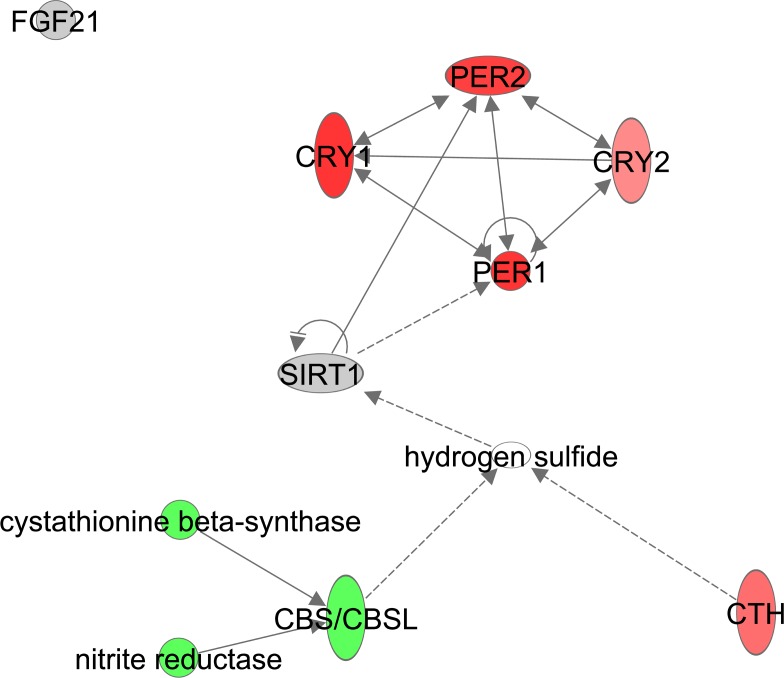
Hydrogen Sulfide production constructed in Ingenuity Pathway Analysis (IPA, www.qiagen.com/ ingenuity) program The normalized counts for each gene were correlated with the increase in calorie restriction (CR) level by Pearson correlation method. The pathway is colored based on a cut-off of an absolute correlation coefficient higher than 0.3. Red indicates a positive correlation with increasing CR level while green indicates a negative correlation.

Gene expression levels of *Fgf21* did not correlate with the extent of restriction (Table S10). H_2_S is generated *in vivo* by cystathionine-γ-lyase (CSE or *Cth*) and cystathionine-β-synthase (CBS) and was shown to have an effect on circadian rhythm genes in a NAD/NAD+ ratio and *Sirt1* dependent manner [66]. Expression levels of genes involved in circadian rhythm such as period circadian clock 1 (*Per1*), *Per2*, cryptochrome 1 (*Cry1*) and *Cry2* were significantly positively correlated with CR. The circadian pathway itself however was not significantly altered with increasing CR levels (z-score: NA, p-value: 0.080). *Cth* correlated positively with CR while *Cbs* correlated negatively (Figure 12, Table S10).

### The effects of graded CR on xenobiotic metabolism

**Figure 13:**
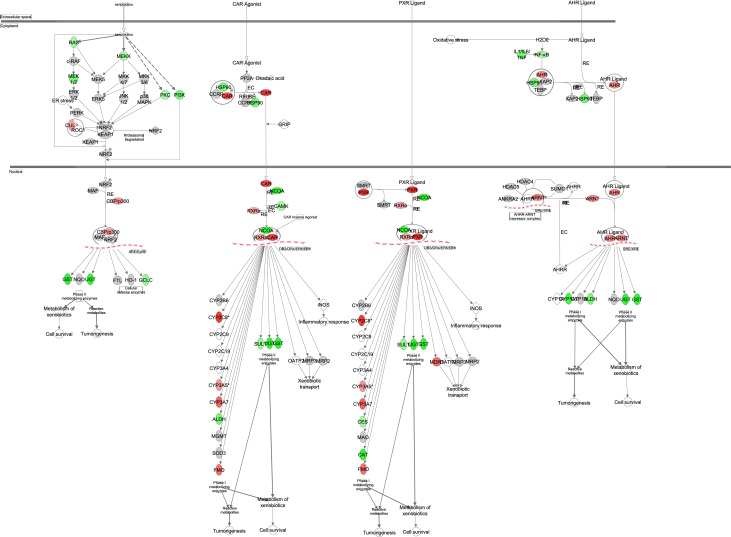
Xenobiotic metabolism obtained from the Ingenuity Pathway Analysis (IPA, www.qiagen.com/ingenuity) program The normalized counts for each gene were correlated with the increase in calorie restriction (CR) level by Pearson correlation method. The pathway is colored based on a cut-off of an absolute correlation coefficient higher than 0.3. Red indicates a positive correlation with increasing CR level while green indicates a negative correlation.

Constitutive active receptor (CAR, *Nr1I3*) is able to activate target genes by forming a complex with retinoid X receptor alpha (RXRα). Similar pregnane X receptor (PXR, *Nr1I2*) also forms a complex with RXRα. These activate transcription of enzymes involved in the xenobiotic metabolism. The xenobiotic metabolism consists of phase I, phase II and phase III enzymes. Phase I enzymes include the cytochrome P450 family (CYP), flavin containing monooxygenase (FMO) and aldehyde dehydrogenase (ALDH). Transcription levels of *Nr1I2*, *Nr1I3*, *Rxra*, *Fmo1*, *Fmo2*, *Fmo3*, *Fmo4*, *Cyp2c8*, *Cyp3a7*, *Aldh1a3*, *Aldh3a2*, *Aldh5a1*, *Aldh6a1* and *Aldh9a1* were all strongly positively associated with the increase in CR (Figure 13, Table S11). Overall metabolism of xenobiotics was predicted to be significantly upregulated (z-score: 1.183, p-value: <0.001).

### DISCUSSION

**Figure 14:**
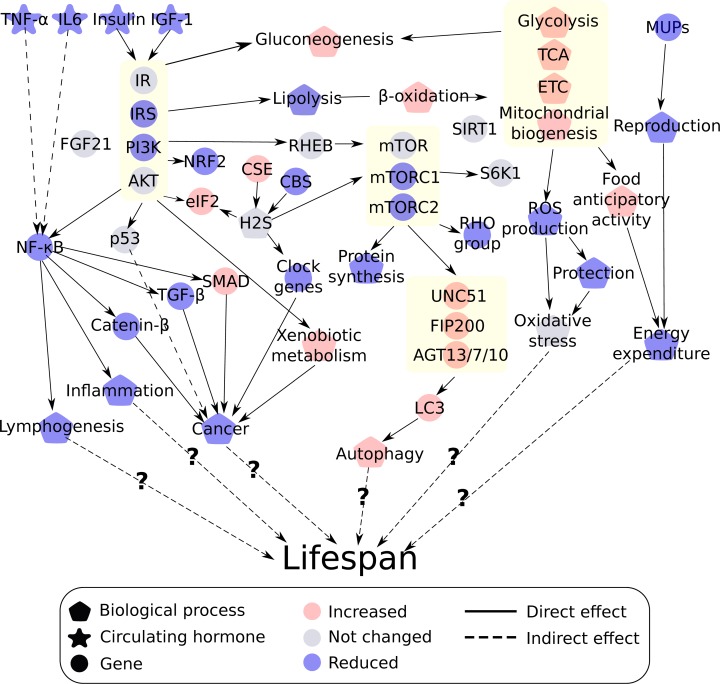
Integrated overview of the different theories of ageing affected by graded calorie restriction (CR) Red indicates a positive correlation with increasing CR while blue indicates a negative correlation. Grey indicates no linear changes with the increase in CR level. Genes or processes biologically related are grouped together indicated by a yellow box including the insulin/insulin like growth factor (IGF-1) signaling pathway, mechanistic target of rapamycin (mTOR) pathway, fuel utilization and autophagy.

In this strain of mouse graded increases in CR leads to a graded increase in lifespan [67]: hence we suggested that changes at the molecular level mimicking this effect are likely to be more important than non-linear effects or responses that are constant across all levels. Here we found that graded CR had a graded impact on several different pathways in such a way that the changes might facilitate an increase in lifespan. To summarize, we found that insulin/IGF-1, NF-ĸB, and mTOR but probably not SIRT signaling pathways were correlated with graded CR in such a way that they may mediate the effect of CR on lifespan. The observed changes in fuel utilization related genes in relation to CR level may reduce oxidative stress. Reproductive investment in the form of MUPs were negatively associated with CR. Graded CR had a positive effect on autophagy and xenobiotic metabolism and CR was protective in cancer signaling. In addition, CR had no effect on transcription rates of FGF21 but did had a positive significant effect on the H_2_S production mechanism. To aid the discussion of the results, we have included an integrated overview of the different pathways affected by graded CR (Figure 14).

### CR has transcriptional impacts on three of the four evolutionary conserved signaling pathways related to longevity

The insulin/IGF-1 signaling pathway is evolutionary conserved and its role in aging came to light from studies with *Caenorhabditis elegans* [40,68]. *C. elegans* mutants for the daf-2 gene (IGF-1 receptor), involved in insulin-like signaling, have a lifespan twice as long as wild types [41,50]. Mice lacking the insulin receptor substrate 1 (IRS1^−/−^) but not IRS2 (IRS2^−/−^) are long-lived, but strangely have lifelong insulin resistance, while being also resistant against age-sensitive markers [43,44]. The mice studied here exhibited lower circulating levels of insulin and IGF-1 under CR [15]. This was correlated with improved glucose tolerance and improved insulin sensitivity [15]. In the present paper, we found that expression levels of hepatic genes involved in the insulin/IGF-1 signaling pathway generally correlated negatively with increasing CR, including members of the IRS family: hence the pathway was inhibited and increasingly so at higher levels of restriction. Insulin signaling activates glycogen synthesis for energy storage, suppresses hepatic glucose output and initiates lipogenesis. These processes are all reduced under CR [26] and we found significant downregulation of *Acly* in this pathway which is involv ed in the synthesis of fatty acids [69,70]. In agreement with other studies that have focused on single levels of restriction [15,35,36], these results indicate insulin/IGF-1 signaling is reduced under CR in relation to the extent of restriction and hence may contribute to the graded increase in lifespan under graded CR [40–42]. However, elucidating the exact mechanism by which reduced insulin/IGF-1 regulates longevity is challenged by its complexity (reviewed in [71]). This pathway may mediate its beneficial effects via its impact on oxidative stress [72,73], the change in substrate utilization under CR [28,29], its impact in other pathways such as mTOR [74], xenobiotic detoxification mechanisms [75] and reproduction [76,77]. In addition, insulin and IGF-1 have been implicated to play a role in cancer [78].

Similar to insulin, the role of mTOR in longevity was first established in *C. elegans* where mutations in mTOR and mTORC1 component raptor (daf-15) extended longevity [79,80]. In *Drosophila melano-gaster,* mutations of mTOR and several components of the mTORC1 also increased lifespan [81,82]. This was followed by studies in mice [83,84] indicating that mTOR is an evolutionary conserved regulator of longevity. Hence, reduced mTOR signaling as a result of CR may contribute to longevity and CR-induced health benefits (reviewed in [85]). mTORC1 regulates processes related to growth and differentiation while mTORC2 plays a regulatory role in the insulin cascade [86]. Disruption of mTORC1 leads to an increased lifespan [81,82] and is believed to be the primary complex to regulate longevity. In our graded CR study, expression levels of mTORC1 and mTORC2 were both strongly negatively associated with the increase in CR. Deletion of S6K1, which is downstream from mTOR and insulin signaling, leads to an increased lifespan in female but not male mice [87]. S6K1 is a substrate of mTORC1 which is believed to be the main complex regulating longevity [88]. We found that graded CR had no effect on the expression levels of S6K1. However, activation of S6K1 is controlled via phosphorylation of at least 8 Ser/Thr residues [89]. mTOR can regulate S6K1 activation by either blocking an upstream S6K kinase or activating a phosphatase [89]. Although gene expression of S6K1 was not significantly altered in our data, its inhibition may be regulated via the down-regulated mTORC1. Hence, the results we found of the downregulated mTORC1/2 may play a role in the effect of CR on longevity. However, the effects may be minimal in these male mice as suggested by the minor effect on lifespan of knocking out the S6K1 gene in male mice [87]. Given the strongly altered pathways downstream of mTOR (autophagy, protein synthesis etc) this suggests that mTOR may have CR responsive effects independent of S6K1 in male mice. Recent work suggested that downstream mTOR signaling in male mice may be regulated via eukaryotic translation initiation factor 4E binding protein 1 (4E-BP1) [90]. Transgenic 4E-BP1 male but not female mice are protected from ageing-induced obesity [90]. Our data suggests that processes downstream from mTOR may be activated in a S6K1 independent manner or that mTOR may not play a key role in extending lifespan in male mice under CR.

A third pathway linked to aging is NF-ĸB signaling, mainly as its activation is linked to known lifespan regulators including insulin/IGF-1, mTOR and the sirtuins (reviewed in [38]). Inducible genetic inhibition of NF-ĸB in the epidermis of aged mice for two weeks resulted in a gene expression profile similar to that of young mice [91]. NF-ĸB transcription factors are also evolutionary conserved and are mainly regulators of the immune system and inflammatory responses [92]. CR is known to reduce inflammation and decreased NF-ĸB signaling has been suggested to play a key role in this response [93]. TNF-α, a major pro-inflammatory cytokine, activates NF-ĸB and is also a transcriptional target of NF-ĸB [94]. The circulating levels of TNF-α were reduced in the mice studied here [15] and expression of NF-ĸB was negatively correlated with increasing CR level but examination of the direct correlation of NF-ĸB to TNF-α showed these correlations were not significant suggesting other factors were likely more important in the suppression of NF-ĸB reported here. In addition, RelA and its down-stream genes were also downregulated. This would indeed suggest a reduced signaling of NF-ĸB under graded CR, resulting in less inflammation. In addition, NF-ĸB might also play a role in regulating energy metabolism that is independent of inflammation [95,96]. In cancer cells, RelA can regulate mito-chondrial function via binding to mitochondrial DNA and repressing gene expression affecting oxidative phospho-rylation and ATP levels [97]. RelA can activate the p53 gene and an increase in p53 levels leads to an increase in oxidative phosphorylation and a decrease in glycolysis [95]. Gene expression levels of p53 were not altered in our study but we did observe an increase in expression of genes involved in glycolysis. The RelA/p63 complex can also increase glucose uptake via transcriptional regulation of the glucose transporter GLUT3 [96]. However, no changes were found in GLUT3 expression levels in our data.

Work with *Saccharomyces cerevisiae*, *C. elegans* and *D*. *melanogaster* indicated that lifespan can also be extended by overexpression of Sir2 [47–49] which may also interact with the insulin signaling pathway [50]. Mammals have 7 Sir2-like proteins (i.e. SIRT1-7) [98] and the loss of SIRT6 in SIRT6-deficient mice leads to abnormalities that were similar to ageing-associated degenerative processes [99]. Here we found little changes in expression levels of SIRTs and only *Sirt4* and *Sirt7* were negatively correlated with the increase in CR level. SIRT4 knock out (KO) mice have a 30% increase in circulating levels of insulin during *ad libitum* feeding [100]. After an overnight fast, the KO mice had higher insulin levels compared to their wild type littermates which was not due to due to glucose intolerance. In fact the fasted KO mice had a slightly improved glucose tolerance [100]. During CR, SIRT4 activity was downregulated and Haigis et al (2006) postulates this is due to the switch towards amino acid-stimulated insulin secretion they identified in the KO mice. This is in agreement with our results were we also found a decrease in expression levels of SIRT4 with increasing CR. Here, we found that fatty acid β-oxidation is upregulated during CR, which is in agreement with others [101]. This would lead to a reduction in NAD/NADH ratio in the liver. Hence Haigis et al (2006) suggests that the change in this ratio may downregulate SIRT4 during CR as SIRT4 is dependent on NAD for its activity. In addition, SIRT4 deacetylates and inhibits malonyl CoA decarboxylase 1 (MCD1) and deletion of SIRT4 would therefore prevent production of a key precursor for fat synthesis (i.e. malonyl CoA) [102]. Hence SIRT4 downregulation during CR is linked to the changes in energy metabolism. SIRT7 was also negatively correlated with increasing levels of CR in our study. SIRT7 is mainly linked to maintenance of genome integrity and is associated with RNA polymerase I [103,104]. SIRT7 is highly expressed in tissues with dividing cells [104] and it was suggested that SIRT7 activity may be decreased during CR to restrain ribosome biogenesis and cell growth when energy is limited [105]. Short term 40% CR (30 days) had no impact on expression levels (both mRNA and protein) of hepatic SIRT7 of 4 months old and 24 months old Wistar rats [106]. However, after 3 months of CR we did found lower expression levels of SIRT7 with increasing levels of CR.

Insulin/IGF-1 and NF-ĸB pathways were strongly down-regulated at the transcriptional level in relation to CR. For mTOR there was some evidence of down-regulation. Although SIRT4 and SIRT7 was negatively correlated with the increase in CR, their role regulating lifespan are limited. Hence we postulate that SIRT signaling is not necessary for the benefical effects of CR on longevity.

### CR altered fuel utilization that may reduce ROS production, and antioxidant defenses

When mice were exposed to long-term CR (28 months) changes were found in the TCA cycle intermediates which might reflect an important adaptation to create available substrates for gluconeogenesis [107]. Levels of citrate, glutamate and alpha-ketoglutarate were decreased and levels of malate were increased [107]. Here we showed gluconeogenesis was increased under graded CR, which is in agreement with previous studies [108]. We found strong transcriptional upregulation of genes linked to glycolysis, the TCA cycle and the electron transport chain. This would indicate adaptation for an increased level of metabolism at the tissue level. This response is paradoxical because at the whole animal level there is less energy being supplied when mice are under restriction and hence there is a need to reduce energy expenditure at the whole animal level. These observations can be reconciled if the animal under CR disproportionately reduces the amount of tissues in its body to more than compensate for the reduced energy intake, providing scope to elevate the tissue level of expenditure [109,110]. For the same individual mice studied in the present paper we have shown that the level of basal metabolic rate [21] is consistent with the measured changes in body composition at the end of the restriction period [15]. In fact, the increased gene expression in the glycolysis/TCA cycle/ETC was negatively correlated with the measured BMR and average body temperature (Figure 11). The reported transcriptional changes in glycolysis/TCA cycle/ETC may then be more important to support changes in physical activity patterns. Supporting this view, in these same individual mice there was an intense period of physical activity prior to food being delivered each day (food anticipatory activity) [18] and transcriptional changes in the glycolysis/TCA cycle/ETC cycle were strongly positively correlated with the levels of FAA in the same individual mice (Figure 11).

One of the earliest theories of aging is that the accumulation of damage caused by ROS, via electron leakage from the ETC, leads to a gradual decline in cellular function [34]. We also found a shift toward fatty acid β-oxidation, which produces FADH thereby potentially mitigating ROS production [33]. After only 6 weeks of CR, H_2_O_2_ production (i.e. a component of the ROS) in hepatic mitochondria of rats significantly decreased. Although ROS production was decreased at complex I in CR rats, this was not significant compared to complex II [111]. Long term CR significantly decreased H_2_O_2_ production by 46% in hepatic mitochondria of rats at complex I [112]. In the present analysis transcription of elements of both complex II and complex IV were positively correlated with increasing CR. A decrease in the capacity of the later enzymes in the ETC can theoretically lead to accumulation of electrons in the upstream complexes increasing ROS production [113]. In mammalian brain and synaptic mitochondria of rats, decreased activity of complex IV was found with age [113,114]. In agreement with our results, previous studies have reported an increase in complex IV levels under CR in brain [115].

To further elaborate on the ROS theory, we previously measured the hepatic activity levels of enzymatic antioxidants (i.e. superoxide dismutase (SOD), glutathione peroxidase and catalase) [15] in livers of the same mice used in the present study. With increasing CR these three antioxidants had lower activity levels, which is potentially an adaptive response to the suggested reduction in ROS production under CR. This is also consistent with the fact that oxidative damage was unchanged in the same mice [15]. These data are consistent with the mice modulating their oxidative defense under CR, potentially to save energy, while maintaining damage levels constant.

We also investigated other pathways related to ROS and found eIF2 signaling was positively correlated and NRF2 negatively correlated with the level of CR. In response to cell stress eIF2 is phosphorylated and reduces formation of the eIF2-GTP complex. This lowers general translation and allows the cell to selectively enhance gene-specific translation [116]. Reduced translation of target genes by phosphorylated eIF2 can lower IĸB, which as noted above is an inhibitor of NF-ĸB [117]. The upregulation of eIF2 signaling is inconsistent with the reduced ROS production and decreased levels of NF-ĸB observed here. However, phosphorylation of eIF2 may be a direct effect of CR. Western blots showed that eIF2 is phosphorylated to signal nutrient deficiency in the anterior piriform cortex of the brains of rats to induce a behavior response [118]. Although, eIF2 was up-regulated in liver, it may reflect the response to state of decreased nutrient availably and not a response to cellular oxidative stress. NRF2 signaling, the second ROS-associated pathway in our data, induces expression of genes related to the anti-oxidant response (reviewed in [119]). This pathway is activated as a cellular defense mechanism against oxidative stress [120] and its observed downregulation in relation to graded CR would be consistent with reduced ROS production. Hence, the changes in NRF2 but not eIF2 signaling induced by graded CR are in agreement with the previously measured reduced activity levels of the enzymatic anti-oxidants.

Overall, the changes we observed in genes related to fuel utilization may contribute to reduced ROS production. However, the responses to this reduction including reduced defense, resulted in minimal impact on oxidative damage. Hence, these changes are unlikely to contribute significantly to the observed increased lifespan by CR. However, recent work has highlighted a role for solute carrier family 13 (sodium-dependent citrate transporter), member 5 (SLC13A5) in whole body lipid and glucose metabolism during ageing [121]. Liver specific knockdown of SLC13A5 in rats improves insulin sensitivity, reduces plasma insulin, lipid and amino acid levels, and mediates a trend towards decreased basal metabolic rates [121]. Hence SLC13A5 may be an interesting focus for future longevity studies.

### CR impacted MUPs genes and suggested a reduced reproductive investment

The disposable soma theory postulating a trade-off between investment in somatic maintenance and reproduction closes the gap between mechanistic and evolutionary theories of aging. In a natural environ-ment, too high investment in somatic maintenance is not beneficial if the organism dies from extrinsic mortality before it can breed, while too low protection might result in premature death. The disposable soma theory argues that during CR organisms need to reallocate the limited energy sources to maintain the soma and this requires diversion of resources away from reproduction [31,32]. MUPs are used in scent marking, and can bind molecules that are pheromonally active but are also used as signaling molecules. Male mice produce on average three to four times more urinary MUPs than female mice [62]. More than 99% of urinary proteins are MUPs. MUPs are primarily produced in the liver and about 20-30% of all the proteins produced by the liver in male mice are MUPs [122]. Hence production of MUPs is likely to be energetically costly and reducing their production may conserve energy for allocation to somatic protection [31,62,63]. Gene expression for 16 different MUPs were all down-regulated at 20CR, 30CR and 40CR compared to 12AL consistent with previous data showing decreased levels of urinary MUPs under CR in the same individual mice [15] and another study of mice under CR [63]. We have also shown that gene expression of MUPs in adipose tissue of the same individuals studied here was also reduced [123]

### CR had a positive impact on autophagy

During CR autophagy improves cellular survival and prevents cell death [124]. Previous work in humans has suggested that long-term CR leads to increased expression levels of autophagy related genes in skeletal muscle [125]. CR-induced autophagy leads to mice being more tolerant against chemotherapy induced cell damage [126]. Hence the beneficial effects of auto-phagy under CR seems to come in part from its ability to reduce the accumulation of damaged proteins. In addition, aging leads to damaged mitochondrial DNA (potentially ROS induced) and autophagy may remove these dysfunctional mitochondria [127]. In the present study, expression levels of 8 genes involved in autophagy were positively associated with the level of CR. When ATG7 is repressed in livers of lean mice, they develop severe insulin resistance [128], which also suggests a role for autophagy in insulin action. Circulating levels of insulin of the same mice indeed correlated with expression levels of genes involved in autophagy but not with *Atg7*. Hence increased autophagy may contribute to the improved insulin sensitivity we observed in these animals [15].

### Graded CR had a graded protective effect against cancer

A large impact of short-term CR was found on cancer-related pathways. The beneficial effects of CR on cancer are well known and has been reported extensively (reviewed in [53]). We observed reduced signaling of Hedgehog, TGF-β and CTNN-β with increasing CR. Hedgehog signaling plays a role in differentiation and determination of cell fate [129]. Inhibition of Hedgehog proteins in mice leads to decreased propagation of chronic myelogenous leukemia [130] and Hedgehog proteins are known to co-express with other oncogenetic pathways such as TGF-β and WNT signaling [131]. It has been proposed that leptin (strongly reduced in our mice with increasing CR levels [15]) plays an important role as a cell fate modulator via Hedgehog signaling in liver fibrosis and obesity-associated cancer metastasis [132]. TNF-α is known to stimulate TGF-β gene expression, and TGF-β is released by adipocytes [133]. TNF-α also had progressively reduced circulating levels as severity of CR increased [15]. Previous analysis of putative signaling proteins secreted by adipose tissue in the same mice included those related to TGF-β signaling [123]. Hence this may result in reduced TGF-β signaling to other tissues, which was in agreement with our hepatic results. Excess adipose tissue leads to increased secretion of IGF-1, IL6, leptin and TGF-α which can promote tumor growth [134,135]. Fat mass and circulating levels of IGF-1, IL6 and leptin were all reduced in the same mice in a linear manner to the level of CR [15].

The altered hepatic gene expression indicated a progressively increased protective effect of graded CR on cancer development, which is in agreement with a graded decrease in factors promoting tumor growth such as circulating leptin levels and fat mass in the same mice. Hence, the protective effect of graded CR on cancer development may result from a system-wide adaptation.

### CR had no impact on FGF21 and had a positive impact on H_2_S

FGF21 is a hormone secreted by liver during fasting, and can induce hepatic fatty acid β-oxidation and ketogenesis. In mice, overexpression of FGF21 leads to an increased lifespan without reducing food intake [54]. Zhang et al. postulated that FGF21 primarily acts by blunting the growth factor/IGF-1 signaling pathway [54]. Although fatty acid β–oxidation and ketogenesis were increased and IGF-1 signaling decreased, we did not observe any increase in gene expression of *Fgf21* in relation to restriction level. This is in stark contrast with protein and methionine restriction (PR and MR) studies where *Fgf21* expression was significantly increased compared to controls [136–138]. Our CR diet protocol involved simultaneous reductions in both calorie and protein intake [14]. Nevertheless, our diets maximally involved 40% protein restriction, while previous PR and MR studies that impacted *Fgf21* gene expression involved restrictions of around 80%. Hence if *Fgf21* is induced by extremely low protein intake rather than reduced calories this may explain the absence of a significant upregulation of *Fgf21* in our CR study. Since CR studies seldom exceed 40% restriction (reviewed in [139]) and at this level there is a highly significant impact on lifespan, these data suggest *Fgf21* is unlikely to be a significant mediator of the CR effect on lifespan. H_2_S is responsive to physiological stimuli and plays a signaling role in neural transmission, smooth muscle relaxation and can regulate release of insulin (reviewed in [140]). H_2_S activity is also related to oxidative stress and may play a protective role against oxidative damage [141–143]. The two enzymes CSE and CBS are necessary for the synthesis of H_2_S [144] and *Cth* was upregulated under CR but *Cbs* downregulated. H_2_S production via CBS is dependent on S-adenosyl-methionine [145] and CSE produces H_2_S via cysteine or homocysteine [146]. Cysteine and homocysteine were both upregulated in the liver metabolomics data of the same mice [22]. This might suggest more H_2_S production via CSE. Increased H_2_S production during CR induces signaling cascades leading to an activation of eIF2a (i.e. phosphorylated eIF2) and repression of mTOR [147,148]. This is in concordance with our results as we found an increase in eIF2 signaling and a decrease in mTORC1 and mTORC2 expression with increasing CR levels.

In hepatocytes, H_2_S may regulate expression of circadian clock genes and *Sirt1* [66]. We found an increased gene expression of the clock genes *Cry1*, *Per1* and *Per2,* which was in agreement with the expression levels we found in hypothalamus of the same mice [19]. Although the circadian rhythms in older mice remain, they lose the ability to synchronize with the environment which has a negative effect on longevity [149,150]. CR synchronizes these rhythms and may protect against the loss of circadian rhythm synchronization [151]. Although circadian rhythms are mainly established in the suprachiasmatic nucleus clock, the central pacemaker in the hypothalamus, studies have suggested that expression of circadian clock genes in the liver could be established independent of the hypothalamus [152–154]. *Per1* and *Per2* have tumor suppression activity [155], which may tie in with the anti-cancer mechanism under CR [53]. Overall our data are consistent with H_2_S signaling playing a key role in the impact of CR.

### CR leads to up regulation of the xenobiotic metabolism

Genes in this pathway play an important role in protection against environmental toxins and furthermore interact with phase II conjugation enzymes, which enhance hydrophilicity and excretion rate of environmental toxins [156]. These toxins are then excreted into bile via downstream phase III transporters [156]. Interestingly, long-lived ‘Little’ mice show resistance to oxidative toxins, and xenobiotic metabolism is upregulated, specifically xenobiotic detoxification genes [157,158]. With age, genes involved in xenobiotic metabolism are decreased in expression [159] and therefore preserved xenobiotic metabolism is believed to contribute to the increase in lifespan observed in long-lived mice. Similar to Ames dwarf and Little mice, our data showed upregulation of genes involved in xenobiotic metabolism under graded CR relative to 12AL which corresponded with previous research focused at single levels of restriction [101,160]. The decreased ability of the liver to metabolize drugs is believed to be mainly due by loss of expression of cytochrome P450 family 2B and 2C, which were among the genes significantly upregulated in our dataset [161]. Furthermore our data also showed genes involved in the ‘FXR/RXR activation’ pathway were upregulated as a response to CR, which are primary regulators of xenobiotic metabolism [162]. The bile acid receptor FXR could potentially mediate the upregulation of xenobiotic genes in the long-lived Little mice [158]. Although no causal link has been found between increased xenobiotic metabolism activity and pro-longevity, a study in *C. elegans* supports such a hypothesis [56]. It is still unclear how xenobiotic metabolism can increase lifespan but potentially it could be as simple as the reduction in damage caused by toxic compounds. The beneficial effects of xenobiotic metabolism may be signaled via reduced insulin/IGF-1 signaling under CR. Genes regulated by the insulin/IGF-1 pathway in *C. elegans* include the xeno-biotic detoxification genes [56]. Microarray analysis in long-lived Ames dwarf mice and Little mice suggest a similar role for insulin and IGF-1 in regulating xenobiotic metabolism [157]. It has been postulated that xenobiotic metabolism may be a key modulator of aging, separate from oxidative stress [56].

## MATERIALS AND METHODS

### Animals and experimental manipulations

All procedures were approved by the University of Aberdeen ethical approval committee and carried out under the Animals (Scientific Procedures) Act 1986 Home Office license (PPL 60/3706 held by JRS). Forty eight male C57BL/6 mice (*Mus musculus*) purchased from Charles River (Ormiston, UK) were individually housed with free access to water. Mice were exposed to 12 hour dark/light cycle (lights on at 0630h) and body mass and food intake were recorded daily, immediately prior to nocturnal feeding. At 20 weeks of age (resembling early adulthood in human), mice were randomly allocated into 6 different treatment groups: (12AL n=8, 24AL n=8, 10CR n=8, 20CR n=8, 30CR n=7, 40CR n=9). Mice in 24AL and 12AL group were fed *ad libitum* for 24h or 12h during the dark period respectively. 10CR, 20CR, 30CR and 40CR indicates 10 %, 20 %, 30 % and 40 % lower calories respectively than their own individual intakes measured over a baseline period of 14 days prior to introducing CR.

Animals fed completely *ad libitum* (i.e., having 24 hours access to food) may over feed and become obese. CR associated changes compared to 24AL are therefore most likely to reflect the anti-obesity effect of CR [4,163]. In addition, CR-restricted mice generally consume food during the first few hours of food provided. The 24AL animals can by definition eat at any time throughout a 24h period. Hence, when CR-restricted mice were culled they may have been starving for 10h-16h while 24AL may have eaten in the hour prior to culling. To address this issue, 12AL was set as a reference to avoid the “time since last meal effect” and graded levels of CR were introduced to investigate a potential graded response. Information on overall study design, diet composition and detailed rationale are described elsewhere [14].

### RNA isolation, cDNA synthesis and RNA sequencing

After culling by a terminal CO_2_ overdose the liver was removed as part of the overall dissection [14], weighed, divided into 7 pieces which were immediately snap frozen in liquid nitrogen and stored in -80°C until one piece was used for RNA isolation. RNA was isolated by homogenizing in Tri-Reagent (Sigma Aldrich, UK) according to manufacturer's instructions. Prior to RNA quantification by Agilent RNA 6000 Nano Kit samples were denatured at 65°C. RNA of 37 individual mice was successfully isolated and sent to Beijing Genomic Institute (BGI, Hong Kong) for RNA sequencing.

Library preparation was conducted by enriching total RNA by using oligo(dT) magnetic beads. The fragmentation buffer was added to obtain short fragments from the RNA. The mRNA was used as template for the random hexamer primers which synthesizes the first strand of cDNA. The second strand was synthesized by adding buffer dNTPs, RNase and DNA polymerase. QiaQuick PCR extraction kit was used to purify the double stranded cDNA and washed with an elution buffer for end repair and single nucleotide A addition. The fragments were ligated with sequencing adaptors and purified by agarose gel-electrophoresis to obtain the correct fragments. These were enriched by PCR amplification. During the quality control step, Agilent 2100 Bioanalyser and ABI StepOnePlus Real-Time PCR System are used to qualify and quantify of the sample library. The library products were sequenced using an Illumina Hi-seq 2000 resulting in 50 bp single ended reads (standard protocol BGI, Hong Kong). Standard primers and barcodes developed by BGI were used.

### Alignment to the reference genome

Prior to alignment to the reference genome, FASTQ files were quality controlled to identify the presence of adaptors or low quality sequences using fastQC (http://www.bioinformatics.bbsrc.ac.uk/projects/fastqc/). To ensure high sequencing quality, the reads were trimmed with a cut-off phred score of 28 using Trimmomatic [164]. Reads were aligned to the reference genome obtained from the National Center for Biotechnology Information (NCBI) database (Mus musculus, version MGSCv37, 2010/09/23, http://www.ncbi.nlm.nih.gov/assembly/165668/). The reference genome was indexed using Bowtie2 [165] and reads aligned with Tophat2 [166] using default settings. Of the 492,199,393 reads 482,311,031 (97.99%) were successfully aligned to the reference genome. 15.6 % contained multi mapped reads which were removed using the Sequence Alignment/Map (SAM) tool [167] before proceeding to quantification of the reads. Aligned sequencing reads were counted with HTSeq-count [168] by identification of how many reads mapped onto a single feature (genes containing exons).

### Analytical procedure

To remove any genes that exhibited no or a very low number of mapped reads, only genes that had more than 1 count per million in at least 4 samples across all treatments were retained for further analysis. This resulted in a total of 12,183 unique genes. Read counts were normalized using the trimmed mean of M values (TMM normalization) [169] to account for highly expressed genes consuming a substantial proportion of the total library size. This composition effect would cause remaining genes to be under sampled [170]. Differential gene expression was modeled using the edgeR package [170] in R (version 3.1.2) [171] and pairwise comparisons were conducted between 12AL and 24AL and between 12AL and each level of CR. Significant genes were identified based on a cut off p-value < 0.05 and an absolute log fold change (log FC) of 1. We identified differentially expressed genes (DEGs) relative to 12AL based on the adjusted p-value (FDR < 0.05). At 24AL, 10CR and 20CR none of the genes were differently expressed relative to 12AL. At 30CR only four genes had a FDR lower than 0.05 and at 40CR this massively increased to 855. We therefore used a cut-off value of absolute log_2_ fold changes (log FC) > 1.00 and p-value < 0.05. The amount of DEGs relative to 12AL responded to CR in a graded manner (24AL, 10CR, 20CR, 30CR and 40CR): 88, 138, 533, 316 and 608 respectively.

### Biological interpretation

The normalized counts for each gene were correlated with the increase in CR level by Pearson correlation method in the statistical environment R (version 3.1.2) [171]. The correlation coefficient for all genes (n=12,183) and their associated logFC relative to 12AL was then loaded into the IPA program (version 2000-2016, Ingenuity® Systems, www.ingenuity.com) to visualize the associated changes with CR in the aging pathways. The insulin/IGF-1 signaling pathway was constructed by merging the “Insulin Receptor Signaling” and “IGF-1 signaling”. For the mTOR signaling pathway, the “mTOR signaling” was used. The pathways “NF-ĸB Signaling” and “NF-ĸB Activation by Viruses” were merged to obtain the NF-ĸB signaling pathway. IPA did not have a prebuilt sirtuin signaling pathway. We therefore manually constructed this pathway based on the summarized data by Nakagawa and Guarente (2011) [61]. The sirtuin signaling pathway was manually constructed by using the built-in Path Designer function. For the oxidative stress pathway, pathways classified as “Cellular Stress and Injury” were merged with the pathway “Production of Nitric Oxide and Reactive Oxygen Species in Macrophages”. The reproduction pathway was built by merging “Germ Cell-Sertoli Cell Junction Signaling”, “Sertoli Cell-Sertoli Cell Junction Signaling”, MUP genes and the function reproduction. For the cancer signaling, autophagy pathway and xenobiotic metabolism, the “Molecular Mechanism of Cancer”, “Autophagy”, “Xenobiotic Metabolism Signaling” were used. For the changes in fuel utilization, the pathways “Glycolysis I”, “TCA cycle II (Eukaryotic)”, “Fatty Acid β-oxidation I” and “Gluconeogenesis I” were used. Mitochondrial biogenesis was obtained by merging “Mitochondrial Dysfunction” and the function mito-chondrial biogenesis. Lastly the H_2_S pathways were manually constructed. The p-value is calculated using the right-tailed Fisher Exact Test. The z-score predicts the activation state (i.e. increased or decreased) of a pathway based on the significant patterns that match the curated knowledge of gene expression levels and the causal relations with the pathway. Hence a pathway may have a significant p-value but lack a z-score if the expression levels of genes does not match the pre-defined associated regulation in that particular pathway. The custom built pathways do not have a z-score and hence we do not have a prediction of their down or upregulation calculated by IPA. Physiological data and behavior al data (methods and data described in [15,16,18,21]) were correlated with each gene and each individual using Pearson correlations conducted in the statistical environment R (version 3.2.5) [171].

## SUPPLEMENTARY MATERIAL TABLES



## References

[1] Tereshina EV (2009). Metabolic abnormalities as a basis for age-dependent diseases and aging? State of the art. Adv Gerontol.

[2] McCay C, Crowell M, Maynard L (1935). The effect of retarded growth upon the length of life span and upon the ultimate body size. J Nutr.

[3] Speakman JR, Hambly C (2007). Starving for life: what animal studies can and cannot tell us about the use of caloric restriction to prolong human lifespan. J Nutr.

[4] Speakman JR, Mitchell SE (2011). Caloric restriction. Mol Aspects Med.

[5] Fabrizio P, Longo VD (2003). The chronological life span of Saccharomyces cerevisiae. Aging Cell.

[6] Bross TG, Rogina B, Helfand SL (2005). Behavioral, physical, and demographic changes in Drosophila populations through dietary restriction. Aging Cell.

[7] Burger JM, Buechel SD, Kawecki TJ (2010). Dietary restriction affects lifespan but not cognitive aging in Drosophila melanogaster. Aging Cell.

[8] Colman RJ, Beasley TM, Kemnitz JW, Johnson SC, Weindruch R, Anderson RM (2014). Caloric restriction reduces age-related and all-cause mortality in rhesus monkeys. Nat Commun.

[9] Mattison JA, Colman RJ, Beasley TM, Allison DB, Kemnitz JW, Roth GS, Ingram DK, Weindruch R, de Cabo R, Anderson RM, Gibbs RA, Zimin AV, Bowden DM (2017). Caloric restriction improves health and survival of rhesus monkeys. Nat Commun.

[10] Mercken EM, Crosby SD, Lamming DW, JeBailey L, Krzysik-Walker S, Villareal DT, Capri M, Franceschi C, Zhang Y, Becker K, Sabatini DM, de Cabo R, Fontana L (2013). Calorie restriction in humans inhibits the PI3K/AKT pathway and induces a younger transcription profile. Aging Cell.

[11] Fontana L, Meyer TE, Klein S, Holloszy JO (2004). Long-term calorie restriction is highly effective in reducing the risk for atherosclerosis in humans. Proc Natl Acad Sci USA.

[12] Fontana L, Klein S, Holloszy JO (2010). Effects of long-term calorie restriction and endurance exercise on glucose tolerance, insulin action, and adipokine production. Age (Dordr).

[13] Koopman JJ, van Heemst D, van Bodegom D, Bonkowski MS, Sun LY, Bartke A (2016). Measuring ageing rates of mice subjected to caloric restriction and genetic disruption of growth hormone signaling. Aging (Albany NY).

[14] Mitchell SE, Tang Z, Kerbois C, Delville C, Konstantopedos P, Bruel A, Derous D, Green C, Aspden RM, Goodyear SR, Chen L, Han JJ, Wang Y (2015). The effects of graded levels of calorie restriction: I. impact of short term calorie and protein restriction on body composition in the C57BL/6 mouse. Oncotarget.

[15] Mitchell SE, Delville C, Konstantopedos P, Hurst J, Derous D, Green C, Chen L, Han JJ, Wang Y, Promislow DE, Lusseau D, Douglas A, Speakman JR (2015). The effects of graded levels of calorie restriction: II. Impact of short term calorie and protein restriction on circulating hormone levels, glucose homeostasis and oxidative stress in male C57BL/6 mice. Oncotarget.

[16] Mitchell SE, Delville C, Konstantopedos P, Derous D, Green CL, Chen L, Han JD, Wang Y, Promislow DE, Douglas A, Lusseau D, Speakman JR (2015). The effects of graded levels of calorie restriction: III. Impact of short term calorie and protein restriction on mean daily body temperature and torpor use in the C57BL/6 mouse. Oncotarget.

[17] Lusseau D, Mitchell SE, Barros C, Derous D, Green C, Chen L, Han JD, Wang Y, Promislow DE, Douglas A, Speakman JR (2015). The effects of graded levels of calorie restriction: IV. Non-linear change in behavioural phenotype of mice in response to short-term calorie restriction. Sci Rep.

[18] Mitchell SE, Delville C, Konstantopedos P, Derous D, Green CL, Wang Y, Han JD, Promislow DE, Douglas A, Chen L, Lusseau D, Speakman JR (2016). The effects of graded levels of calorie restriction: V. Impact of short term calorie and protein restriction on physical activity in the C57BL/6 mouse. Oncotarget.

[19] Derous D, Mitchell SE, Green CL, Chen L, Han JD, Wang Y, Promislow DE, Lusseau D, Speakman JR, Douglas A (2016). The effects of graded levels of calorie restriction: VI. Impact of short-term graded calorie restriction on transcriptomic responses of the hypothalamic hunger and circadian signaling pathways. Aging (Albany NY).

[20] Derous D, Mitchell SE, Green CL, Wang Y, Han JD, Chen L, Promislow DE, Lusseau D, Speakman JR, Douglas A (2016). The effects of graded levels of calorie restriction: VII. Topological rearrangement of hypothalamic aging networks. Aging (Albany NY).

[21] Mitchell SE, Tang Z, Kerbois C, Delville C, Derous D, Green CL, Wang Y, Han JJ, Chen L, Douglas A, Lusseau D, Promislow DE, Speakman JR (2017). The effects of graded levels of calorie restriction: VIII. Impact of short term calorie and protein restriction on basal metabolic rate in the C57BL/6 mouse. Oncotarget.

[22] Green CL, Mitchell SE, Derous D, Wang Y, Chen L, Han JJ, Promislow DE, Lusseau D, Douglas A, Speakman JR (2017). The effects of graded levels of calorie restriction: IX. Global metabolomic screen reveals modulation of carnitines, sphingolipids and bile acids in the liver of C57BL/6 mice. Aging Cell.

[23] Schmucker DL (1998). Aging and the liver: an update. J Gerontol A Biol Sci Med Sci.

[24] Cao SX, Dhahbi JM, Mote PL, Spindler SR (2001). Genomic profiling of short- and long-term caloric restriction effects in the liver of aging mice. Proc Natl Acad Sci USA.

[25] Medvedev ZA (1990). An attempt at a rational classification of theories of ageing. Biol Rev Camb Philos Soc.

[26] Kuhla A, Hahn S, Butschkau A, Lange S, Wree A, Vollmar B (2014). Lifelong caloric restriction reprograms hepatic fat metabolism in mice. J Gerontol A Biol Sci Med Sci.

[27] Kuhla A, Blei T, Jaster R, Vollmar B (2011). Aging is associated with a shift of fatty metabolism toward lipogenesis. J Gerontol A Biol Sci Med Sci.

[28] Badman MK, Pissios P, Kennedy AR, Koukos G, Flier JS, Maratos-Flier E (2007). Hepatic fibroblast growth factor 21 is regulated by PPARalpha and is a key mediator of hepatic lipid metabolism in ketotic states. Cell Metab.

[29] Gerhart-Hines Z, Rodgers JT, Bare O, Lerin C, Kim SH, Mostoslavsky R, Alt FW, Wu Z, Puigserver P (2007). Metabolic control of muscle mitochondrial function and fatty acid oxidation through SIRT1/PGC-1alpha. EMBO J.

[30] Takemori K, Kimura T, Shirasaka N, Inoue T, Masuno K, Ito H (2011). Food restriction improves glucose and lipid metabolism through Sirt1 expression: a study using a new rat model with obesity and severe hypertension. Life Sci.

[31] Kirkwood TB (1977). Evolution of ageing. Nature.

[32] Kowald A, Kirkwood TB (2015). Evolutionary significance of ageing in the wild. Exp Gerontol.

[33] Hirst J, King MS, Pryde KR (2008). The production of reactive oxygen species by complex I. Biochem Soc Trans.

[34] Harman D (1956). Aging: a theory based on free radical and radiation chemistry. J Gerontol.

[35] Argentino DP, Dominici FP, Al-Regaiey K, Bonkowski MS, Bartke A, Turyn D (2005). Effects of long-term caloric restriction on early steps of the insulin-signaling system in mouse skeletal muscle. J Gerontol A Biol Sci Med Sci.

[36] Breese CR, Ingram RL, Sonntag WE (1991). Influence of age and long-term dietary restriction on plasma insulin-like growth factor-1 (IGF-1), IGF-1 gene expression, and IGF-1 binding proteins. J Gerontol.

[37] Johnson SC, Rabinovitch PS, Kaeberlein M (2013). mTOR is a key modulator of ageing and age-related disease. Nature.

[38] Tilstra JS, Clauson CL, Niedernhofer LJ, Robbins PD (2011). NF-κB in Aging and Disease. Aging Dis.

[39] Houtkooper RH, Pirinen E, Auwerx J (2012). Sirtuins as regulators of metabolism and healthspan. Nat Rev Mol Cell Biol.

[40] Wolkow CA, Kimura KD, Lee MS, Ruvkun G (2000). Regulation of C. elegans life-span by insulinlike signaling in the nervous system. Science.

[41] Kimura KD, Tissenbaum HA, Liu Y, Ruvkun G (1997). daf-2, an insulin receptor-like gene that regulates longevity and diapause in Caenorhabditis elegans. Science.

[42] Blüher M, Michael MD, Peroni OD, Ueki K, Carter N, Kahn BB, Kahn CR (2002). Adipose tissue selective insulin receptor knockout protects against obesity and obesi-ty-related glucose intolerance. Dev Cell.

[43] Selman C, Partridge L, Withers DJ (2011). Replication of extended lifespan phenotype in mice with deletion of insulin receptor substrate 1. PLoS One.

[44] Selman C, Lingard S, Choudhury AI, Batterham RL, Claret M, Clements M, Ramadani F, Okkenhaug K, Schuster E, Blanc E, Piper MD, Al-Qassab H, Speakman JR (2008). Evidence for lifespan extension and delayed age-related biomarkers in insulin receptor substrate 1 null mice. FASEB J.

[45] Stanfel MN, Shamieh LS, Kaeberlein M, Kennedy BK (2009). The TOR pathway comes of age. Biochimica et Biophysica Acta - General Subjects.

[46] Kanfi Y, Naiman S, Amir G, Peshti V, Zinman G, Nahum L, Bar-Joseph Z, Cohen HY (2012). The sirtuin SIRT6 regulates lifespan in male mice. Nature.

[47] Giannakou ME, Goss M, Jünger MA, Hafen E, Leevers SJ, Partridge L (2004). Long-lived Drosophila with overexpressed dFOXO in adult fat body. Science.

[48] Kaeberlein M, McVey M, Guarente L (1999). The SIR2/3/4 complex and SIR2 alone promote longevity in Saccharomyces cerevisiae by two different mechanisms. Genes Dev.

[49] Tissenbaum HA, Guarente L (2001). Increased dosage of a sir-2 gene extends lifespan in Caenorhabditis elegans. Nature.

[50] Kenyon C, Chang J, Gensch E, Rudner A, Tabtiang R (1993). A C. elegans mutant that lives twice as long as wild type. Nature.

[51] Giannakou ME, Partridge L (2004). The interaction between FOXO and SIRT1: tipping the balance towards survival. Trends Cell Biol.

[52] Morselli E, Maiuri MC, Markaki M, Megalou E, Pasparaki A, Palikaras K, Criollo A, Galluzzi L, Malik SA, Vitale I, Michaud M, Madeo F, Tavernarakis N, Kroemer G (2010). Caloric restriction and resveratrol promote longevity through the Sirtuin-1-dependent induction of autophagy. Cell Death Dis.

[53] Longo VD, Fontana L (2010). Calorie restriction and cancer prevention: metabolic and molecular mechanisms. Trends Pharmacol Sci.

[54] Zhang Y, Xie Y, Berglund ED, Coate KC, He TT, Katafuchi T, Xiao G, Potthoff MJ, Wei W, Wan Y, Yu RT, Evans RM, Kliewer SA, Mangelsdorf DJ (2012). The starvation hormone, fibroblast growth factor-21, extends lifespan in mice. eLife.

[55] Zhang Y, Tang ZH, Ren Z, Qu SL, Liu MH, Liu LS, Jiang ZS (2013). Hydrogen sulfide, the next potent preventive and therapeutic agent in aging and age-associated diseases. Mol Cell Biol.

[56] McElwee JJ, Schuster E, Blanc E, Thomas JH, Gems D (2004). Shared transcriptional signature in Caenorhabditis elegans Dauer larvae and long-lived daf-2 mutants implicates detoxification system in longevity assurance. J Biol Chem.

[57] Merry BJ (2002). Molecular mechanisms linking calorie restriction and longevity. Int J Biochem Cell Biol.

[58] Duffy PH, Lewis SM, Mayhugh MA, Trotter RW, Latendresse JR, Thorn BT, Feuers RJ (2004). The effects of different levels of dietary restriction on neoplastic pathology in the male Sprague-Dawley rat. Aging Clin Exp Res.

[59] Nogueira LM, Lavigne JA, Chandramouli GV, Lui H, Barrett JC, Hursting SD (2012). Dose-dependent effects of calorie restriction on gene expression, metabolism, and tumor progression are partially mediated by insulin-like growth factor-1. Cancer Med.

[60] Kim SS, Choi KM, Kim S, Park T, Cho IC, Lee JW, Lee CK (2016). Whole-transcriptome analysis of mouse adipose tissue in response to short-term caloric restriction. Mol Genet Genomics.

[61] Nakagawa T, Guarente L (2011). Sirtuins at a glance. J Cell Sci.

[62] Beynon RJ, Hurst JL (2004). Urinary proteins and the modulation of chemical scents in mice and rats. Peptides.

[63] Mitchell SJ, Madrigal-Matute J, Scheibye-Knudsen M, Fang E, Aon M, González-Reyes JA, Cortassa S, Kaushik S, Gonzalez-Freire M, Patel B, Wahl D, Ali A, Calvo-Rubio M (2016). Effects of Sex, Strain, and Energy Intake on Hallmarks of Aging in Mice. Cell Metab.

[64] Hu W, Feng Z, Teresky AK, Levine AJ (2007). p53 regulates maternal reproduction through LIF. Nature.

[65] Levine AJ, Tomasini R, McKeon FD, Mak TW, Melino G (2011). The p53 family: guardians of maternal reproduc-tion. Nat Rev Mol Cell Biol.

[66] Shang Z, Lu C, Chen S, Hua L, Qian R (2012). Effect of H2S on the circadian rhythm of mouse hepatocytes. Lipids Health Dis.

[67] Turturro A, Duffy P, Hass B, Kodell R, Hart R (2002). Survival characteristics and age-adjusted disease incidences in C57BL/6 mice fed a commonly used cereal-based diet modulated by dietary restriction. J Gerontol A Biol Sci Med Sci.

[68] Paradis S, Ruvkun G (1998). Caenorhabditis elegans Akt/PKB transduces insulin receptor-like signals from AGE-1 PI3 kinase to the DAF-16 transcription factor. Genes Dev.

[69] Holland R, Hardie DG (1985). Both insulin and epidermal growth factor stimulate fatty acid synthesis and increase phosphorylation of acetyl-CoA carboxylase and ATP-citrate lyase in isolated hepatocytes. FEBS Lett.

[70] Fromenty B, Robin MA, Igoudjil A, Mansouri A, Pessayre D (2004). The ins and outs of mitochondrial dysfunction in NASH. Diabetes Metab.

[71] van Heemst D (2010). Insulin, IGF-1 and longevity. Aging Dis.

[72] Lambert AJ, Portero-Otin M, Pamplona R, Merry BJ (2004). Effect of ageing and caloric restriction on specific markers of protein oxidative damage and membrane peroxidizability in rat liver mitochondria. Mech Ageing Dev.

[73] Lambert AJ, Wang B, Yardley J, Edwards J, Merry BJ (2004). The effect of aging and caloric restriction on mitochondrial protein density and oxygen consumption. Exp Gerontol.

[74] Tucci P (2012). Caloric restriction: is mammalian life extension linked to p53?. Aging (Albany NY).

[75] Gems D (2007). Long-lived dwarf mice: are bile acids a longevity signal?. Aging Cell.

[76] Burks DJ, Font de Mora J, Schubert M, Withers DJ, Myers MG, Towery HH, Altamuro SL, Flint CL, White MF (2000). IRS-2 pathways integrate female reproduction and energy homeostasis. Nature.

[77] Sliwowska JH, Fergani C, Gawałek M, Skowronska B, Fichna P, Lehman MN (2014). Insulin: its role in the central control of reproduction. Physiol Behav.

[78] Kaaks R, Lukanova A (2001). Energy balance and cancer: the role of insulin and insulin-like growth factor-I. Proc Nutr Soc.

[79] Jia K, Chen D, Riddle DL (2004). The TOR pathway interacts with the insulin signaling pathway to regulate C. elegans larval development, metabolism and life span. Development.

[80] Vellai T, Takacs-Vellai K, Zhang Y, Kovacs AL, Orosz L, Müller F (2003). Genetics: influence of TOR kinase on lifespan in C. elegans. Nature.

[81] Katewa SD, Kapahi P (2011). Role of TOR signaling in aging and related biological processes in Drosophila melanogaster. Exp Gerontol.

[82] Bjedov I, Toivonen JM, Kerr F, Slack C, Jacobson J, Foley A, Partridge L (2010). Mechanisms of life span extension by rapamycin in the fruit fly Drosophila melanogaster. Cell Metab.

[83] Harrison DE, Strong R, Sharp ZD, Nelson JF, Astle CM, Flurkey K, Nadon NL, Wilkinson JE, Frenkel K, Carter CS, Pahor M, Javors MA, Fernandez E, Miller RA (2009). Rapamycin fed late in life extends lifespan in genetically heterogeneous mice. Nature.

[84] Miller RA, Harrison DE, Astle CM, Baur JA, Boyd AR, de Cabo R, Fernandez E, Flurkey K, Javors MA, Nelson JF, Orihuela CJ, Pletcher S, Sharp ZD (2011). Rapamycin, but not resveratrol or simvastatin, extends life span of genetically heterogeneous mice. J Gerontol A Biol Sci Med Sci.

[85] Kenyon CJ (2010). The genetics of ageing. Nature.

[86] Lamming DW, Ye L, Katajisto P, Goncalves MD, Saitoh M, Stevens DM, Davis JG, Salmon AB, Richardson A, Ahima RS, Guertin DA, Sabatini DM, Baur JA (2012). Rapamycin-induced insulin resistance is mediated by mTORC2 loss and uncoupled from longevity. Science.

[87] Selman C, Tullet JM, Wieser D, Irvine E, Lingard SJ, Choudhury AI, Claret M, Al-Qassab H, Carmignac D, Ramadani F, Woods A, Robinson IC, Schuster E (2009). Ribosomal protein S6 kinase 1 signaling regulates mammalian life span. Science.

[88] Sharp ZD, Strong R (2010). The role of mTOR signaling in controlling mammalian life span: what a fungicide teaches us about longevity. J Gerontol A Biol Sci Med Sci.

[89] Dufner A, Thomas G (1999). Ribosomal S6 kinase signaling and the control of translation. Exp Cell Res.

[90] Tsai SY, Rodriguez AA, Dastidar SG, Del Greco E, Carr KL, Sitzmann JM, Academia EC, Viray CM, Martinez LL, Kaplowitz BS, Ashe TD, La Spada AR, Kennedy BK (2016). Increased 4E-BP1 Expression Protects against Diet-Induced Obesity and Insulin Resistance in Male Mice. Cell Reports.

[91] Adler AS, Sinha S, Kawahara TL, Zhang JY, Segal E, Chang HY (2007). Motif module map reveals enforcement of aging by continual NF-kappaB activity. Genes Dev.

[92] Ghosh S, May MJ, Kopp EB (1998). NF-kappa B and Rel proteins: evolutionarily conserved mediators of immune responses. Annu Rev Immunol.

[93] Kim HJ, Jung KJ, Yu BP, Cho CG, Choi JS, Chung HY (2002). Modulation of redox-sensitive transcription factors by calorie restriction during aging. Mech Ageing Dev.

[94] Pahl HL (1999). Activators and target genes of Rel/NF-kappaB transcription factors. Oncogene.

[95] Mauro C, Leow SC, Anso E, Rocha S, Thotakura AK, Tornatore L, Moretti M, De Smaele E, Beg AA, Tergaonkar V, Chandel NS, Franzoso G (2011). NF-κB controls energy homeostasis and metabolic adaptation by upregulating mitochondrial respiration. Nat Cell Biol.

[96] Kawauchi K, Araki K, Tobiume K, Tanaka N (2008). p53 regulates glucose metabolism through an IKK-NF-kappaB pathway and inhibits cell transformation. Nat Cell Biol.

[97] Johnson RF, Witzel II, Perkins ND (2011). p53-dependent regulation of mitochondrial energy production by the RelA subunit of NF-κB. Cancer Res.

[98] Frye RA (2000). Phylogenetic classification of prokaryotic and eukaryotic Sir2-like proteins. Biochem Biophys Res Commun.

[99] Mostoslavsky R, Chua KF, Lombard DB, Pang WW, Fischer MR, Gellon L, Liu P, Mostoslavsky G, Franco S, Murphy MM, Mills KD, Patel P, Hsu JT (2006). Genomic instability and aging-like phenotype in the absence of mammalian SIRT6. Cell.

[100] Haigis MC, Mostoslavsky R, Haigis KM, Fahie K, Christodoulou DC, Murphy AJ, Valenzuela DM, Yancopoulos GD, Karow M, Blander G, Wolberger C, Prolla TA, Weindruch R (2006). SIRT4 inhibits glutamate dehydrogenase and opposes the effects of calorie restriction in pancreatic beta cells. Cell.

[101] Tsuchiya T, Dhahbi JM, Cui X, Mote PL, Bartke A, Spindler SR (2004). Additive regulation of hepatic gene expression by dwarfism and caloric restriction. Physiol Genomics.

[102] Laurent G, German NJ, Saha AK, de Boer VC, Davies M, Koves TR, Dephoure N, Fischer F, Boanca G, Vaitheesvaran B, Lovitch SB, Sharpe AH, Kurland IJ (2013). SIRT4 coordinates the balance between lipid synthesis and catabolism by repressing malonyl CoA decarboxylase. Mol Cell.

[103] Vazquez BN, Thackray JK, Serrano L (2017). Sirtuins and DNA damage repair: SIRT7 comes to play. Nucleus.

[104] Ford E, Voit R, Liszt G, Magin C, Grummt I, Guarente L (2006). Mammalian Sir2 homolog SIRT7 is an activator of RNA polymerase I transcription. Genes Dev.

[105] Guarente L (2006). Sirtuins as potential targets for metabolic syndrome. Nature.

[106] Wronska A, Lawniczak A, Wierzbicki PM, Kmiec Z (2016). Age-Related Changes in Sirtuin 7 Expression in Calorie-Restricted and Refed Rats. Gerontology.

[107] Hagopian K, Ramsey JJ, Weindruch R (2004). Krebs cycle enzymes from livers of old mice are differentially regulated by caloric restriction. Exp Gerontol.

[108] Hagopian K, Ramsey JJ, Weindruch R (2003). Caloric restriction increases gluconeogenic and transaminase enzyme activities in mouse liver. Exp Gerontol.

[109] Speakman JR, Selman C, McLaren JS, Harper EJ (2002). Living fast, dying when? The link between aging and energetics. J Nutr.

[110] Selman C, Phillips T, Staib JL, Duncan JS, Leeuwenburgh C, Speakman JR (2005). Energy expenditure of calorically restricted rats is higher than predicted from their altered body composition. Mech Ageing Dev.

[111] Gredilla R, Barja G, López-Torres M (2001). Effect of short-term caloric restriction on H2O2 production and oxidative DNA damage in rat liver mitochondria and location of the free radical source. J Bioenerg Biomembr.

[112] López-Torres M, Gredilla R, Sanz A, Barja G, López-Torres M, Gredilla R, Sanz A, Barja G (2002). Influence of aging and long-term caloric restriction on oxygen radical generation and oxidative DNA damage in rat liver mitochondria. Free Radic Biol Med.

[113] Ames BN, Shigenaga MK, Hagen TM (1995). Mitochondrial decay in aging. Biochim Biophys Acta.

[114] Allen RG, Keogh BP, Tresini M, Gerhard GS, Volker C, Pignolo RJ, Horton J, Cristofalo VJ (1997). Development and age-associated differences in electron transport potential and consequences for oxidant generation. JBiol Chem.

[115] Olgun A, Akman S, Serdar MA, Kutluay T (2002). Oxidative phosphorylation enzyme complexes in caloric restriction. Exp Gerontol.

[116] Wek RC, Jiang HY, Anthony TG (2006). Coping with stress: eIF2 kinases and translational control. Biochem Soc Trans.

[117] Jiang HY, Wek SA, McGrath BC, Scheuner D, Kaufman RJ, Cavener DR, Wek RC (2003). Phosphorylation of the alpha subunit of eukaryotic initiation factor 2 is required for activation of NF-kappaB in response to diverse cellular stresses. Mol Cell Biol.

[118] Gietzen DW, Ross CM, Hao S, Sharp JW (2004). Phosphorylation of eIF2alpha is involved in the signaling of indispensable amino acid deficiency in the anterior piriform cortex of the brain in rats. J Nutr.

[119] Ma Q (2013). Role of nrf2 in oxidative stress and toxicity. Annu Rev Pharmacol Toxicol.

[120] Nguyen T, Nioi P, Pickett CB (2009). The Nrf2-antioxidant response element signaling pathway and its activation by oxidative stress. J Biol Chem.

[121] Pesta DH, Perry RJ, Guebre-Egziabher F, Zhang D, Jurczak M, Fischer-Rosinsky A, Daniels MA, Willmes DM, Bhanot S, Bornstein SR, Knauf F, Samuel VT, Shulman GI, Birkenfeld AL (2015). Prevention of diet-induced hepatic steatosis and hepatic insulin resistance by second generation antisense oligonucleotides targeted to the longevity gene mIndy (Slc13a5). Aging Albany NY.

[122] Berger FG, Szoka P (1981). Biosynthesis of the major urinary proteins in mouse liver: a biochemical genetic study. Biochem Genet.

[123] Derous D, Mitchell SE, Green CL, Wang Y, Han JD, Chen L, Promislow DE, Lusseau D, Douglas A, Speakman JR (2017). The Effects of Graded Levels of Calorie Restriction: X. Transcriptomic Responses of Epididymal Adipose Tissue. J Gerontol A Biol Sci Med Sci.

[124] Tasdemir E, Maiuri MC, Galluzzi L, Vitale I, Djavaheri-Mergny M, D'Amelio M, Criollo A, Morselli E, Zhu C, Harper F, Nannmark U, Samara C, Pinton P (2008). Regulation of autophagy by cytoplasmic p53. Nat Cell Biol.

[125] Yang L, Licastro D, Cava E, Veronese N, Spelta F, Rizza W, Bertozzi B, Villareal DT, Hotamisligil GS, Holloszy JO, Fontana L (2015). Long-Term Calorie Restriction Enhances Cellular Quality-Control Processes in Human Skeletal Muscle. Cell Reports.

[126] Lee C, Safdie FM, Raffaghello L, Wei M, Madia F, Parrella E, Hwang D, Cohen P, Bianchi G, Longo VD (2010). Reduced levels of IGF-I mediate differential protection of normal and cancer cells in response to fasting and improve chemotherapeutic index. Cancer Res.

[127] Moreau K, Luo S, Rubinsztein DC (2010). Cytoprotective roles for autophagy. Curr Opin Cell Biol.

[128] Yang L, Li P, Fu S, Calay ES, Hotamisligil GS (2010). Defective hepatic autophagy in obesity promotes ER stress and causes insulin resistance. Cell Metab.

[129] King PJ, Guasti L, Laufer E (2008). Hedgehog signalling in endocrine development and disease. J Endocrinol.

[130] Zhao C, Chen A, Jamieson CH, Fereshteh M, Abrahamsson A, Blum J, Kwon HY, Kim J, Chute JP, Rizzieri D, Munchhof M, VanArsdale T, Beachy PA, Reya T (2009). Hedgehog signalling is essential for maintenance of cancer stem cells in myeloid leukaemia. Nature.

[131] Rubin LL, de Sauvage FJ (2006). Targeting the Hedgehog pathway in cancer. Nat Rev Drug Discov.

[132] Choi SS, Syn WK, Karaca GF, Omenetti A, Moylan CA, Witek RP, Agboola KM, Jung Y, Michelotti GA, Diehl AM (2010). Leptin promotes the myofibroblastic phenotype in hepatic stellate cells by activating the hedgehog pathway. J Biol Chem.

[133] Samad F, Yamamoto K, Pandey M, Loskutoff DJ (1997). Elevated expression of transforming growth factor-beta in adipose tissue from obese mice. Mol Med.

[134] Musi N, Guardado-Mendoza R (2014). Adipose Tissue as an Endocrine Organ. Cellular Endocrinology in Health and Disease.

[135] Park J, Scherer PE (2011). Leptin and cancer: from cancer stem cells to metastasis. Endocr Relat Cancer.

[136] Laeger T, Henagan TM, Albarado DC, Redman LM, Bray GA, Noland RC, Münzberg H, Hutson SM, Gettys TW, Schwartz MW, Morrison CD (2014). FGF21 is an endocrine signal of protein restriction. J Clin Invest.

[137] Lees EK, Król E, Grant L, Shearer K, Wyse C, Moncur E, Bykowska AS, Mody N, Gettys TW, Delibegovic M (2014). Methionine restriction restores a younger metabolic phenotype in adult mice with alterations in fibroblast growth factor 21. Aging Cell.

[138] Stone KP, Wanders D, Orgeron M, Cortez CC, Gettys TW (2014). Mechanisms of increased in vivo insulin sensitivity by dietary methionine restriction in mice. Diabetes.

[139] Speakman JR, Mitchell SE, Mazidi M (2016). Calories or protein? The effect of dietary restriction on lifespan in rodents is explained by calories alone. Exp Gerontol.

[140] Kimura H (2011). Hydrogen sulfide: its production, release and functions. Amino Acids.

[141] Whiteman M, Armstrong JS, Chu SH, Jia-Ling S, Wong BS, Cheung NS, Halliwell B, Moore PK (2004). The novel neuromodulator hydrogen sulfide: an endogenous peroxynitrite ‘scavenger’?. J Neurochem.

[142] Whiteman M, Li L, Kostetski I, Chu SH, Siau JL, Bhatia M, Moore PK (2006). Evidence for the formation of a novel nitrosothiol from the gaseous mediators nitric oxide and hydrogen sulphide. Biochem Biophys Res Commun.

[143] Kimura Y, Goto Y, Kimura H (2010). Hydrogen sulfide increases glutathione production and suppresses oxidative stress in mitochondria. Antioxid Redox Signal.

[144] Patel P, Vatish M, Heptinstall J, Wang R, Carson RJ (2009). The endogenous production of hydrogen sulphide in intrauterine tissues. Reprod Biol Endocrinol.

[145] Singh S, Padovani D, Leslie RA, Chiku T, Banerjee R (2009). Relative contributions of cystathionine beta-synthase and gamma-cystathionase to H2S biogenesis via alternative trans-sulfuration reactions. J Biol Chem.

[146] Chiku T, Padovani D, Zhu W, Singh S, Vitvitsky V, Banerjee R (2009). H2S biogenesis by human cystathionine gamma-lyase leads to the novel sulfur metabolites lanthionine and homolanthionine and is responsive to the grade of hyperhomocysteinemia. J Biol Chem.

[147] Hine C, Harputlugil E, Zhang Y, Ruckenstuhl C, Lee BC, Brace L, Longchamp A, Treviño-Villarreal JH, Mejia P, Ozaki CK, Wang R, Gladyshev VN, Madeo F (2015). Endogenous hydrogen sulfide production is essential for dietary restriction benefits. Cell.

[148] Robertson LT, Treviño-Villarreal JH, Mejia P, Grondin Y, Harputlugil E, Hine C, Vargas D, Zheng H, Ozaki CK, Kristal BS, Simpson SJ, Mitchell JR (2015). Protein and Calorie Restriction Contribute Additively to Protection from Renal Ischemia Reperfusion Injury Partly via Leptin Reduction in Male Mice. J Nutr.

[149] Taylor P, Weinert H, Weinert D, Waterhouse J (2010). The Circadian Activity and Body Temperature Rhythms of Mice During Their Last Days of Life. Biol Rhythm Res.

[150] Wyse CA, Coogan AN, Selman C, Hazlerigg DG, Speakman JR (2010). Association between mammalian lifespan and circadian free-running period: the circadian resonance hypothesis revisited. Biol Lett.

[151] Froy O, Chapnik N, Miskin R (2008). Relationship between calorie restriction and the biological clock: lessons from long-lived transgenic mice. Rejuvenation Res.

[152] Hara R, Wan K, Wakamatsu H, Aida R, Moriya T, Akiyama M, Shibata S (2001). Restricted feeding entrains liver clock without participation of the supra-chiasmatic nucleus. Genes Cells.

[153] Stokkan KA, Yamazaki S, Tei H, Sakaki Y, Menaker M (2001). Entrainment of the circadian clock in the liver by feeding. Science.

[154] Vollmers C, Gill S, DiTacchio L, Pulivarthy SR, Le HD, Panda S (2009). Time of feeding and the intrinsic circadian clock drive rhythms in hepatic gene expression. Proc Natl Acad Sci USA.

[155] Lee CC (2006). Tumor suppression by the mammalian Period genes. Cancer Causes Control.

[156] Omiecinski CJ, Vanden Heuvel JP, Perdew GH, Peters JM (2011). Xenobiotic metabolism, disposition, and regulation by receptors: from biochemical phenomenon to predictors of major toxicities. Toxicol Sci.

[157] Amador-Noguez D, Yagi K, Venable S, Darlington G (2004). Gene expression profile of long-lived Ames dwarf mice and Little mice. Aging Cell.

[158] Amador-Noguez D, Dean A, Huang W, Setchell K, Moore D, Darlington G (2007). Alterations in xenobiotic metabolism in the long-lived Little mice. Aging Cell.

[159] Mori K, Blackshear PE, Lobenhofer EK, Parker JS, Orzech DP, Roycroft JH, Walker KL, Johnson KA, Marsh TA, Irwin RD, Boorman GA (2007). Hepatic transcript levels for genes coding for enzymes associated with xenobiotic metabolism are altered with age. Toxicol Pathol.

[160] Steinbaugh MJ, Sun LY, Bartke A, Miller RA (2012). Activation of genes involved in xenobiotic metabolism is a shared signature of mouse models with extended lifespan. Am J Physiol Endocrinol Metab.

[161] Woodhouse K, Wynne HA (1992). Age-related changes in hepatic function. Implications for drug therapy. Drugs Aging.

[162] Meyer UA (2007). Endo-xenobiotic crosstalk and the regulation of cytochromes P450. Drug Metab Rev.

[163] Sohal RS, Forster MJ (2014). Caloric restriction and the aging process: a critique. Free Radic Biol Med.

[164] Bolger AM, Lohse M, Usadel B (2014). Trimmomatic: a flexible trimmer for Illumina sequence data. Bioinformatics.

[165] Langmead B, Trapnell C, Pop M, Salzberg SL (2009). Ultrafast and memory-efficient alignment of short DNA sequences to the human genome. Genome Biol.

[166] Trapnell C, Pachter L, Salzberg SL (2009). TopHat: discovering splice junctions with RNA-Seq. Bioinformatics.

[167] Li H, Handsaker B, Wysoker A, Fennell T, Ruan J, Homer N, Marth G, Abecasis G, Durbin R, 1000 Genome Project Data Processing Subgroup (2009). The Sequence Alignment/Map format and SAMtools. Bioinformatics.

[168] Anders S, Pyl PT, Huber W (2015). HTSeq--a Python framework to work with high-throughput sequencing data. Bioinformatics.

[169] Robinson MD, Oshlack A (2010). A scaling normalization method for differential expression analysis of RNA-seq data. Genome Biol.

[170] Robinson MD, McCarthy DJ, Smyth GK (2010). edgeR: a Bioconductor package for differential expression analysis of digital gene expression data. Bioinformatics.

[171] R Core Team (2014). R: a language and environment for statistical computing.

