# Supplementary material for: The effects of graded levels of calorie restriction: XI. Evaluation of the main hypotheses underpinning the life extension effects of CR using the hepatic transcriptome
Source: Aging (Albany NY). 2017 Jul 31;9(7):1770–804. doi: 10.18632/aging.101269 (PMC5559174; doi:10.18632/aging.101269)
Supplement: Supplementary file 1 [file aging-09-1770-s001.pdf]

SUPPLEMENTARY MATERIAL

Table S1. Expression levels of genes involved in insulin/IGF-1 signaling pathway correlated with the increase in calorie restriction (CR) and their gene expression at each level of CR relative to 12h ad libitum intake.

			10CR		20CR		30CR		40CR	
Symbol	Gene Symbol	*r*	*Log FC*	*p*	Log FC	p	Log FC	p	Log FC	p
4E-BP1	*Eif4ebp1*	-0.227	*-0.055*	*0.711*	0.056	0.689	-0.305	0.043	-0.295	0.026
ACLY	*Acly*	-0.339	*-1.654*	*0.002*	-1.324	0.007	-0.787	0.124	-1.310	0.003
AFX	*Foxo4*	-0.308	*-0.115*	*0.519*	-0.305	0.073	-0.185	0.301	-0.261	0.098
BAD	*Bad*	0.476	*-0.024*	*0.925*	0.089	0.711	0.240	0.339	0.415	0.062
c-FOS	*Fos*	0.121	*-1.152*	*0.104*	0.271	0.656	-0.193	0.770	0.217	0.705
c-JUN	*Jun*	-0.025	*-0.027*	*0.943*	0.834	0.017	-0.052	0.891	-0.014	0.966
c-RAF	*Raf1*	-0.299	*-0.165*	*0.253*	-0.215	0.116	-0.278	0.055	-0.247	0.052
C3G	*Rapgef1*	0.321	*0.178*	*0.169*	0.258	0.036	0.109	0.400	0.215	0.062
CASP9	*Casp9*	-0.257	*-0.279*	*0.109*	-0.157	0.336	-0.164	0.344	-0.471	0.002
CBL	*Cbl*	0.217	*-0.226*	*0.512*	0.034	0.916	0.044	0.897	0.089	0.766
CIP4	*Trip10*	0.217	*0.211*	*0.281*	0.383	0.037	0.256	0.190	0.281	0.107
eIF4E	*Eif4e*	-0.131	*-0.018*	*0.898*	-0.044	0.746	0.139	0.325	0.069	0.582
ELK-1	*Elk1*	-0.377	*-0.063*	*0.808*	0.045	0.852	-0.388	0.142	-0.412	0.077
FAK	*Ptk2*	-0.093	*0.133*	*0.357*	0.032	0.819	-0.038	0.797	0.021	0.873
FKHRL1	*Foxo3*	0.168	*-0.019*	*0.924*	-0.241	0.220	-0.120	0.562	0.015	0.933
FYN	*Fyn*	-0.297	*0.002*	*0.993*	0.118	0.519	0.012	0.953	-0.240	0.171
GAB1	*Gab1*	0.370	*0.234*	*0.149*	0.187	0.227	0.188	0.248	0.200	0.169
GLUT4	*Slc2a4*	0.168	*-1.636*	*0.013*	-0.688	0.244	0.257	0.671	-0.059	0.913
GRB10	*Grb10*	-0.142	*0.242*	*0.536*	0.444	0.231	-0.550	0.178	-0.432	0.224
GRB2	*Grb2*	-0.346	*-0.098*	*0.434*	0.003	0.982	-0.104	0.405	-0.123	0.266
IGF-1	*Igf1*	0.160	*0.284*	*0.223*	-0.005	0.983	0.321	0.169	0.053	0.800
IGF1R	*Igf1r*	0.293	*0.371*	*0.297*	0.573	0.088	0.257	0.476	0.693	0.029
INSR	*Insr*	0.507	*0.106*	*0.572*	-0.076	0.670	0.153	0.414	0.245	0.141
IRS1	*Irs1*	0.120	*-0.229*	*0.408*	-0.191	0.466	-0.129	0.639	0.412	0.089
JNK1	*Mapk8*	0.392	*0.183*	*0.239*	0.051	0.730	0.211	0.173	0.312	0.023
LAR	*Ptprf*	-0.623	*-0.293*	*0.176*	-0.325	0.113	-0.615	0.005	-0.594	0.002
LIPE	*Lipe*	-0.141	*-0.183*	*0.446*	-0.270	0.235	-0.265	0.271	-0.431	0.043
mTOR	*Mtor*	-0.119	*0.060*	*0.704*	0.159	0.289	0.043	0.787	-0.122	0.384
NCK	*Nck1*	-0.176	*-0.094*	*0.564*	-0.169	0.274	-0.205	0.212	-0.137	0.343
PDE3B	*Pde3b*	0.488	*0.236*	*0.276*	0.162	0.432	0.418	0.053	0.415	0.031
PDK1	*Pdpk1*	0.279	*0.092*	*0.564*	0.054	0.723	-0.018	0.910	0.220	0.120
PTEN	*Pten*	0.131	*0.001*	*0.995*	-0.097	0.595	0.069	0.717	0.041	0.807
PTP1B	*Ptpn1*	0.112	*0.040*	*0.799*	0.261	0.075	0.112	0.472	0.153	0.266
PXN	*Pxn*	0.248	*-0.126*	*0.367*	0.059	0.652	-0.112	0.424	0.054	0.661
RAPTOR	*Rptor*	-0.214	*-0.063*	*0.667*	-0.104	0.450	-0.167	0.254	-0.175	0.176
RAS GAP	*Rasa1*	0.001	*0.057*	*0.695*	-0.090	0.516	-0.034	0.818	-0.048	0.710
SGK	*Sgk1*	0.108	*0.275*	*0.330*	0.580	0.030	0.500	0.075	0.479	0.057
SHC	*Shc1*	-0.180	*-0.091*	*0.512*	0.026	0.845	-0.072	0.607	-0.258	0.036
SHP2	*Ptpn11*	0.124	*0.155*	*0.295*	0.114	0.418	0.045	0.764	0.179	0.173
SOCS3	*Socs3*	-0.221	*-0.655*	*0.148*	-0.046	0.913	-0.919	0.045	-0.677	0.086
SRF	*Srf*	-0.308	*-0.259*	*0.175*	-0.140	0.434	-0.432	0.026	-0.155	0.353
STAT3	*Stat3*	0.227	*-0.364*	*0.151*	0.348	0.142	0.043	0.862	0.271	0.223
STX4	*Stx4a*	-0.442	*-0.455*	*0.021*	-0.771	0.000	-0.361	0.065	-0.572	0.001
SYNIP	*Stxbp4*	-0.129	*-0.026*	*0.941*	-0.099	0.763	-0.295	0.406	-0.473	0.134

TC10	*Rhoq*	0.483	*0.240*	*0.171*	0.245	0.143	0.319	0.068	0.428	0.006
TSC1	*Tsc1*	0.205	*-0.028*	*0.846*	-0.272	0.047	0.014	0.922	0.046	0.713
TSC2	*Tsc2*	0.011	*0.078*	*0.581*	0.119	0.371	0.094	0.503	0.058	0.643
VAMP2	*Vamp2*	0.381	*-0.001*	*0.992*	-0.017	0.882	0.035	0.774	0.032	0.770

Table S2. Expression levels of genes involved in mTOR signaling pathway correlated with the increase in calorie restriction (CR) and their gene expression at each level of CR relative to 12h ad libitum intake.

			10CR		20CR		30CR		40CR	
Symbol	Gene Symbol	r	logFC	p	logFC	p	logFC	p	logFC	p
4EBP	*Eif4ebp1*	-0.227	-0.055	0.711	0.056	0.689	-0.305	0.043	-0.295	0.026
ATG13	*Atg13*	-0.003	-0.132	0.372	-0.241	0.088	-0.143	0.335	-0.097	0.458
DGKζ	*Dgkz*	0.070	0.067	0.655	0.249	0.080	0.041	0.784	0.090	0.499
eIF4B	*Eif4b*	-0.188	-0.026	0.882	-0.097	0.566	-0.054	0.761	-0.245	0.120
eIF4E	*Eif4e*	-0.131	-0.018	0.898	-0.044	0.746	0.139	0.325	0.069	0.582
FKBP1	*Fkbp1a*	-0.007	0.028	0.861	0.289	0.054	0.119	0.454	0.089	0.528
GBL	*Mlst8*	-0.440	0.244	0.367	0.231	0.369	-0.230	0.413	-0.196	0.428
HIF1α	*Hif1a*	0.060	0.008	0.954	-0.089	0.502	-0.113	0.421	-0.054	0.661
INSR	*Insr*	0.507	0.106	0.572	-0.076	0.670	0.153	0.414	0.245	0.141
IRS1	*Irs1*	0.120	-0.229	0.408	-0.191	0.466	-0.129	0.639	0.412	0.089
LKB1	*Stk11*	-0.353	0.037	0.776	-0.073	0.558	-0.158	0.232	-0.165	0.159
mTOR	*Mtor*	-0.119	0.060	0.704	0.159	0.289	0.043	0.787	-0.122	0.384
p70S6K	*Rps6kb1*	0.112	0.098	0.566	-0.043	0.791	0.022	0.899	0.131	0.384
PDK1	*Pdpk1*	0.279	0.092	0.564	0.054	0.723	-0.018	0.910	0.220	0.120
PKCα	*Prkca*	0.269	0.134	0.509	0.079	0.681	0.151	0.455	0.296	0.099
PRAS40	*Akt1s1*	-0.256	-0.189	0.199	-0.139	0.315	-0.208	0.159	-0.257	0.048
RAC	*Rac1*	-0.077	-0.032	0.801	-0.003	0.981	-0.037	0.773	-0.059	0.604
RAPTOR	*Rptor*	-0.214	-0.063	0.667	-0.104	0.450	-0.167	0.254	-0.175	0.176
REDD1	*Ddit4*	0.446	0.176	0.752	0.840	0.106	0.954	0.078	1.777	0.000
RHEB	*Rheb*	0.222	0.117	0.447	0.184	0.205	0.092	0.548	0.222	0.103
RICTOR	*Rictor*	0.328	0.312	0.161	0.231	0.276	0.270	0.226	0.332	0.094
RPS6	*Rps6*	0.487	-0.032	0.858	0.035	0.839	0.163	0.364	0.268	0.093
SIN1	*Mapkap1*	-0.318	-0.018	0.889	-0.119	0.345	-0.118	0.375	-0.131	0.267
TSC1	*Tsc1*	0.205	-0.028	0.846	-0.272	0.047	0.014	0.922	0.046	0.713
TSC2	*Tsc2*	0.011	0.078	0.581	0.119	0.371	0.094	0.503	0.058	0.643
ULK1	*Ulk1*	-0.040	-0.384	0.029	-0.214	0.197	-0.290	0.098	-0.103	0.504

Table S3. Expression levels of genes involved in NF-κB signaling pathway correlated with the increase in calorie restriction (CR) and their gene expression at each level of CR relative to 12h ad libitum intake.

			10CR		20CR		30CR		40CR	
Symbol	Gene Symbol	r	logFC	p	logFC	p	logFC	p	logFC	p
β-TrCP	*Btrc*	-0.112	-0.003	0.986	0.009	0.958	0.020	0.913	-0.157	0.337
A20	*Tnfaip3*	-0.100	0.218	0.582	0.351	0.349	-0.399	0.334	-0.311	0.389
ABIN-1	*Tnip1*	-0.219	-0.115	0.764	0.330	0.358	-0.569	0.146	-0.645	0.059
Bcl10	*Bcl10*	0.106	-0.238	0.314	0.110	0.616	0.101	0.663	0.007	0.975
BIMP1	*Card10*	0.062	0.207	0.354	0.318	0.132	0.231	0.302	0.120	0.548
c-RAF	*Raf1*	-0.299	-0.165	0.253	-0.215	0.116	-0.278	0.055	-0.247	0.052

CARD11	*Card11*	-0.294	-0.096	0.883	-0.445	0.479	-0.963	0.166	-1.573	0.013
Caspase8	*Casp8*	0.075	0.093	0.508	0.039	0.770	0.047	0.737	-0.072	0.568
CCR5	*Ccr5*	-0.087	0.160	0.608	0.536	0.065	0.350	0.258	0.112	0.687
CD4	*Cd4*	-0.550	-0.161	0.797	-0.779	0.204	-2.064	0.005	-1.509	0.011
Cot	*Map3k8*	0.125	-0.422	0.256	-0.131	0.703	-0.368	0.321	-0.050	0.877
FADD	*Fadd*	-0.480	-0.293	0.095	-0.301	0.070	-0.415	0.019	-0.565	0.000
GSK-3β	*Gsk3b*	0.117	0.144	0.273	0.063	0.614	0.006	0.966	0.095	0.416
HVEM	*Tnfrsf14*	-0.030	0.162	0.622	0.339	0.270	0.186	0.572	-0.191	0.528
IKKα	*Chuk*	0.065	0.152	0.458	-0.183	0.349	0.035	0.865	-0.021	0.908
IKKβ	*Ikbkb*	-0.137	-0.011	0.926	-0.068	0.535	-0.054	0.646	-0.124	0.230
IKKγ	*Ikbkg*	-0.099	0.213	0.163	0.202	0.165	-0.021	0.892	0.072	0.599
IRAK-M	*Irak3*	0.031	-0.275	0.389	-0.126	0.671	-0.430	0.187	0.047	0.865
JNK1	*Mapk8*	0.392	0.183	0.239	0.051	0.730	0.211	0.173	0.312	0.023
LTBR	*Ltbr*	-0.028	-0.067	0.630	0.041	0.755	-0.107	0.442	-0.024	0.844
MALT1	*Malt1*	-0.026	0.070	0.704	-0.135	0.446	-0.024	0.899	-0.040	0.808
MEKK1	*Map3k1*	-0.017	0.233	0.317	0.167	0.452	-0.016	0.946	0.145	0.486
MYD88	*Myd88*	0.040	-0.245	0.162	-0.095	0.564	-0.174	0.318	0.009	0.954
NAK	*Tbk1*	0.311	-0.048	0.771	-0.089	0.570	-0.024	0.883	0.254	0.079
NAP1	*Azi2*	0.566	0.166	0.283	-0.040	0.784	0.281	0.067	0.236	0.085
NF-κB1 p50	*Nfkb1*	-0.133	0.105	0.571	-0.092	0.605	-0.161	0.396	-0.100	0.548
NF-κB2 p100	*Nfkb2*	-0.234	0.203	0.592	0.479	0.182	-0.394	0.313	-0.734	0.034
NGF	*Ngf*	-0.225	0.430	0.558	0.679	0.333	-0.142	0.850	-0.245	0.711
NIK	*Map3k14*	0.156	0.511	0.241	1.164	0.004	0.402	0.361	0.667	0.088
p65/RelA	*Rela*	-0.300	-0.150	0.371	-0.134	0.396	-0.409	0.016	-0.292	0.049
PELI1	*Peli1*	0.206	0.022	0.896	-0.185	0.247	-0.106	0.527	0.025	0.866
PKCζ	*Prkcz*	-0.322	-0.132	0.538	-0.358	0.081	-0.368	0.089	-0.566	0.003
PKR	*Eif2ak2*	-0.007	0.058	0.719	-0.039	0.801	0.057	0.727	0.084	0.559
PLCγ2	*Plcg2*	-0.289	0.043	0.917	0.196	0.619	-0.318	0.454	-1.239	0.001
RelB	*Relb*	-0.255	0.435	0.434	0.567	0.283	-0.477	0.414	-1.635	0.003
RIP	*Ripk1*	0.125	0.025	0.864	0.012	0.927	-0.139	0.336	0.063	0.619
TAB1	*Tab1*	0.100	0.458	0.179	0.301	0.358	0.341	0.323	0.388	0.207
TANK	*Tank*	-0.150	0.138	0.360	-0.049	0.734	-0.178	0.250	0.035	0.793
TBP	*Tbp*	0.368	0.146	0.476	0.122	0.531	0.052	0.803	0.443	0.014
TGF-α	*Tgfa*	-0.169	-0.020	0.907	-0.033	0.844	-0.340	0.057	-0.297	0.058
TIRAP	*Tirap*	-0.481	-0.091	0.608	-0.187	0.267	-0.044	0.804	-0.222	0.157
TRADD	*Tradd*	-0.114	-0.526	0.016	-0.135	0.494	-0.100	0.631	-0.240	0.198
TRAF2	*Traf2*	-0.004	0.326	0.163	0.400	0.071	0.112	0.638	0.206	0.330
TRAF6	*Traf6*	0.420	0.135	0.414	0.082	0.601	0.150	0.365	0.383	0.009
TTRAP	*Tdp2*	0.498	0.012	0.949	0.050	0.787	0.382	0.047	0.288	0.094
UBE2N	*Ube2n*	-0.386	-0.143	0.276	-0.157	0.209	-0.223	0.092	-0.120	0.302
UBE2V1	*Ube2v1*	-0.301	-0.096	0.446	0.027	0.818	-0.069	0.584	-0.080	0.472
Zap70	*Zap70*	-0.169	0.242	0.441	0.624	0.034	0.314	0.316	-0.250	0.381

Table S4. Expression levels of genes involved in sirtuin signaling pathway correlated with the increase in calorie restriction (CR) and their gene expression at each level of CR relative to 12h ad libitum intake.

			10CR		20CR		30CR		40CR	
**Symbol**	**Gene symbol**	**r**	**logFC**	**p**	**logFC**	**p**	**logFC**	**p**	**logFC**	**p**
ACADL	*Acadl*	0.763	0.111	0.612	0.263	0.208	0.468	0.032	0.557	0.005
ARNTL	*Arntl*	-0.105	-1.581	0.038	-1.588	0.026	-1.808	0.020	0.261	0.674

BIRC5	*Birc5*	-0.039	0.321	0.580	1.432	0.008	-0.403	0.507	0.053	0.920
CDKN1A	*Cdkn1a*	0.055	0.775	0.288	1.702	0.016	-0.253	0.736	1.225	0.066
CPS1	*Cps1*	0.476	-0.279	0.234	-0.222	0.317	0.030	0.899	0.177	0.392
CRTC2	*Crtc2*	0.626	0.135	0.439	0.130	0.432	0.220	0.204	0.463	0.003
CTNNB1	*Ctnnb1*	-0.363	-0.100	0.550	-0.083	0.601	-0.220	0.190	-0.268	0.069
CYC1	*Cyc1*	0.617	-0.086	0.611	0.020	0.898	0.157	0.349	0.309	0.039
CYCS	*Cycs*	0.572	0.064	0.782	-0.084	0.702	0.231	0.313	0.523	0.011
E2F1	*E2f1*	-0.296	0.182	0.629	0.681	0.050	-0.128	0.741	-0.441	0.210
EPAS1	*Epas1*	0.343	-0.396	0.183	-0.125	0.655	0.108	0.714	0.108	0.678
FOXO1	*Foxo1*	0.603	0.236	0.302	0.322	0.139	0.382	0.094	0.681	0.001
FOXO3	*Foxo3*	0.168	-0.019	0.924	-0.241	0.220	-0.120	0.562	0.015	0.933
FOXO4	*Foxo4*	-0.308	-0.115	0.519	-0.305	0.073	-0.185	0.301	-0.261	0.098
GLUD1	*Glud1*	0.655	-0.123	0.559	0.047	0.815	0.230	0.271	0.460	0.014
HIF1A	*Hif1a*	0.060	0.008	0.954	-0.089	0.502	-0.113	0.421	-0.054	0.661
HNF4A	*Hnf4a*	0.338	-0.104	0.665	0.075	0.744	-0.060	0.802	0.316	0.139
HSF1	*Hsf1*	0.015	-0.047	0.744	-0.059	0.666	-0.040	0.782	0.012	0.925
IDE	*Ide*	-0.662	-0.125	0.442	-0.345	0.026	-0.422	0.010	-0.371	0.010
IDH2	*Idh2*	0.556	0.443	0.099	0.740	0.004	0.818	0.002	0.850	<0.001
MRPL10	*Mrpl10*	-0.223	-0.127	0.302	0.007	0.949	-0.188	0.131	-0.035	0.747
NDUFA9	*Ndufa9*	0.506	-0.136	0.385	-0.142	0.340	0.097	0.535	0.240	0.082
NFKB1	*Nfkb1*	-0.133	0.105	0.571	-0.092	0.605	-0.161	0.396	-0.100	0.548
NFKB2	*Nfkb2*	-0.234	0.203	0.592	0.479	0.182	-0.394	0.313	-0.734	0.034
NR1H2	*Nr1h2*	0.381	-0.140	0.341	0.037	0.787	0.101	0.485	0.232	0.069
NR1H3	*Nr1h3*	-0.540	-0.134	0.458	-0.134	0.432	-0.152	0.397	-0.344	0.030
PARP1	*Parp1*	-0.363	-0.176	0.288	-0.179	0.252	-0.208	0.209	-0.418	0.004
PER2	*Per2*	0.724	0.671	0.039	0.828	0.008	1.028	0.002	0.950	0.001
PIP5K1A	*Pip5k1a*	0.105	-0.069	0.723	0.277	0.121	-0.003	0.988	-0.009	0.960
PIP5K1C	*Pip5k1c*	-0.120	-0.027	0.840	0.135	0.282	-0.001	0.995	0.049	0.676
PPARA	*Ppara*	0.257	-0.004	0.987	-0.055	0.811	0.114	0.637	-0.037	0.864
PPARG	*Pparg*	-0.012	0.249	0.483	0.534	0.113	0.024	0.947	-0.111	0.729
PPARGC1A	*Ppargc1a*	0.806	-0.172	0.624	0.249	0.452	0.623	0.071	1.064	0.001
PPID	*Ppid*	-0.654	-0.037	0.856	-0.242	0.208	-0.380	0.063	-0.319	0.074
RARA	*Rara*	-0.358	0.048	0.765	0.051	0.739	-0.203	0.217	-0.363	0.013
RARB	*Rarb*	0.370	0.166	0.594	0.802	0.005	0.631	0.037	0.646	0.017
RB1	*Rb1*	-0.315	0.052	0.757	-0.338	0.039	-0.251	0.146	-0.217	0.153
RBL1	*Rbl1*	-0.021	0.645	0.161	0.934	0.032	0.086	0.858	-0.011	0.980
RBL2	*Rbl2*	0.562	0.004	0.983	-0.245	0.127	0.051	0.762	0.252	0.090
RELA	*Rela*	-0.300	-0.150	0.371	-0.134	0.396	-0.409	0.016	-0.292	0.049
SDHA	*Sdha*	0.185	-0.084	0.658	-0.239	0.185	0.027	0.886	0.013	0.937
SDHB	*Sdhb*	0.462	0.127	0.474	-0.026	0.876	0.195	0.271	0.193	0.220
SIRT1	*Sirt1*	0.214	0.016	0.938	0.066	0.732	-0.119	0.560	0.299	0.092
SIRT2	*Sirt2*	-0.080	-0.038	0.754	-0.037	0.749	-0.131	0.282	-0.098	0.361
SIRT3	*Sirt3*	-0.032	0.028	0.890	-0.262	0.174	-0.003	0.988	-0.233	0.192
SIRT4	*Sirt4*	-0.653	0.037	0.882	-0.299	0.216	-0.638	0.017	-0.499	0.030
SIRT5	*Sirt5*	-0.297	-0.066	0.770	-0.172	0.422	-0.033	0.884	-0.523	0.010
SIRT6	*Sirt6*	0.051	0.203	0.561	0.142	0.669	-0.145	0.687	0.035	0.911
SIRT7	*Sirt7*	-0.501	-0.184	0.248	-0.317	0.037	-0.286	0.074	-0.304	0.031
SLC25A5	*Slc25a5*	0.732	0.046	0.803	0.165	0.344	0.368	0.044	0.478	0.003
SMAD7	*Smad7*	0.052	0.229	0.338	0.123	0.588	0.431	0.069	-0.247	0.254
SREBF1	*Srebf1*	-0.463	-0.260	0.435	-0.102	0.745	-0.343	0.304	-0.914	0.002
SREBF2	*Srebf2*	0.257	-0.236	0.226	0.115	0.529	0.035	0.855	0.094	0.583
TLE1	*Tle1*	-0.153	-0.255	0.141	-0.284	0.083	-0.443	0.011	-0.049	0.746
TP53	*Trp53*	-0.159	0.136	0.551	0.326	0.131	-0.272	0.245	-0.076	0.711

TSC2	*Tsc2*	0.011	0.078	0.581	0.119	0.371	0.094	0.503	0.058	0.643
TUBA1A	*Tuba1a*	-0.226	-0.288	0.545	0.102	0.818	-0.756	0.124	-0.799	0.062
TUBA1B	*Tuba1b*	-0.307	-0.171	0.565	0.169	0.541	-0.311	0.301	-0.530	0.047
TUBA1C	*Tuba1c*	-0.377	-0.283	0.481	0.108	0.774	-0.693	0.090	-0.688	0.053
TUBA4A	*Tuba4a*	-0.595	-0.502	0.077	-0.665	0.014	-0.803	0.005	-0.597	0.016
TUBA8	*Tuba8*	0.525	1.004	0.123	1.542	0.014	1.050	0.108	2.228	<0.001
UCP2	*Ucp2*	-0.133	0.204	0.479	0.582	0.033	0.177	0.540	-0.190	0.458
WRN	*Wrn*	-0.057	0.014	0.930	-0.190	0.222	-0.129	0.430	-0.135	0.351
XRCC6	*Xrcc6*	-0.321	-0.083	0.677	0.210	0.256	-0.045	0.820	-0.238	0.179

Table S5. Expression levels of genes involved in oxidative stress signaling pathway correlated with the increase in calorie restriction (CR) and their gene expression at each level of CR relative to 12h ad libitum intake.

			10CR		20CR		30CR		40CR	
**Symbol**	**Gene Symbol**	**r**	**logFC**	**p**	**logFC**	**p**	**logFC**	**p**	**logFC**	**p**
53BP1	*Trp53bp1*	-0.455	0.142	0.346	-0.124	0.392	-0.160	0.297	-0.182	0.181
AKR	*Akr1a1*	-0.214	-0.013	0.939	0.062	0.704	-0.016	0.926	-0.083	0.587
AKT	*Akt1*	0.072	0.049	0.710	0.258	0.038	0.109	0.405	0.046	0.695
AOX1	*Aox1*	-0.316	0.156	0.476	0.159	0.446	-0.046	0.835	-0.185	0.343
ARD1	*Naa10*	0.052	-0.251	0.082	-0.064	0.633	-0.025	0.861	0.004	0.977
ARNT	*Arnt*	0.457	0.085	0.566	0.091	0.519	0.273	0.063	0.201	0.126
ASK1	*Map3k5*	0.525	0.195	0.382	0.019	0.928	0.152	0.494	0.294	0.137
ATF2	*Atf2*	0.414	0.014	0.922	-0.053	0.690	0.046	0.745	0.208	0.093
ATF4	*Atf4*	-0.141	-0.061	0.700	-0.055	0.714	-0.034	0.830	-0.161	0.253
ATF6	*Atf6*	-0.227	-0.058	0.724	-0.022	0.889	-0.318	0.055	-0.090	0.538
ATM	*Atm*	0.005	0.105	0.576	-0.050	0.780	0.011	0.954	-0.078	0.646
BACH1	*Bach1*	0.070	0.365	0.051	0.088	0.625	0.096	0.610	-0.094	0.573
BID	*Bid*	-0.364	-0.040	0.835	-0.201	0.275	-0.184	0.345	-0.408	0.019
BIP	*Hspa5*	-0.431	-0.164	0.571	-0.197	0.473	-0.519	0.076	-0.500	0.050
BLM	*Blm*	-0.066	-0.193	0.591	-0.702	0.044	-0.239	0.507	-0.460	0.150
BRCA1	*Brca1*	0.463	0.008	0.985	0.734	0.055	0.903	0.024	1.072	0.003
c-Abl	*Abl1*	0.245	0.189	0.215	0.260	0.071	0.246	0.105	0.122	0.371
c-Jun	*Jun*	-0.025	-0.027	0.943	0.834	0.017	-0.052	0.891	-0.014	0.966
c-MAF	*Maf*	0.259	0.052	0.748	0.210	0.175	0.236	0.146	0.176	0.223
c-RAF	*Raf1*	-0.299	-0.165	0.253	-0.215	0.116	-0.278	0.055	-0.247	0.052
CALR	*Calr*	-0.616	-0.562	0.065	-0.444	0.122	-0.902	0.003	-0.638	0.016
CASP3	*Casp3*	-0.315	-0.439	0.038	-0.312	0.115	-0.059	0.774	-0.472	0.011
CASP7	*Casp7*	0.158	-0.014	0.941	-0.025	0.887	0.268	0.142	-0.086	0.600
CASP9	*Casp9*	-0.257	-0.279	0.109	-0.157	0.336	-0.164	0.344	-0.471	0.002
CAT	*Cat*	-0.805	-0.091	0.700	-0.470	0.039	-0.487	0.043	-0.668	0.001
CBP	*Crebbp*	0.395	0.196	0.375	0.185	0.379	0.138	0.533	0.399	0.042
CBR1	*Cbr1*	0.712	0.505	0.221	1.316	0.001	1.314	0.001	1.858	0.000
CCT7	*Cct7*	0.068	-0.152	0.253	0.049	0.697	-0.010	0.937	0.005	0.968
CDC25A	*Cdc25a*	0.175	0.154	0.598	0.426	0.116	0.273	0.348	0.376	0.144
CDK1	*Cdk1*	-0.132	0.564	0.430	1.514	0.025	0.445	0.538	-0.742	0.267
CDK2	*Cdk2*	0.027	0.145	0.420	-0.176	0.315	0.068	0.709	-0.078	0.631
Chk2	*Chek2*	-0.238	0.188	0.623	0.426	0.234	0.330	0.386	-0.226	0.524
CHOP	*Ddit3*	-0.200	0.635	0.174	0.784	0.079	0.146	0.759	-0.136	0.749
CLPP	*Clpp*	-0.026	-0.128	0.359	-0.074	0.574	-0.092	0.511	-0.004	0.977
CSF2Rα	*Csf2ra*	-0.123	0.121	0.738	0.439	0.197	-0.017	0.962	-0.735	0.026
CSF2Rβ	*Csf2rb2*	-0.032	0.382	0.327	0.706	0.056	0.191	0.554	-0.029	0.921

CUL2	*Cul2*	-0.524	0.027	0.848	-0.253	0.061	-0.094	0.508	-0.186	0.137
EIF2S1	*Eif2s1*	-0.195	0.050	0.740	0.101	0.480	-0.117	0.440	-0.105	0.433
eIF4E	*Eif4e*	-0.131	-0.018	0.898	-0.044	0.746	0.139	0.325	0.069	0.582
elF2α	*Eif2a*	0.502	0.059	0.628	0.019	0.873	0.161	0.188	0.185	0.089
elF2γ	*Eif2s3x*	0.426	0.109	0.460	-0.003	0.985	0.159	0.280	0.253	0.054
elF4C	*Eif1a*	0.038	-0.028	0.906	0.159	0.483	-0.019	0.938	0.221	0.296
ELK-1	*Elk1*	-0.377	-0.063	0.808	0.045	0.852	-0.388	0.142	-0.412	0.077
EPHX1	*Ephx1*	0.042	0.282	0.421	0.846	0.011	0.418	0.232	0.633	0.044
ERK5	*Mapk7*	-0.087	0.035	0.909	0.106	0.709	-0.127	0.682	-0.082	0.765
ERP29	*Erp29*	-0.508	-0.227	0.173	-0.084	0.593	-0.293	0.080	-0.313	0.033
FKBP5	*Fkbp5*	0.780	0.438	0.411	1.267	0.013	1.575	0.003	2.180	0.000
FMO1	*Fmo1*	0.818	0.439	0.149	0.724	0.012	1.206	0.000	1.099	0.000
FTH1	*Fth1*	0.532	0.184	0.319	0.283	0.108	0.326	0.077	0.409	0.013
GADD34	*Ppp1r15a*	0.191	0.049	0.799	0.170	0.348	-0.004	0.984	0.139	0.416
GCLC	*Gclc*	-0.388	0.246	0.368	0.017	0.948	-0.571	0.041	-0.504	0.038
GCLM	*Gclm*	0.003	0.221	0.152	0.332	0.024	0.131	0.396	0.160	0.245
GCN2	*Eif2ak4*	0.387	-0.003	0.989	0.084	0.635	0.139	0.454	0.300	0.067
gp91	*Cybb*	-0.071	0.217	0.534	0.732	0.027	0.021	0.951	-0.483	0.120
GRB2	*Grb2*	-0.346	-0.098	0.434	0.003	0.982	-0.104	0.405	-0.123	0.266
GRP94	*Hsp90b1*	-0.486	-0.287	0.160	-0.337	0.082	-0.370	0.071	-0.302	0.092
GSK3β	*Gsk3b*	0.117	0.144	0.273	0.063	0.614	0.006	0.966	0.095	0.416
GSR	*Gsr*	-0.153	0.054	0.710	0.121	0.376	-0.187	0.198	-0.034	0.788
H2AX	*H2afx*	-0.111	0.212	0.396	0.289	0.220	-0.184	0.479	0.144	0.523
HERPUD1	*Herpud1*	0.184	-0.120	0.645	0.004	0.987	-0.025	0.922	0.216	0.347
HIF1α	*Hif1a*	0.060	0.008	0.954	-0.089	0.502	-0.113	0.421	-0.054	0.661
HIP2	*Ube2k*	0.058	-0.050	0.678	-0.039	0.730	-0.006	0.963	0.124	0.245
HO-1	*Hmox1*	0.117	-0.006	0.985	0.739	0.020	-0.080	0.813	0.241	0.418
HP1	*Cbx5*	0.205	0.255	0.067	0.183	0.167	0.177	0.204	0.062	0.620
HRI	*Eif2ak1*	0.151	-0.016	0.903	-0.161	0.192	-0.118	0.363	-0.002	0.989
HSP90	*Hsp90aa1*	-0.423	0.055	0.820	0.124	0.588	-0.203	0.404	-0.150	0.483
IκBα	*Nfkbia*	0.575	0.116	0.557	0.074	0.695	0.160	0.418	0.462	0.008
ICAM1	*Icam1*	-0.056	-0.036	0.914	0.411	0.194	0.081	0.809	-0.085	0.775
ICSBP	*Irf8*	-0.080	0.320	0.324	0.472	0.125	0.093	0.776	-0.458	0.121
INSR	*Insr*	0.507	0.106	0.572	-0.076	0.670	0.153	0.414	0.245	0.141
IRE1	*Ern1*	-0.184	-0.033	0.895	-0.344	0.154	-0.466	0.069	-0.009	0.969
IRF-1	*Irf1*	-0.132	-0.040	0.851	-0.090	0.656	-0.261	0.225	-0.208	0.269
JAB1	*Cops5*	0.151	-0.080	0.547	-0.069	0.583	0.044	0.739	-0.008	0.945
JAK2	*Jak2*	-0.138	0.054	0.707	-0.132	0.337	-0.130	0.368	-0.177	0.167
JIK	*Taok3*	0.580	0.045	0.804	0.040	0.813	0.101	0.571	0.349	0.028
JNK1	*Mapk8*	0.392	0.183	0.239	0.051	0.730	0.211	0.173	0.312	0.023
KAP-1	*Trim28*	0.142	-0.072	0.661	0.024	0.880	0.007	0.965	0.005	0.974
MDC1	*Mdc1*	0.099	-0.010	0.960	0.079	0.672	0.082	0.680	0.117	0.506
MDM2	*Mdm2*	0.001	0.116	0.521	0.217	0.203	0.048	0.791	0.120	0.456
MDMX	*Mdm4*	0.309	0.298	0.281	0.279	0.289	0.337	0.222	0.311	0.207
MEK1	*Map2k1*	-0.493	-0.174	0.283	-0.122	0.426	-0.182	0.262	-0.359	0.012
MEK5	*Map2k5*	0.099	-0.098	0.545	0.004	0.978	-0.200	0.220	-0.058	0.687
MEKK	*Map3k1*	-0.017	0.233	0.317	0.167	0.452	-0.016	0.946	0.145	0.486
MRE11	*Mre11a*	0.072	0.142	0.625	0.123	0.657	-0.236	0.435	0.075	0.773
MRP1	*Abcc1*	-0.030	0.317	0.441	0.360	0.357	-0.148	0.727	-0.369	0.329
MRP2	*Abcc2*	0.241	0.209	0.333	0.190	0.355	0.132	0.543	0.139	0.470
MRP4	*Abcc4*	0.073	1.657	0.007	2.157	0.000	0.988	0.108	1.217	0.030
NBS1	*Nbn*	0.141	0.014	0.925	-0.089	0.534	-0.079	0.604	0.024	0.857
NRF2	*Nfe2l2*	-0.139	-0.035	0.828	-0.105	0.491	-0.146	0.365	-0.057	0.687

NRPB	*Enc1*	0.455	0.275	0.558	1.004	0.024	0.988	0.033	1.406	0.001
p21CIP1	*Cdkn1a*	0.055	0.775	0.288	1.702	0.016	-0.253	0.736	1.225	0.066
p22phox	*Cyba*	-0.101	0.115	0.767	0.647	0.075	0.062	0.874	-0.394	0.258
p38MAPK	*Mapk14*	0.614	0.219	0.157	0.243	0.099	0.306	0.048	0.436	0.002
p40phox	*Ncf4*	-0.231	-0.210	0.679	0.161	0.733	-0.169	0.739	-1.054	0.025
p47phox	*Ncf1*	-0.150	0.301	0.338	0.621	0.036	-0.082	0.799	-0.444	0.123
p53	*Trp53*	-0.159	0.136	0.551	0.326	0.131	-0.272	0.245	-0.076	0.711
P58IPK	*Dnajc3*	-0.105	-0.070	0.703	-0.144	0.409	-0.119	0.518	0.083	0.609
p67phox	*Ncf2*	-0.112	-0.069	0.857	0.271	0.447	-0.019	0.960	-0.427	0.210
PABP	*Pabpc1*	-0.272	0.045	0.775	0.011	0.944	-0.191	0.230	-0.180	0.200
PAI-1	*Serpine1*	0.039	-0.037	0.954	0.875	0.140	0.254	0.687	-0.365	0.521
PDK1	*Pdpk1*	0.279	0.092	0.564	0.054	0.723	-0.018	0.910	0.220	0.120
PERK	*Eif2ak3*	-0.206	0.109	0.663	-0.187	0.438	-0.587	0.024	-0.329	0.145
PKCβ	*Prkcb*	-0.177	-0.169	0.508	0.129	0.589	-0.223	0.385	-0.648	0.005
PKR	*Eif2ak2*	-0.007	0.058	0.719	-0.039	0.801	0.057	0.727	0.084	0.559
PMF-1	*Pmf1*	0.453	-0.372	0.063	-0.128	0.491	0.074	0.702	0.211	0.221
PPARα	*Ppara*	0.257	-0.004	0.987	-0.055	0.811	0.114	0.637	-0.037	0.864
PPIB	*Ppib*	-0.602	-0.394	0.099	-0.258	0.252	-0.507	0.034	-0.526	0.012
PRDX1	*Prdx1*	-0.201	-0.130	0.457	-0.079	0.632	-0.058	0.738	-0.078	0.610
Rac1	*Rac1*	-0.077	-0.032	0.801	-0.003	0.981	-0.037	0.773	-0.059	0.604
Rad50	*Rad50*	0.066	0.028	0.870	0.108	0.508	0.063	0.714	-0.009	0.956
RAD9	*Rad9a*	0.112	0.093	0.512	0.175	0.191	0.048	0.735	0.033	0.792
REF1	*Apex1*	-0.363	-0.469	0.021	-0.188	0.323	-0.298	0.140	-0.469	0.009
SHC	*Shc1*	-0.180	-0.091	0.512	0.026	0.845	-0.072	0.607	-0.258	0.036
SIRPα	*Sirpa*	-0.184	0.119	0.738	0.451	0.180	-0.253	0.481	-0.597	0.059
SP1	*Sp1*	-0.145	-0.081	0.528	-0.179	0.146	-0.215	0.100	-0.138	0.228
SQSTM1	*Sqstm1*	-0.287	0.000	0.999	0.200	0.481	-0.483	0.110	-0.475	0.072
SR-BI	*Scarb1*	0.155	-0.154	0.397	0.015	0.930	-0.146	0.422	0.125	0.438
SRC-1	*Ncoa1*	0.269	-0.018	0.903	-0.090	0.507	0.104	0.465	0.194	0.123
STAT1	*Stat1*	-0.217	0.044	0.866	0.120	0.631	-0.417	0.116	-0.336	0.148
STIP1	*Stip1*	-0.520	-0.131	0.496	-0.084	0.646	-0.361	0.064	-0.289	0.089
TCEB1	*Tceb1*	-0.252	0.119	0.519	0.005	0.978	0.076	0.682	-0.016	0.922
TDP1	*Tdp1*	-0.089	-0.083	0.827	0.200	0.569	0.005	0.990	-0.327	0.339
TLR4	*Tlr4*	-0.006	-0.007	0.979	0.146	0.576	0.044	0.875	0.005	0.986
TPA	*Plat*	-0.148	0.369	0.658	0.767	0.334	-1.024	0.248	-1.440	0.064
TRAF2	*Traf2*	-0.004	0.326	0.163	0.400	0.071	0.112	0.638	0.206	0.330
TRXR1	*Txnrd1*	-0.057	0.190	0.296	0.174	0.314	-0.053	0.773	0.134	0.405
TXN	*Txn1*	-0.298	-0.127	0.418	-0.351	0.019	-0.176	0.265	-0.196	0.157
UB2R1	*Cdc34*	-0.111	0.082	0.607	0.241	0.108	-0.105	0.512	0.053	0.707
UbcM2	*Ube2e3*	0.477	-0.026	0.855	0.063	0.640	0.154	0.275	0.241	0.054
USP14	*Usp14*	-0.191	-0.044	0.732	-0.114	0.352	-0.041	0.750	-0.039	0.734
VCAM1	*Vcam1*	0.456	0.122	0.711	0.847	0.007	0.542	0.097	0.639	0.029
VCP	*Vcp*	-0.549	-0.369	0.088	-0.321	0.116	-0.538	0.013	-0.542	0.004
VHL	*Vhl*	-0.256	0.052	0.835	0.116	0.621	-0.004	0.986	-0.045	0.838
XBP1	*Xbp1*	-0.571	-0.085	0.732	-0.283	0.230	-0.429	0.087	-0.659	0.003

Table S6. Expression levels of genes involved in reproduction signaling pathway correlated with the increase in calorie restriction (CR) and their gene expression at each level of CR relative to 12h ad libitum intake.

Symbol	Gene Symbol	r	10CR logFC	10CR p	20CR logFC	20CR p	30CR logFC	30CR p	40CR logFC	40CR p
β-catenin	*Ctnnb1*	-0.363	-0.100	0.550	-0.083	0.601	-0.220	0.190	-0.268	0.069
ACOX1	*Acox1*	0.368	0.052	0.793	0.115	0.541	0.242	0.221	0.079	0.653
AHR	*Ahr*	0.468	-0.012	0.957	0.117	0.564	0.293	0.166	0.422	0.025
AKT1	*Akt1*	0.072	0.049	0.710	0.258	0.038	0.109	0.405	0.046	0.695
Apaf1	*Apaf1*	0.020	0.141	0.693	0.775	0.018	0.373	0.290	0.037	0.908
ASPH	*Asph*	-0.207	0.026	0.858	-0.093	0.499	0.155	0.287	-0.043	0.737
ATF2	*Atf2*	0.414	0.014	0.922	-0.053	0.690	0.046	0.745	0.208	0.093
ATM	*Atm*	0.005	0.105	0.576	-0.050	0.780	0.011	0.954	-0.078	0.646
ATR	*Atr*	0.422	0.237	0.206	0.302	0.089	0.340	0.068	0.422	0.011
AXIN	*Axin1*	0.117	-0.146	0.373	-0.029	0.851	-0.154	0.349	0.088	0.540
BAX	*Bax*	-0.138	-0.095	0.696	0.118	0.604	-0.205	0.403	-0.219	0.312
Bcl-2	*Bcl2*	-0.221	0.665	0.077	0.396	0.274	-0.449	0.265	-0.401	0.256
Bcl-xL	*Bcl2l1*	-0.005	0.134	0.473	0.224	0.205	-0.077	0.684	0.065	0.695
Brca1	*Brca1*	0.463	0.008	0.985	0.734	0.055	0.903	0.024	1.072	0.003
c-Jun	*Jun*	-0.025	-0.027	0.943	0.834	0.017	-0.052	0.891	-0.014	0.966
c-Raf	*Raf1*	-0.299	-0.165	0.253	-0.215	0.116	-0.278	0.055	-0.247	0.052
CABC1	*Adck3*	-0.158	-0.780	0.025	-0.975	0.003	-0.615	0.075	-0.145	0.628
Caspase 6	*Casp6*	0.263	-0.210	0.243	-0.154	0.366	0.177	0.315	0.024	0.881
CDK2	*Cdk2*	0.027	0.145	0.420	-0.176	0.315	0.068	0.709	-0.078	0.631
CDK4	*Cdk4*	-0.195	-0.021	0.904	0.111	0.508	-0.094	0.595	-0.288	0.068
CEBPA	*Cebpa*	-0.650	-0.363	0.155	-0.441	0.069	-0.512	0.046	-0.817	0.000
CGN	*Cgn*	-0.260	-0.382	0.041	-0.284	0.105	-0.443	0.018	-0.246	0.133
Chk2	*Chek2*	-0.238	0.188	0.623	0.426	0.234	0.330	0.386	-0.226	0.524
CK1δ	*Csnk1d*	0.154	-0.057	0.648	0.067	0.570	-0.100	0.426	0.148	0.179
CTNNγ	*Jup*	0.163	-0.203	0.247	0.307	0.062	0.136	0.433	0.171	0.266
CyclinG	*Ccng1*	-0.121	0.068	0.665	0.035	0.813	0.079	0.616	-0.107	0.447
CyclinK	*Ccnk*	0.409	-0.082	0.576	0.076	0.579	0.004	0.976	0.138	0.282
Cyclin D1	*Ccnd1*	0.103	0.588	0.224	1.520	0.001	1.324	0.006	0.226	0.601
Cyclin D2	*Ccnd2*	-	0.264	0.353	0.128	0.637	-	0.555	-	0.026

		0.259					0.170		0.571	
DLG1	*Dlg1*	-0.413	-0.076	0.588	-0.139	0.300	-0.238	0.093	-0.373	0.003
DNA-PK	*Prkdc*	-0.237	-0.157	0.479	-0.085	0.682	-0.346	0.126	-0.229	0.246
DRAM	*Dram1*	0.084	-0.729	0.187	0.178	0.718	-0.064	0.903	-0.332	0.482
E-Cadherin	*Cdh1*	-0.251	0.025	0.945	0.151	0.656	-0.722	0.048	-0.405	0.200
E2F1	*E2f1*	-0.296	0.182	0.629	0.681	0.050	-0.128	0.741	-0.441	0.210
Elk-1	*Elk1*	-0.377	-0.063	0.808	0.045	0.852	-0.388	0.142	-0.412	0.077
FAK	*Ptk2*	-0.093	0.133	0.357	0.032	0.819	-0.038	0.797	0.021	0.873
FAS	*Fas*	0.107	0.015	0.932	0.319	0.043	0.096	0.570	0.187	0.212
FASN	*Fasn*	-0.367	-1.511	0.008	-1.297	0.014	-0.592	0.280	-1.562	0.001
FST	*Fst*	-0.524	-1.391	0.008	-1.190	0.015	-1.540	0.004	-1.968	0.000
G6PC	*G6pc*	0.183	-0.646	0.195	-0.285	0.543	0.167	0.733	0.657	0.133
GPD1	*Gpd1*	-0.415	-0.191	0.434	-0.330	0.155	-0.473	0.055	-0.071	0.740
Gsk3β	*Gsk3b*	0.117	0.144	0.273	0.063	0.614	0.006	0.966	0.095	0.416
HDAC	*Hdac1*	-0.330	-0.097	0.537	-0.041	0.785	0.024	0.878	-0.379	0.007
HDAC9	*Hdac9*	-0.109	-0.090	0.845	0.113	0.790	0.030	0.948	-0.550	0.200
HIF1A	*Hif1a*	0.060	0.008	0.954	-0.089	0.502	-0.113	0.421	-0.054	0.661
HIPK2	*Hipk2*	0.317	0.359	0.141	-0.016	0.945	0.068	0.782	0.299	0.172
HMGN1	*Hmgn1*	0.202	-0.053	0.698	-0.013	0.920	0.185	0.170	0.029	0.810
HTT	*Htt*	-0.338	-0.107	0.582	-0.185	0.315	-0.267	0.174	-0.313	0.070
IGF2	*Igf2*	-0.067	0.383	0.800	7.505	0.000	0.764	0.613	0.486	0.720
IGFBP1	*Igfbp1*	0.377	0.089	0.878	1.118	0.042	0.318	0.581	1.199	0.022
ILK	*Ilk*	-0.121	0.016	0.904	-0.076	0.539	-0.169	0.197	-0.152	0.188
INSR	*Insr*	0.507	0.106	0.572	-0.076	0.670	0.153	0.414	0.245	0.141
JMY	*Jmy*	0.286	0.096	0.554	-0.006	0.968	-0.056	0.731	0.257	0.072
JNK1	*Mapk8*	0.392	0.183	0.239	0.051	0.730	0.211	0.173	0.312	0.023
LEPR	*Lepr*	0.686	0.201	0.680	0.594	0.199	1.115	0.021	1.526	0.001
MDM2	*Mdm2*	0.001	0.116	0.521	0.217	0.203	0.048	0.791	0.120	0.456
MDM4	*Mdm4*	0.309	0.298	0.281	0.279	0.289	0.337	0.222	0.311	0.207
MKK3	*Map2k3*	0.044	0.262	0.106	0.221	0.152	0.193	0.233	-0.038	0.792
MLXIPL	*Mlxipl*	-0.493	-0.236	0.412	-0.743	0.007	-0.638	0.028	-0.520	0.040
Mup20	*Mup20*	-0.884	-1.845	0.255	-6.244	0.001	-6.679	0.001	-7.905	0.000
NFE2L2	*Nfe2l2*	-0.139	-0.035	0.828	-0.105	0.491	-0.146	0.365	-0.057	0.687

NR1I2	*Nr1i2*	0.805	0.306	0.204	0.498	0.030	0.782	0.001	0.783	0.000
NR2C2	*Nr2c2*	0.443	0.051	0.775	-0.013	0.937	0.082	0.641	0.216	0.166
NRF1	*Nrf1*	-0.120	-0.128	0.471	-0.168	0.317	-0.152	0.394	-0.087	0.581
OCLN	*Ocln*	0.248	-0.402	0.104	-0.181	0.434	-0.173	0.478	0.224	0.295
p130CAS	*Bcar1*	-0.613	-0.188	0.434	-0.271	0.235	-0.570	0.020	-0.893	0.000
p21Cip1	*Cdkn1a*	0.055	0.775	0.288	1.702	0.016	-0.253	0.736	1.225	0.066
p300	*Ep300*	0.476	0.129	0.501	0.097	0.593	0.087	0.650	0.432	0.011
p38 MAPK	*Mapk14*	0.614	0.219	0.157	0.243	0.099	0.306	0.048	0.436	0.002
p48	*St13*	-0.267	-0.042	0.777	-0.071	0.614	-0.130	0.384	-0.103	0.432
p53	*Trp53*	-0.159	0.136	0.551	0.326	0.131	-0.272	0.245	-0.076	0.711
p53R2	*Rrm2b*	0.386	0.497	0.054	0.215	0.390	0.496	0.055	0.556	0.016
PAI-1	*Serpine2*	-0.614	-0.181	0.579	-0.932	0.003	-1.420	0.000	-0.789	0.007
PALS2	*Mpp6*	0.277	0.094	0.606	-0.002	0.991	0.192	0.289	0.221	0.169
PCAF	*Kat2b*	0.424	0.184	0.354	-0.120	0.527	0.198	0.319	0.240	0.174
PCK1	*Pck1*	0.677	0.004	0.990	0.424	0.199	0.514	0.137	0.943	0.003
PCNA	*Pcna*	0.228	0.037	0.808	0.208	0.143	0.245	0.102	0.207	0.122
PDK1	*Pdpk1*	0.279	0.092	0.564	0.054	0.723	-0.018	0.910	0.220	0.120
PERP	*Perp*	0.317	-0.055	0.761	-0.129	0.450	0.106	0.551	0.236	0.136
PIAS1	*Pias1*	0.270	-0.171	0.269	-0.129	0.375	-0.044	0.774	0.151	0.259
PILT	*Tjap1*	-0.084	0.299	0.309	0.358	0.197	-0.289	0.352	0.097	0.715
PML	*Pml*	-0.243	-0.181	0.390	-0.134	0.500	-0.160	0.448	-0.263	0.157
PPARA	*Ppara*	0.257	-0.004	0.987	-0.055	0.811	0.114	0.637	-0.037	0.864
PPARG	*Pparg*	-0.012	0.249	0.483	0.534	0.113	0.024	0.947	-0.111	0.729
PPARGC1A	*Ppargc1a*	0.806	-0.172	0.624	0.249	0.452	0.623	0.071	1.064	0.001
PTEN	*Pten*	0.131	0.001	0.995	-0.097	0.595	0.069	0.717	0.041	0.807
PUMA	*Bbc3*	-0.311	-0.894	0.013	-0.599	0.071	-0.364	0.293	-0.659	0.033
PXN	*Pxn*	0.248	-0.126	0.367	0.059	0.652	-0.112	0.424	0.054	0.661
Rac	*Rac1*	-0.077	-0.032	0.801	-0.003	0.981	-0.037	0.773	-0.059	0.604
Rb	*Rb1*	-0.315	0.052	0.757	0.338	0.039	-0.251	0.146	-0.217	0.153
RORA	*Rora*	0.240	0.063	0.759	-0.141	0.470	0.063	0.758	-0.054	0.768
RORC	*Rorc*	0.817	0.610	0.053	0.696	0.021	1.162	0.000	1.088	0.000
SCD	*Scd1*	-0.123	-0.561	0.125	-0.670	0.053	-0.278	0.444	-0.438	0.169
SCO2	*Sco2*	0.082	-	0.714	0.026	0.942	0.427	0.251	0.310	0.354

			0.142							
SEPP1	*Sepp1*	0.386	0.167	0.376	0.116	0.521	0.284	0.133	0.247	0.141
SIRT	*Sirt1*	0.214	0.016	0.938	0.066	0.732	-0.119	0.560	0.299	0.092
Slug	*Snai2*	0.429	-0.004	0.988	0.037	0.889	0.139	0.617	0.319	0.194
SMARCB1	*Smarcb1*	-0.320	-0.075	0.759	0.152	0.507	-0.226	0.358	-0.477	0.029
STAG1	*Stag1*	0.180	0.023	0.880	-0.157	0.274	0.048	0.750	0.153	0.247
STAT3	*Stat3*	0.227	-0.364	0.151	0.348	0.142	0.043	0.862	0.271	0.223
Survivin	*Birc5*	-0.039	0.321	0.580	1.432	0.008	-0.403	0.507	0.053	0.920
SYMPK	*Sympk*	0.080	-0.071	0.652	0.061	0.681	-0.018	0.910	0.115	0.408
Teap	*Trp53inp1*	0.171	0.270	0.336	0.427	0.110	0.359	0.200	0.659	0.009
TGF-β	*Tgfb3*	-0.224	0.037	0.953	0.029	0.961	-0.691	0.296	-1.709	0.004
TGFBR3	*Tgfbr3*	0.436	0.114	0.564	-0.108	0.570	0.104	0.600	0.170	0.332
THRA	*Thra*	0.214	0.118	0.561	0.259	0.173	0.088	0.664	0.215	0.230
TNFR	*Tnfrsf1a*	-0.049	-0.297	0.103	0.063	0.712	-0.135	0.458	-0.168	0.293
TOPBP1	*Topbp1*	0.303	-0.238	0.201	0.137	0.424	0.097	0.592	0.133	0.406
TRAP220	*Med1*	0.350	0.083	0.572	0.036	0.798	-0.017	0.907	0.153	0.241
TSP1	*Thbs1*	-0.121	-0.291	0.683	0.505	0.447	-0.995	0.176	-1.492	0.021
VINCULIN	*Vcl*	-0.378	-0.070	0.692	-0.189	0.265	-0.408	0.024	-0.476	0.003
WASP	*Was*	-0.118	-0.151	0.704	0.245	0.503	-0.322	0.426	-0.585	0.107
WT1	*Wt1*	-0.140	0.136	0.846	0.389	0.555	-0.419	0.559	-1.344	0.040
ZAC1	*Plagl1*	-0.209	0.437	0.499	0.368	0.551	-0.597	0.377	-0.771	0.194
ZO2	*Tjp2*	-0.264	-0.261	0.199	-0.102	0.593	-0.227	0.264	-0.433	0.016
ZO3	*Tjp3*	-0.613	-0.435	0.112	-0.557	0.032	-0.795	0.004	-0.734	0.002

Table S7. Expression levels of genes involved in cancer signaling pathway correlated with the increase in calorie restriction (CR) and their gene expression at each level of CR relative to 12h ad libitum intake.

			10CR		20CR		30CR		40CR	
**Symbol**	**Gene Symbol**	**r**	**logFC**	**p**	**logFC**	**p**	**logFC**	**p**	**logFC**	**p**
APAF1	*Apaf1*	0.020	0.141	0.693	0.775	0.018	0.373	0.290	0.037	0.908
APC	*Apc*	0.413	0.334	0.112	0.247	0.219	0.332	0.115	0.452	0.016
ASK1	*Map3k5*	0.525	0.195	0.382	0.019	0.928	0.152	0.494	0.294	0.137
ATM	*Atm*	0.005	0.105	0.576	-0.050	0.780	0.011	0.954	-0.078	0.646
ATR	*Atr*	0.422	0.237	0.206	0.302	0.089	0.340	0.068	0.422	0.011
AXIN	*Axin1*	0.117	-0.146	0.373	-0.029	0.851	-0.154	0.349	0.088	0.540
B-RAF	*Braf*	0.417	-0.058	0.768	-0.284	0.132	0.114	0.560	0.303	0.080

BAD	*Bad*	0.476	-0.024	0.925	0.089	0.711	0.240	0.339	0.415	0.062
BAK	*Bak1*	-0.276	0.001	0.996	0.172	0.541	-0.274	0.363	-0.446	0.093
BAX	*Bax*	-0.138	-0.095	0.696	0.118	0.604	-0.205	0.403	-0.219	0.312
BCL2	*Bcl2*	-0.221	0.665	0.077	0.396	0.274	-0.449	0.265	-0.401	0.256
BclXL	*Bcl2l1*	-0.005	0.134	0.473	0.224	0.205	-0.077	0.684	0.065	0.695
BIM	*Bcl2l11*	0.546	0.078	0.774	0.328	0.199	0.371	0.167	0.574	0.016
BMPR2	*Bmpr2*	0.334	0.618	0.038	0.610	0.032	0.562	0.060	0.606	0.024
BRCA1	*Brca1*	0.463	0.008	0.985	0.734	0.055	0.903	0.024	1.072	0.003
c-Fos	*Fos*	0.121	-1.152	0.104	0.271	0.656	-0.193	0.770	0.217	0.705
c-Jun	*Jun*	-0.025	-0.027	0.943	0.834	0.017	-0.052	0.891	-0.014	0.966
c-RAF	*Raf1*	-0.299	-0.165	0.253	-0.215	0.116	-0.278	0.055	-0.247	0.052
C3G	*Rapgef1*	0.321	0.178	0.169	0.258	0.036	0.109	0.400	0.215	0.062
Caspase9	*Casp9*	-0.257	-0.279	0.109	-0.157	0.336	-0.164	0.344	-0.471	0.002
CBL	*Cbl*	0.217	-0.226	0.512	0.034	0.916	0.044	0.897	0.089	0.766
CBP	*Crebbp*	0.395	0.196	0.375	0.185	0.379	0.138	0.533	0.399	0.042
CCND1	*Ccnd1*	0.103	0.588	0.224	1.520	0.001	1.324	0.006	0.226	0.601
CDHE	*Cdh1*	-0.251	0.025	0.945	0.151	0.656	-0.722	0.048	-0.405	0.200
CDK2	*Cdk2*	0.027	0.145	0.420	-0.176	0.315	0.068	0.709	-0.078	0.631
CHK2	*Chek2*	-0.238	0.188	0.623	0.426	0.234	0.330	0.386	-0.226	0.524
CRK	*Crk*	0.303	0.242	0.138	0.011	0.943	0.190	0.245	0.274	0.060
CTNNβ	*Ctnnb1*	-0.363	-0.100	0.550	-0.083	0.601	-0.220	0.190	-0.268	0.069
CTNNδ	*Ctnnd1*	-0.070	-0.185	0.187	-0.089	0.502	-0.154	0.274	-0.124	0.314
DAXX	*Daxx*	-0.147	-0.233	0.212	-0.204	0.247	-0.389	0.040	-0.237	0.152
DNAPK	*Prkdc*	-0.237	-0.157	0.479	-0.085	0.682	-0.346	0.126	-0.229	0.246
DSH	*Dvl1*	0.490	-0.054	0.744	0.094	0.547	0.168	0.306	0.288	0.047
ELK-1	*Elk1*	-0.377	-0.063	0.808	0.045	0.852	-0.388	0.142	-0.412	0.077
EPAC1	*Rapgef3*	-0.192	0.200	0.525	0.094	0.754	-0.223	0.498	-0.083	0.774
FADD	*Fadd*	-0.480	-0.293	0.095	-0.301	0.070	-0.415	0.019	-0.565	0.000
FAK	*Ptk2*	-0.093	0.133	0.357	0.032	0.819	-0.038	0.797	0.021	0.873
FAS	*Fas*	0.107	0.015	0.932	0.319	0.043	0.096	0.570	0.187	0.212
FLIP	*Cflar*	0.329	0.243	0.163	0.046	0.782	0.211	0.225	0.278	0.072
FOXO1	*Foxo1*	0.603	0.236	0.302	0.322	0.139	0.382	0.094	0.681	0.001
FYN	*Fyn*	-0.297	0.002	0.993	0.118	0.519	0.012	0.953	-0.240	0.171
GRB2	*Grb2*	-0.346	-0.098	0.434	0.003	0.982	-0.104	0.405	-0.123	0.266
GSK3β	*Gsk3b*	0.117	0.144	0.273	0.063	0.614	0.006	0.966	0.095	0.416
HAT1	*Hat1*	-0.099	0.082	0.702	0.107	0.596	0.106	0.620	-0.017	0.929
HIF1α	*Hif1a*	0.060	0.008	0.954	-0.089	0.502	-0.113	0.421	-0.054	0.661
HIPK2	*Hipk2*	0.317	0.359	0.141	-0.016	0.945	0.068	0.782	0.299	0.172
IRS1	*Irs1*	0.120	-0.229	0.408	-0.191	0.466	-0.129	0.639	0.412	0.089
MAX	*Max*	-0.149	0.166	0.250	0.098	0.476	-0.038	0.795	-0.022	0.866
MDM2	*Mdm2*	0.001	0.116	0.521	0.217	0.203	0.048	0.791	0.120	0.456
MIZ1	*Zbtb17*	-0.167	-0.035	0.846	-0.020	0.907	-0.122	0.500	-0.181	0.262
MYC	*Myc*	-0.042	0.872	0.151	0.459	0.431	0.226	0.713	-0.618	0.261
NBS1	*Nbn*	0.141	0.014	0.925	-0.089	0.534	-0.079	0.604	0.024	0.857
NCSTN	*Ncstn*	0.043	-0.233	0.123	0.029	0.840	-0.005	0.972	-0.009	0.944
NF1	*Nf1*	0.031	0.026	0.875	-0.134	0.388	-0.085	0.603	0.005	0.972
NICD	*Notch1*	0.150	-0.173	0.398	0.081	0.675	0.095	0.640	0.226	0.207
NLK	*Nlk*	-0.352	0.120	0.533	-0.227	0.224	-0.041	0.834	-0.212	0.226
p18INK4C	*Cdkn2c*	0.118	0.474	0.161	0.528	0.101	0.653	0.052	0.545	0.073
p21CIP1	*Cdkn1a*	0.055	0.775	0.288	1.702	0.016	-0.253	0.736	1.225	0.066
p27KIP1	*Cdkn1b*	-0.152	0.054	0.745	-0.047	0.766	-0.197	0.243	-0.047	0.748
p300	*Ep300*	0.476	0.129	0.501	0.097	0.593	0.087	0.650	0.432	0.011
p53	*Trp53*	-0.159	0.136	0.551	0.326	0.131	-0.272	0.245	-0.076	0.711

PTCH	*Ptch1*	0.204	-0.103	0.684	-0.136	0.572	-0.397	0.125	0.184	0.410
PUMA	*Bbc3*	-0.311	-0.894	0.013	-0.599	0.071	-0.364	0.293	-0.659	0.033
RalGAP	*Ralbp1*	-0.357	0.061	0.689	0.004	0.980	-0.136	0.373	-0.318	0.019
RALGEF	*Ralgds*	-0.171	0.225	0.595	0.514	0.198	0.222	0.602	-0.368	0.345
RASGAP	*Rasa1*	0.001	0.057	0.695	-0.090	0.516	-0.034	0.818	-0.048	0.710
RASGRP	*Rasgrp1*	-0.187	-0.031	0.932	0.175	0.608	0.029	0.936	-0.592	0.077
Rb	*Rb1*	-0.315	0.052	0.757	-0.338	0.039	-0.251	0.146	-0.217	0.153
RBL1	*Rbl1*	-0.021	0.645	0.161	0.934	0.032	0.086	0.858	-0.011	0.980
RBPJK	*Rbpj*	-0.100	0.052	0.722	-0.083	0.554	-0.033	0.825	-0.110	0.402
SHC	*Shc1*	-0.180	-0.091	0.512	0.026	0.845	-0.072	0.607	-0.258	0.036
SHP2	*Ptpn11*	0.124	0.155	0.295	0.114	0.418	0.045	0.764	0.179	0.173
SMAC	*Diablo*	-0.164	-0.006	0.968	0.107	0.482	-0.172	0.298	-0.090	0.533
SMAD4	*Smad4*	0.360	0.133	0.407	-0.007	0.962	0.110	0.493	0.229	0.106
SMAD6	*Smad6*	0.041	-0.118	0.627	0.074	0.744	-0.081	0.740	-0.183	0.399
SMAD7	*Smad7*	0.052	0.229	0.338	0.123	0.588	0.431	0.069	-0.247	0.254
SMO	*Smo*	-0.431	-0.320	0.397	-0.254	0.477	-0.951	0.015	-1.234	0.000
SRC	*Src*	-0.082	0.633	0.301	1.359	0.020	-0.224	0.724	0.037	0.947
STK6	*Aurka*	-0.053	-0.194	0.695	0.913	0.040	-0.333	0.507	0.189	0.661
SUFU	*Sufu*	0.011	-0.010	0.978	0.343	0.298	0.214	0.547	0.094	0.768
TAB1	*Tab2*	0.270	-0.086	0.639	-0.147	0.397	-0.244	0.183	0.322	0.046
tBID	*Bid*	-0.364	-0.040	0.835	-0.201	0.275	-0.184	0.345	-0.408	0.019
TGFβR1	*Tgfbr1*	0.221	0.328	0.077	0.288	0.103	0.280	0.132	0.358	0.031
TGFβR2	*Tgfbr2*	0.066	0.076	0.665	0.151	0.363	0.055	0.755	0.032	0.835
WNT5A	*Wnt5a*	0.004	0.322	0.584	0.458	0.413	-0.300	0.628	-0.209	0.700

Table S8. Expression levels of genes involved in autophagy pathway correlated with the increase in calorie restriction (CR) and their gene expression at each level of CR relative to 12h ad libitum intake.

			10CR		20CR		30CR		40CR	
**Symbol**	**Gene Symbol**	**r**	**logFC**	**p**	**logFC**	**p**	**logFC**	**p**	**logFC**	**p**
ATG10	*Atg10*	-0.125	0.083	0.725	-0.177	0.441	0.043	0.856	-0.163	0.448
ATG12	*Atg12*	-0.095	0.089	0.566	0.096	0.513	-0.052	0.738	-0.007	0.960
ATG13	*Atg13*	-0.003	-0.132	0.372	-0.241	0.088	-0.143	0.335	-0.097	0.458
ATG16L1	*Atg16l1*	0.651	0.036	0.842	0.033	0.848	0.144	0.427	0.466	0.004
ATG3	*Atg3*	0.411	0.006	0.972	-0.094	0.553	0.188	0.256	0.160	0.278
ATG5	*Atg5*	0.319	0.052	0.731	0.087	0.546	0.167	0.269	0.163	0.226
ATG7	*Atg7*	-0.403	-0.189	0.229	-0.108	0.465	-0.277	0.080	-0.358	0.010
BCL2	*Bcl2*	-0.221	0.665	0.077	0.396	0.274	-0.449	0.265	-0.401	0.256
BECN1	*Becn1*	-0.366	0.115	0.361	-0.052	0.665	-0.123	0.333	-0.024	0.831
FIP200	*Rb1cc1*	0.399	0.184	0.299	0.049	0.773	0.171	0.335	0.273	0.083
LAMP1	*Lamp1*	-0.301	-0.006	0.973	0.007	0.963	-0.116	0.472	-0.100	0.478
LAMP2	*Lamp2*	-0.611	-0.130	0.494	-0.260	0.152	-0.272	0.155	-0.192	0.253
LC3-II	*Map1lc3a*	0.450	-0.003	0.986	0.106	0.572	0.183	0.353	0.497	0.004
LC3-II	*Map1lc3b*	0.486	-0.009	0.956	0.133	0.369	0.109	0.485	0.238	0.085
mTOR	*Mtor*	-0.119	0.060	0.704	0.159	0.289	0.043	0.787	-0.122	0.384
NBR1	*Nbr1*	0.080	-0.032	0.843	-0.176	0.249	-0.051	0.752	-0.015	0.915
SQSTM1	*Sqstm1*	-0.287	0.000	0.999	0.200	0.481	-0.483	0.110	-0.475	0.072
STX17	*Stx17*	0.403	0.117	0.464	-0.034	0.824	0.199	0.210	0.160	0.257
ULK1	*Ulk1*	-0.040	-0.384	0.029	-0.214	0.197	-0.290	0.098	-0.103	0.504
WDFY3	*Wdfy3*	0.471	0.120	0.531	0.106	0.561	0.198	0.302	0.309	0.070
WIPI1	*Wipi1*	0.054	-0.500	0.019	-0.287	0.148	-0.371	0.079	-0.174	0.344

Table S9. Expression levels of genes involved in fuel utilization correlated with the increase in calorie restriction (CR) and their gene expression at each level of CR relative to 12h ad libitum intake.

Symbol	Gene Symbol	r	10CR logFC	10CR p	20CR logFC	20CR p	30CR logFC	30CR p	40CR logFC	40CR p
ABAD	*Hsd17b10*	0.523	-0.002	0.991	-0.016	0.930	0.165	0.382	0.322	0.055
ACAA1	*Acaa1a*	0.364	0.098	0.609	0.178	0.323	0.286	0.129	0.158	0.346
Acaa1b	*Acaa1b*	0.340	0.334	0.329	0.184	0.574	0.655	0.055	0.551	0.072
ACAA2	*Acaa2*	0.309	0.054	0.780	-0.024	0.896	0.132	0.493	0.205	0.230
ACADM	*Acadm*	0.561	0.138	0.510	0.243	0.222	0.384	0.065	0.601	0.001
ACO1	*Aco1*	0.057	-0.037	0.829	-0.085	0.606	-0.056	0.746	-0.013	0.932
ACO2	*Aco2*	0.698	0.123	0.600	0.349	0.117	0.513	0.028	0.652	0.002
ACSL1	*Acsl1*	0.247	0.390	0.179	-0.073	0.794	0.234	0.422	0.143	0.581
ACSL3	*Acsl3*	0.024	-0.361	0.132	-0.082	0.715	0.088	0.711	-0.402	0.056
ACSL4	*Acsl4*	-0.161	0.140	0.550	0.298	0.179	0.062	0.792	-0.162	0.434
ACSL5	*Acsl5*	-0.021	-0.622	0.011	-0.484	0.035	-0.320	0.185	-0.242	0.254
ADRB3	*Adrb3*	-0.524	-0.341	0.498	-1.337	0.007	-0.883	0.087	-1.689	0.000
AIF	*Aifm1*	0.086	-0.143	0.341	-0.080	0.574	-0.018	0.902	0.062	0.640
ALDOA	*Aldoa*	0.291	-0.060	0.753	0.237	0.191	0.093	0.625	0.226	0.181
ALDOB	*Aldob*	0.504	-0.169	0.509	-0.091	0.705	0.174	0.494	0.452	0.046
ALDOC	*Aldoc*	0.152	-0.490	0.215	0.132	0.721	0.167	0.665	-0.176	0.608
APP	*App*	-0.016	0.306	0.340	0.670	0.028	0.244	0.448	0.269	0.347
ATP5A1	*Atp5a1*	0.575	-0.066	0.715	-0.075	0.660	0.141	0.431	0.222	0.163
ATP5B	*Atp5b*	0.608	0.007	0.968	0.061	0.725	0.211	0.244	0.352	0.029
ATP5C1	*Atp5c1*	0.683	0.085	0.650	0.090	0.615	0.302	0.106	0.362	0.030
ATP5D	*Atp5d*	0.348	-0.031	0.846	0.056	0.708	0.065	0.679	0.176	0.205
ATP5F1	*Atp5f1*	0.662	0.036	0.831	0.033	0.838	0.280	0.099	0.316	0.037
ATP5G3	*Atp5g3*	0.520	0.163	0.313	0.005	0.972	0.301	0.061	0.313	0.029
ATP5H	*Atp5h*	0.687	-0.067	0.717	-0.014	0.937	0.266	0.149	0.381	0.020
ATP5J	*Atp5j*	0.267	0.038	0.814	-0.120	0.436	0.138	0.393	0.099	0.489
ATP5J2	*Atp5j2*	0.360	0.175	0.317	-0.007	0.966	0.267	0.125	0.255	0.100
AUH	*Auh*	0.032	0.079	0.611	-0.119	0.422	0.004	0.978	0.001	0.995
BCL2	*Bcl2*	-0.221	0.665	0.077	0.396	0.274	-0.449	0.265	-0.401	0.256
BECN1	*Becn1*	-0.366	0.115	0.361	-0.052	0.665	-0.123	0.333	-0.024	0.831
BPGM	*Bpgm*	-0.142	-0.084	0.666	0.123	0.500	-0.226	0.251	0.207	0.226
CAMK4	*Camk4*	0.023	0.409	0.340	0.055	0.896	-0.389	0.397	0.076	0.845
Caspase 3	*Casp3*	-0.315	-0.439	0.038	-0.312	0.115	-0.059	0.774	-0.472	0.011
Caspase 8	*Casp8*	0.075	0.093	0.508	0.039	0.770	0.047	0.737	-0.072	0.568
Caspase 9	*Casp9*	-0.257	-0.279	0.109	-0.157	0.336	-0.164	0.344	-0.471	0.002
CAT	*Cat*	-0.805	-0.091	0.700	-0.470	0.039	-0.487	0.043	-0.668	0.001
CHUK	*Chuk*	0.065	0.152	0.458	-0.183	0.349	0.035	0.865	-0.021	0.908
COX4I1	*Cox4i1*	0.429	-0.070	0.693	0.002	0.993	0.155	0.381	0.202	0.199
COX5A	*Cox5a*	0.576	-0.010	0.952	-0.054	0.740	0.230	0.176	0.293	0.053

COX6A1	*Cox6a1*	0.406	0.082	0.596	-0.055	0.706	0.049	0.752	0.205	0.135
COX6B1	*Cox6b1*	0.367	0.100	0.548	-0.003	0.987	0.158	0.344	0.229	0.122
COX7A1	*Cox7a1*	0.820	0.405	0.386	0.721	0.099	1.188	0.008	1.585	0.000
COX7A2	*Cox7a2*	-0.035	0.050	0.751	-0.173	0.300	-0.024	0.893	-0.017	0.911
COX7B	*Cox7b*	0.545	0.179	0.410	-0.066	0.752	0.400	0.065	0.404	0.037
COX8A	*Cox8a*	0.627	0.119	0.465	-0.027	0.863	0.247	0.130	0.298	0.040
CRTC3	*Crtc3*	-0.027	-0.254	0.137	-0.181	0.258	-0.286	0.096	-0.041	0.780
CS	*Cs*	-0.012	-0.604	0.005	-0.502	0.014	-0.238	0.266	-0.259	0.170
DHOH	*Dhodh*	-0.254	-0.337	0.151	0.007	0.972	-0.111	0.627	-0.262	0.204
DHTKD1	*Dhtkd1*	0.515	0.213	0.316	0.040	0.842	0.355	0.094	0.333	0.078
DJ - 1	*Park7*	0.310	0.002	0.986	-0.045	0.741	0.115	0.416	0.181	0.149
DLD	*Dld*	0.453	0.035	0.817	-0.081	0.570	0.093	0.534	0.243	0.067
DLST	*Dlst*	0.479	0.107	0.519	0.124	0.433	0.242	0.144	0.311	0.035
ECHS1	*Echs1*	0.632	-0.040	0.833	-0.011	0.950	0.319	0.092	0.289	0.086
ECI1	*Eci1*	0.315	0.098	0.596	0.029	0.870	0.364	0.048	0.136	0.408
ECI2	*Eci2*	0.519	0.378	0.164	0.191	0.461	0.501	0.065	0.425	0.080
EHHADH	*Ehhadh*	0.721	0.463	0.318	1.047	0.019	1.570	0.001	1.803	0.000
ENO1	*Eno1*	0.524	-0.496	0.113	-0.147	0.615	0.086	0.778	0.433	0.108
ENO3	*Eno3*	0.077	0.256	0.361	-0.183	0.507	-0.074	0.798	-0.012	0.962
FBP1	*Fbp1*	0.117	-0.036	0.851	-0.076	0.681	0.079	0.682	0.065	0.702
FH	*Fh1*	0.161	-0.215	0.233	-0.012	0.946	-0.067	0.709	0.071	0.653
FIS1	*Fis1*	0.128	-0.052	0.752	0.094	0.545	0.045	0.784	0.071	0.624
FURIN	*Furin*	0.598	-0.163	0.353	-0.128	0.441	-0.020	0.908	0.205	0.186
GAPDH	*Gapdh*	0.360	-0.139	0.484	-0.112	0.552	-0.076	0.701	0.262	0.134
GNAS	*Gnas*	-0.311	0.014	0.928	-0.036	0.811	-0.116	0.459	-0.069	0.620
GPD2	*Gpd2*	0.235	-0.267	0.329	-0.510	0.050	-0.056	0.837	0.147	0.541
GPI	*Gpi1*	0.571	-0.176	0.505	0.023	0.926	0.187	0.474	0.625	0.007
GPX4	*Gpx4*	0.057	0.208	0.384	0.575	0.011	0.274	0.251	0.284	0.182
GPX7	*Gpx7*	0.074	0.809	0.208	1.710	0.005	0.803	0.215	0.573	0.329
GRX2	*Glrx2*	0.014	0.050	0.737	-0.113	0.423	0.041	0.784	0.070	0.592
GSR	*Gsr*	-0.153	0.054	0.710	0.121	0.376	-0.187	0.198	-0.034	0.788
HADH	*Hadh*	0.620	0.147	0.475	0.343	0.080	0.565	0.006	0.511	0.005
HADHA	*Hadha*	0.377	0.038	0.833	0.067	0.693	0.044	0.805	0.178	0.262
HSD17B10	*Hsd17b10*	0.523	-0.002	0.991	-0.016	0.930	0.165	0.382	0.322	0.055
HSD17B4	*Hsd17b4*	0.149	-0.001	0.997	-0.060	0.723	0.055	0.758	0.166	0.295
HSD17B8	*H2-Ke6*	-0.084	-0.332	0.043	-0.167	0.281	-0.228	0.163	-0.099	0.489
HtrA2	*Htra2*	0.193	-0.097	0.668	0.174	0.408	0.151	0.497	-0.007	0.970
HTT	*Htt*	-0.338	-0.107	0.582	-0.185	0.315	-0.267	0.174	-0.313	0.070
IDH3A	*Idh3a*	0.806	0.181	0.412	0.233	0.266	0.541	0.013	0.850	0.000
IDH3B	*Idh3b*	0.631	0.148	0.324	0.073	0.611	0.256	0.088	0.300	0.024
IDH3G	*Idh3g*	0.371	0.054	0.668	-0.030	0.805	0.104	0.413	0.178	0.113
IL15	*Il15*	0.392	0.490	0.203	0.285	0.446	0.576	0.133	0.482	0.165
IVD	*Ivd*	0.083	0.075	0.626	0.006	0.969	0.091	0.551	0.057	0.675
KGDH	*Ogdh*	0.350	-0.016	0.923	0.030	0.849	0.021	0.900	0.117	0.428
LETM1	*Letm1*	-0.130	-0.091	0.533	-0.139	0.315	-0.284	0.054	0.067	0.603

LRRK2	*Lrrk2*	0.014	0.417	0.191	0.147	0.636	0.226	0.488	-0.107	0.721
MAOA	*Maoa*	0.678	0.006	0.974	0.224	0.203	0.559	0.002	0.559	0.001
MAOB	*Maob*	0.756	0.141	0.522	0.283	0.177	0.603	0.006	0.680	0.001
MDH1	*Mdh1*	0.205	-0.074	0.681	-0.112	0.514	0.035	0.848	0.028	0.862
MDH2	*Mdh2*	0.482	-0.093	0.652	0.258	0.185	0.204	0.320	0.518	0.005
ME1	*Me1*	0.029	-0.743	0.073	-0.928	0.018	-0.228	0.577	-0.288	0.423
ME2	*Me2*	-0.006	0.269	0.424	0.461	0.146	0.228	0.502	-0.076	0.803
MIRO2	*Rhot2*	0.348	0.116	0.416	0.148	0.274	0.171	0.231	0.300	0.018
MKK4	*Map2k4*	0.197	0.077	0.479	0.047	0.645	0.102	0.344	0.109	0.258
mtSOD	*Sod2*	0.568	0.162	0.327	0.034	0.827	0.255	0.122	0.320	0.029
NCT	*Ncstn*	0.043	-0.233	0.123	0.029	0.840	-0.005	0.972	-0.009	0.944
NDUFA1	*Ndufa11*	0.343	-0.008	0.966	-0.061	0.720	0.043	0.813	0.309	0.052
NDUFA10	*Ndufa10*	0.380	-0.059	0.689	-0.060	0.667	0.067	0.650	0.178	0.171
NDUFA12	*Ndufa12*	0.621	0.163	0.382	-0.044	0.803	0.238	0.199	0.529	0.001
NDUFA13	*Ndufa13*	-0.200	-0.008	0.956	-0.104	0.471	-0.102	0.503	-0.041	0.761
NDUFA2	*Ndufa2*	0.152	0.046	0.808	-0.218	0.231	0.171	0.363	0.176	0.293
NDUFA3	*Ndufa3*	0.193	0.062	0.725	-0.118	0.487	0.091	0.609	0.205	0.192
NDUFA4	*Ndufa4*	0.235	0.008	0.967	-0.215	0.256	0.023	0.906	0.173	0.326
NDUFA5	*Ndufa5*	0.543	0.156	0.443	-0.052	0.790	0.459	0.022	0.396	0.027
NDUFA6	*Ndufa6*	0.263	0.007	0.975	-0.353	0.084	0.022	0.917	0.174	0.357
NDUFA7	*Ndufa7*	0.634	0.083	0.645	0.182	0.286	0.388	0.029	0.509	0.001
NDUFA8	*Ndufa8*	0.619	-0.072	0.665	0.003	0.986	0.108	0.514	0.355	0.016
NDUFA9	*Ndufa9*	0.506	-0.136	0.385	-0.142	0.340	0.097	0.535	0.240	0.082
NDUFAF1	*Ndufaf1*	-0.610	-0.265	0.184	-0.352	0.064	-0.390	0.053	-0.460	0.009
NDUFB10	*Ndufb10*	0.543	-0.001	0.995	0.011	0.939	0.205	0.185	0.300	0.029
NDUFB11	*Ndufb11*	0.296	-0.105	0.560	-0.182	0.289	0.071	0.694	0.170	0.287
NDUFB2	*Ndufb2*	0.001	0.182	0.328	0.054	0.759	0.065	0.728	0.111	0.503
NDUFB3	*Ndufb3*	0.139	0.030	0.867	-0.329	0.059	0.021	0.906	-0.005	0.975
NDUFB4	*Ndufb4*	0.336	0.126	0.486	0.133	0.437	0.234	0.194	0.306	0.056
NDUFB5	*Ndufb5*	0.524	0.028	0.859	-0.107	0.474	0.202	0.194	0.242	0.080
NDUFB6	*Ndufb6*	0.463	-0.075	0.683	-0.129	0.460	0.235	0.195	0.313	0.053
NDUFB7	*Ndufb7*	0.210	-0.112	0.460	-0.112	0.436	0.094	0.533	0.100	0.454
NDUFB8	*Ndufb8*	0.569	0.000	0.998	-0.160	0.404	0.328	0.102	0.320	0.074
NDUFB9	*Ndufb9*	0.534	0.048	0.799	0.000	0.999	0.231	0.216	0.264	0.112
NDUFS1	*Ndufs1*	0.478	-0.006	0.967	-0.111	0.427	0.180	0.219	0.164	0.208
NDUFS2	*Ndufs2*	0.625	0.013	0.932	0.036	0.798	0.119	0.423	0.305	0.021
NDUFS4	*Ndufs4*	0.355	-0.007	0.966	-0.166	0.300	0.263	0.113	0.105	0.477
NDUFS6	*Ndufs6*	-0.050	0.121	0.547	-0.091	0.555	0.001	0.994	0.065	0.648
NDUFS7	*Ndufs7*	0.219	-0.141	0.391	-0.140	0.371	-0.018	0.914	0.065	0.652
NDUFS8	*Ndufs8*	0.027	-0.019	0.899	0.063	0.651	0.065	0.654	-0.011	0.935
NDUFV1	*Ndufv1*	0.572	-0.139	0.404	-0.064	0.686	0.002	0.988	0.280	0.057
NDUFV2	*Ndufv2*	0.592	0.097	0.572	0.012	0.942	0.273	0.112	0.405	0.008
NDUFV3	*Ndufv3*	0.374	0.102	0.491	0.025	0.858	0.089	0.548	0.223	0.088
NOS3	*Nos3*	-0.093	-0.404	0.219	0.012	0.968	-0.140	0.663	-0.345	0.232
NR1D1	*Nr1d1*	-0.724	-0.966	0.028	-1.327	0.002	-1.619	0.000	-1.734	0.000

OGDH	*Ogdh*	0.350	-0.016	0.923	0.030	0.849	0.021	0.900	0.117	0.428
Parkin	*Park2*	0.012	0.161	0.636	-0.111	0.734	0.515	0.121	-0.210	0.496
PDHA	*Pdha1*	0.650	0.140	0.497	-0.122	0.533	0.296	0.148	0.367	0.045
PFKL	*Pfkl*	-0.327	-0.080	0.672	0.056	0.753	-0.087	0.648	-0.128	0.448
PFKM	*Pfkm*	0.407	-0.325	0.099	-0.114	0.536	0.205	0.282	-0.001	0.993
PFKP	*Pfkp*	0.187	0.393	0.179	0.529	0.057	0.238	0.419	0.323	0.217
PGAM1	*Pgam1*	0.383	0.018	0.911	0.009	0.953	0.039	0.803	0.328	0.018
PGK1	*Pgk1*	0.756	-0.019	0.925	0.161	0.407	0.353	0.083	0.616	0.001
PINK1	*Pink1*	0.640	-0.059	0.736	0.072	0.663	0.211	0.224	0.305	0.049
PKLR	*Pklr*	-0.281	-0.556	0.146	-0.928	0.011	-0.253	0.505	-0.495	0.137
PKM	*Pkm*	-0.155	-0.011	0.971	0.418	0.148	-0.176	0.569	-0.358	0.188
PPARGC1A	*Ppargc1a*	0.806	-0.172	0.624	0.249	0.452	0.623	0.071	1.064	0.001
PPARGC1B	*Ppargc1b*	0.189	0.051	0.824	-0.193	0.380	-0.001	0.998	-0.106	0.605
PRX3	*Prdx3*	0.266	-0.107	0.511	0.132	0.391	0.166	0.306	0.171	0.234
PRX5	*Prdx5*	-0.083	0.021	0.892	-0.019	0.897	-0.041	0.789	-0.007	0.962
PSEN1	*Psen1*	-0.026	-0.026	0.820	0.078	0.477	-0.049	0.677	0.013	0.896
PTEN	*Pten*	0.131	0.001	0.995	-0.097	0.595	0.069	0.717	0.041	0.807
SCP2	*Scp2*	-0.808	-0.096	0.729	-0.584	0.028	-0.608	0.031	-0.742	0.002
SDHA	*Sdha*	0.185	-0.084	0.658	-0.239	0.185	0.027	0.886	0.013	0.937
SDHB	*Sdhb*	0.462	0.127	0.474	-0.026	0.876	0.195	0.271	0.193	0.220
SDHC	*Sdhc*	0.632	-0.009	0.957	0.101	0.502	0.244	0.121	0.251	0.073
SDHD	*Sdhd*	0.735	-0.042	0.850	-0.024	0.909	0.411	0.063	0.435	0.028
SDS	*Sds*	0.552	-0.177	0.474	0.353	0.130	0.513	0.035	0.445	0.042
SIRT1	*Sirt1*	0.214	0.016	0.938	0.066	0.732	-0.119	0.560	0.299	0.092
SLC27A1	*Slc27a1*	0.243	0.426	0.232	0.558	0.098	0.507	0.153	0.413	0.195
SLC27A2	*Slc27a2*	-0.093	0.095	0.659	-0.196	0.340	-0.106	0.622	-0.149	0.434
SLC27A4	*Slc27a4*	-0.326	-0.168	0.379	-0.070	0.699	-0.309	0.109	-0.298	0.078
SLC27A5	*Slc27a5*	0.118	-0.290	0.277	-0.112	0.657	-0.093	0.727	0.005	0.982
SUCLA2	*Sucla2*	0.370	0.007	0.963	-0.121	0.409	0.114	0.461	0.183	0.181
SUCLG1	*Suclg1*	0.279	-0.007	0.963	0.015	0.923	0.156	0.331	0.137	0.338
Synucleinα	*Snca*	-0.087	0.131	0.862	1.040	0.130	0.415	0.575	0.818	0.210
TFAM	*Tfam*	0.426	-0.001	0.992	-0.054	0.709	0.217	0.150	0.217	0.105
TPI1	*Tpi1*	0.483	-0.182	0.331	0.011	0.951	0.104	0.577	0.331	0.045
TRAK1	*Trak1*	0.678	0.125	0.502	0.095	0.591	0.419	0.023	0.301	0.068
TRX2	*Txn2*	0.583	0.027	0.862	0.236	0.109	0.181	0.242	0.340	0.014
TRXR2	*Txnrd2*	0.093	-0.187	0.271	-0.124	0.437	-0.015	0.931	0.033	0.825
UCP2	*Ucp2*	-0.133	0.204	0.479	0.582	0.033	0.177	0.540	-0.190	0.458
UQCR10	*Uqcr10*	0.369	0.246	0.170	0.020	0.907	0.319	0.074	0.419	0.008
UQCRB	*Uqcrb*	0.516	0.178	0.401	-0.102	0.618	0.259	0.226	0.308	0.106
UQCRC1	*Uqcrc1*	0.507	-0.064	0.712	-0.068	0.684	0.090	0.606	0.228	0.140
UQCRC2	*Uqcrc2*	0.688	0.012	0.944	-0.043	0.795	0.194	0.263	0.331	0.032
UQCRFS1	*Uqcrfs1*	0.618	0.012	0.942	-0.067	0.660	0.212	0.181	0.320	0.023
Xanthine oxidase	*Xdh*	0.107	0.077	0.634	0.158	0.303	-0.013	0.937	0.036	0.799

Table S10. Expression levels of genes involved in H2S production and signaling pathway correlated with the increase in calorie restriction (CR) and their gene expression at each level of CR relative to 12h ad libitum intake.

			10CR		20CR		30CR		40CR	
Symbol	Gene Symbol	r	logFC	p	logFC	p	logFC	p	logFC	p
CBS/CBSL	*Cbs*	-0.549	-0.626	0.021	-0.500	0.050	-0.701	0.010	-0.565	0.016
CRY1	*Cry1*	0.785	0.676	0.138	1.026	0.018	1.333	0.003	1.800	0.000
CRY2	*Cry2*	0.441	0.071	0.687	0.056	0.741	0.189	0.284	0.159	0.308
CTH	*Cth*	0.550	0.268	0.302	0.461	0.063	0.550	0.034	1.054	0.000
PER1	*Per1*	0.849	0.284	0.455	0.544	0.132	0.868	0.021	1.301	0.000
PER2	*Per2*	0.724	0.671	0.039	0.828	0.008	1.028	0.002	0.950	0.001
SIRT1	*Sirt1*	0.214	0.016	0.938	0.066	0.732	-0.119	0.560	0.299	0.092
FGF21	*Fgf21*	-0.076	0.369	0.614	0.588	0.399	-0.948	0.215	-0.960	0.146

Table S11. Expression levels of genes involved in xenobiotic metabolism correlated with the increase in calorie restriction (CR) and their gene expression at each level of CR relative to 12h ad libitum intake.

			10CR		20CR		30CR		40CR	
Symbol	Gene Symbol	r	logFC	p	logFC	p	logFC	p	logFC	p
ABCA1	*Abca1*	-0.518	-0.087	0.602	-0.172	0.280	-0.230	0.169	-0.264	0.073
ABCG1	*Abcg1*	-0.038	0.130	0.615	0.320	0.190	0.041	0.873	-0.302	0.192
ABCG5	*Abcg5*	0.286	0.287	0.179	0.106	0.604	0.276	0.196	0.139	0.464
ABCG8	*Abcg8*	-0.078	-0.054	0.805	-0.279	0.179	-0.140	0.522	-0.319	0.097
AE2	*Slc4a2*	0.109	0.029	0.861	0.205	0.185	-0.107	0.518	0.010	0.945
AHR	*Ahr*	0.468	-0.012	0.957	0.117	0.564	0.293	0.166	0.422	0.025
ALAS1	*Alas1*	0.595	0.988	0.033	1.105	0.013	1.421	0.002	0.913	0.030
ALDH1A1	*Aldh1a1*	-0.379	0.234	0.324	-0.117	0.606	-0.105	0.660	-0.037	0.860
ALDH1A7	*Aldh1a7*	-0.034	0.029	0.894	0.172	0.401	0.144	0.504	-0.002	0.991
ALDH3A2	*Aldh3a2*	0.595	0.450	0.133	0.489	0.088	0.771	0.010	0.882	0.001
ANKRA2	*Ankra2*	-0.150	0.140	0.518	-0.085	0.685	0.060	0.782	-0.153	0.436
ApoA1	*Apoa1*	-0.468	-0.682	0.033	-0.354	0.239	-0.588	0.066	-0.797	0.004
ApoB	*Apob*	-0.239	0.044	0.827	-0.174	0.358	-0.202	0.313	-0.057	0.747
APOC2	*Apoc2*	0.073	0.211	0.366	0.551	0.013	0.171	0.464	0.218	0.293
APOE	*Apoe*	-0.364	-0.248	0.238	-0.216	0.277	-0.331	0.116	-0.233	0.207
ARNT	*Arnt*	0.457	0.085	0.566	0.091	0.519	0.273	0.063	0.201	0.126
ASBT	*Slc10a2*	-0.623	-0.542	0.345	-0.396	0.463	-1.162	0.049	-1.764	0.001
BAAT	*Baat*	-0.151	0.050	0.809	-0.180	0.358	-0.015	0.943	0.163	0.372
BACS	*Slc27a5*	0.118	-0.290	0.277	-0.112	0.657	-0.093	0.727	0.005	0.982
BSEP	*Abcb11*	-0.627	-0.395	0.138	-0.610	0.016	-0.552	0.039	-0.446	0.055
CAR	*Nr1i3*	0.691	0.674	0.140	0.656	0.133	1.327	0.004	1.153	0.005
CAT	*Cat*	-0.805	-0.091	0.700	-0.470	0.039	-0.487	0.043	-0.668	0.001
CCRP	*Dnajc7*	0.217	-0.023	0.850	-0.153	0.183	-0.004	0.971	0.020	0.853
CD14	*Cd14*	-0.154	0.506	0.365	0.982	0.064	-0.159	0.782	-0.274	0.589
CES3	*Ces3a*	-0.924	-0.247	0.769	-1.607	0.051	-2.929	0.002	-4.669	0.000
ChREBP	*Mlxipl*	-0.493	-0.236	0.412	-0.743	0.007	-0.638	0.028	-0.520	0.040
c-Jun	*Jun*	-0.025	-0.027	0.943	0.834	0.017	-0.052	0.891	-0.014	0.966
CPT1A	*Cpt1a*	0.390	0.173	0.452	0.285	0.193	0.300	0.191	0.191	0.352
c-RAF	*Raf1*	-0.299	-0.165	0.253	-0.215	0.116	-0.278	0.055	-0.247	0.052
CRM-1	*Xpo1*	-0.081	-0.079	0.647	-0.266	0.104	-0.171	0.323	-0.136	0.372

CUL3	*Cul3*	0.353	0.026	0.864	-0.092	0.531	-0.025	0.870	0.187	0.169
CYP1A2	*Cyp1a2*	-0.687	-0.123	0.821	-0.924	0.078	-1.176	0.036	-2.285	0.000
CYP1B1	*Cyp1b1*	-0.231	0.206	0.729	-0.544	0.347	-0.757	0.223	-1.001	0.067
CYP27A1	*Cyp27a1*	-0.004	-0.562	0.060	-0.470	0.095	-0.280	0.345	-0.157	0.546
CYP2A6	*Cyp2a5*	0.169	1.206	0.015	1.435	0.003	1.390	0.005	1.171	0.009
CYP2B6	*Cyp2b10*	-0.133	1.602	0.277	4.118	0.008	1.852	0.213	3.296	0.024
CYP2C8	*Cyp2c39*	0.876	0.691	0.457	2.302	0.011	3.947	0.000	4.260	0.000
CYP3A5	*Cyp3a11*	0.415	0.541	0.169	1.047	0.005	0.772	0.049	1.286	0.000
CYP3A7	*Cyp3a13*	0.611	0.099	0.736	0.829	0.003	0.649	0.025	1.007	0.000
CYP4A14	*Cyp4a14*	0.822	2.801	0.015	4.426	0.000	5.538	0.000	6.391	0.000
CYP4A22	*Cyp4a10*	0.830	1.612	0.047	2.220	0.005	3.406	0.000	4.024	0.000
CYP7A1	*Cyp7a1*	0.394	0.316	0.485	0.865	0.045	1.275	0.005	1.275	0.002
CYP8B1	*Cyp8b1*	-0.679	-0.942	0.103	-1.523	0.006	-1.690	0.005	-2.072	0.000
ERK5	*Mapk7*	-0.087	0.035	0.909	0.106	0.709	-0.127	0.682	-0.082	0.765
FASN	*Fasn*	-0.367	-1.511	0.008	-1.297	0.014	-0.592	0.280	-1.562	0.001
FBP1	*Fbp1*	0.117	-0.036	0.851	-0.076	0.681	0.079	0.682	0.065	0.702
Fetuin B	*Fetub*	-0.149	-0.354	0.192	0.252	0.325	-0.229	0.398	-0.330	0.165
FGFR4	*Fgfr4*	-0.490	-0.152	0.465	-0.059	0.765	-0.399	0.058	-0.441	0.017
FOXO1	*Foxo1*	0.603	0.236	0.302	0.322	0.139	0.382	0.094	0.681	0.001
FOXO2	*Foxo3*	0.168	-0.019	0.924	-0.241	0.220	-0.120	0.562	0.015	0.933
FTL	*Ftl1*	0.220	0.118	0.549	0.193	0.301	0.164	0.402	0.214	0.220
FXR	*Nr1h4*	0.190	0.053	0.792	-0.168	0.379	0.148	0.460	0.106	0.548
G6PC	*G6pc*	0.183	-0.646	0.195	-0.285	0.543	0.167	0.733	0.657	0.133
GCLC	*Gclc*	-0.388	0.246	0.368	0.017	0.948	-0.571	0.041	-0.504	0.038
GR	*Nr3c1*	*0.055*	0.191	0.265	-0.069	0.671	0.075	0.664	-0.030	0.845
GSTM1	*Gstm2*	0.284	0.024	0.914	0.454	0.031	0.354	0.109	-0.020	0.917
GSTM2	*Gstm7*	-0.344	-0.306	0.099	-0.338	0.054	-0.342	0.065	-0.385	0.018
HDAC4	*Hdac4*	0.193	-0.153	0.629	0.013	0.966	0.018	0.953	0.128	0.642
HDAC5	*Hdac5*	-0.265	-0.543	0.005	-0.505	0.006	-0.435	0.025	-0.343	0.043
HL	*Lipc*	-0.575	-0.320	0.323	-0.763	0.014	-0.649	0.047	-0.784	0.006
HMGCS2	*Hmgcs2*	0.412	0.102	0.677	-0.066	0.779	0.183	0.455	0.356	0.102
HNF1α	*Hnf1a*	0.203	-0.285	0.066	-0.149	0.306	-0.277	0.074	0.115	0.392
HNF4α	*Hnf4a*	0.338	-0.104	0.665	0.075	0.744	-0.060	0.802	0.316	0.139
HO-1	*Hmox1*	0.117	-0.006	0.985	0.739	0.020	-0.080	0.813	0.241	0.418
IGFBP1	*Igfbp1*	0.377	0.089	0.878	1.118	0.042	0.318	0.581	1.199	0.022
IL-1	*Il1b*	-0.231	-1.102	0.055	-0.394	0.447	-1.195	0.040	-0.497	0.304
INSR	*Insr*	0.507	0.106	0.572	-0.076	0.670	0.153	0.414	0.245	0.141
IRAK	*Irak1*	-0.245	0.026	0.842	0.080	0.521	-0.091	0.486	-0.013	0.908
KEAP1	*Keap1*	0.076	0.009	0.946	-0.022	0.857	0.008	0.950	0.084	0.452
LBP	*Lbp*	0.527	-0.388	0.206	0.429	0.136	0.184	0.543	0.919	0.001
LPL	*Lpl*	-0.185	0.763	0.208	0.938	0.107	-0.668	0.285	-0.623	0.250
LRH-1	*Nr5a2*	0.161	-0.111	0.515	-0.169	0.297	-0.105	0.537	0.106	0.481
MAF	*Maf*	0.259	0.052	0.748	0.210	0.175	0.236	0.146	0.176	0.223
MD-2	*Ly96*	-0.313	-0.231	0.436	-0.253	0.368	-0.798	0.011	-0.412	0.122
MDR1	*Abcb1a*	0.576	1.621	0.014	2.488	0.000	2.378	0.000	2.826	0.000
MDR3	*Abcb4*	0.260	0.286	0.150	0.303	0.110	0.114	0.568	0.310	0.080
MEK5	*Map2k5*	0.099	-0.098	0.545	0.004	0.978	-0.200	0.220	-0.058	0.687
MEKK1	*Map3k1*	-0.017	0.233	0.317	0.167	0.452	-0.016	0.946	0.145	0.486
MGMT	*Mgmt*	0.011	0.128	0.611	0.040	0.868	0.215	0.390	-0.103	0.651
MRP2	*Abcc2*	0.241	0.209	0.333	0.190	0.355	0.132	0.543	0.139	0.470
MRP3	*Abcc3*	0.083	0.231	0.420	0.632	0.020	0.669	0.019	0.092	0.718
MRP4	*Abcc4*	0.073	1.657	0.007	2.157	0.000	0.988	0.108	1.217	0.030
MTTP	*Mttp*	0.098	-0.256	0.280	-0.348	0.121	-0.111	0.638	0.052	0.801

MYD88	*Myd88*	0.040	-0.245	0.162	-0.095	0.564	-0.174	0.318	0.009	0.954
NFκBp65	*Rela*	-0.300	-0.150	0.371	-0.134	0.396	-0.409	0.016	-0.292	0.049
NR0B2	*Nr0b2*	-0.288	-0.450	0.261	-0.159	0.673	-0.627	0.120	-0.554	0.114
NRF2	*Nfe2l2*	-0.139	-0.035	0.828	-0.105	0.491	-0.146	0.365	-0.057	0.687
NTCP	*Slc10a1*	0.248	-0.190	0.627	-0.560	0.135	-0.021	0.958	-0.033	0.924
OATP4	*Slco1b2*	-0.407	0.083	0.834	-0.513	0.178	-0.394	0.326	-0.677	0.054
OSTB	*Slc51b*	0.578	0.160	0.838	1.659	0.023	1.641	0.031	2.322	0.001
PAPSS2	*Papss2*	0.585	0.133	0.603	0.265	0.276	0.622	0.014	0.321	0.157
PEPCK	*Pck2*	0.047	0.229	0.534	0.905	0.008	0.269	0.464	0.047	0.886
PERK	*Eif2ak3*	-0.206	0.109	0.663	-0.187	0.438	-0.587	0.024	-0.329	0.145
PGC-1α	*Ppargc1a*	0.806	-0.172	0.624	0.249	0.452	0.623	0.071	1.064	0.001
PGC-1β	*Ppargc1b*	0.189	0.051	0.824	-0.193	0.380	-0.001	0.998	-0.106	0.605
PKLR	*Pklr*	-0.281	-0.556	0.146	-0.928	0.011	-0.253	0.505	-0.495	0.137
PLTP	*Pltp*	-0.122	-0.973	0.005	-0.269	0.404	-0.156	0.647	-0.992	0.001
PON1	*Pon1*	0.017	-0.150	0.613	-0.284	0.315	-0.070	0.813	-0.008	0.974
PPAR	*Ppara*	0.257	-0.004	0.987	-0.055	0.811	0.114	0.637	-0.037	0.864
PPARγ	*Pparg*	-0.012	0.249	0.483	0.534	0.113	0.024	0.947	-0.111	0.729
PXR	*Nr1i2*	0.805	0.306	0.204	0.498	0.030	0.782	0.001	0.783	0.000
RARα	*Rara*	-0.358	0.048	0.765	0.051	0.739	-0.203	0.217	-0.363	0.013
RXRα	*Rxra*	0.348	-0.026	0.890	-0.001	0.996	0.041	0.828	0.135	0.420
SCD1	*Scd1*	-0.123	-0.561	0.125	-0.670	0.053	-0.278	0.444	-0.438	0.169
SDC1	*Sdc1*	0.257	-0.136	0.497	0.441	0.020	0.208	0.297	0.375	0.035
SITPEC	*Ecsit*	0.136	0.053	0.751	-0.119	0.457	-0.069	0.683	0.111	0.451
SMRT	*Ncor2*	-0.095	-0.029	0.872	0.219	0.197	-0.123	0.495	-0.074	0.643
SOD3	*Sod3*	-0.247	-0.172	0.466	0.149	0.501	-0.314	0.186	-0.498	0.017
SR-BI	*Scarb1*	0.155	-0.154	0.397	0.015	0.930	-0.146	0.422	0.125	0.438
SRC-1	*Ncoa1*	0.269	-0.018	0.903	-0.090	0.507	0.104	0.465	0.194	0.123
SREBP1	*Srebf1*	-0.463	-0.260	0.435	-0.102	0.745	-0.343	0.304	-0.914	0.002
SULT2A1	*Sult2a3*	0.570	0.000	1.000	0.000	1.000	4.322	0.017	6.585	0.001
SUMO1	*Sumo1*	0.202	0.335	0.047	0.142	0.380	0.341	0.044	0.180	0.235
TEBP	*Ptges3*	-0.223	0.113	0.432	0.002	0.991	0.056	0.700	-0.021	0.869
TLR4	*Tlr4*	-0.006	-0.007	0.979	0.146	0.576	0.044	0.875	0.005	0.986
TRAF2	*Traf2*	-0.004	0.326	0.163	0.400	0.071	0.112	0.638	0.206	0.330
TRAF6	*Traf6*	0.420	0.135	0.414	0.082	0.601	0.150	0.365	0.383	0.009
UGT1A1	*Ugt1a1*	0.670	-0.010	0.972	0.310	0.249	0.523	0.063	0.805	0.001
UGT1A9	*Ugt1a9*	0.427	0.722	0.114	0.776	0.075	1.007	0.027	1.313	0.002
VLDLR	*Vldlr*	0.784	0.798	0.260	1.420	0.036	2.706	0.000	2.917	0.000
XAP2	*Aip*	-0.276	-0.335	0.083	0.042	0.815	-0.139	0.465	-0.453	0.008
